# Supplementary material for: Cis-trans isomerization of peptoid residues in the collagen triple-helix
Source: Nat Commun. 2023 Nov 21;14:7571. doi: 10.1038/s41467-023-43469-8 (PMC10663571; doi:10.1038/s41467-023-43469-8)
Supplement: Supplementary file 1 — Supplementary Information [file 41467_2023_43469_MOESM1_ESM.pdf]

Supplementary Information

Cis-trans isomerization of peptoid residues in the collagen triple-helix

Rongmao Qiu[1,2], Xiaojing Li[1,2], Kui Huang[1,2], Weizhe Bai[3], Daoning Zhou[1,2], Gang Li[3,*], Zhao Qin[4,*], and Yang Li[1,2,3,*]

[1]Guangdong Provincial Engineering Research Center of Molecular Imaging, the Fifth Affiliated Hospital, Sun Yat-sen University, Zhuhai, 519000, China

[2]Guangdong-Hong Kong-Macao University Joint Laboratory of Interventional Medicine, the Fifth Affiliated Hospital, Sun Yat-sen University, Zhuhai, 519000, China

[3]Cardiac Surgery and Structural Heart Disease Unit of Cardiovascular Center, the Fifth Affiliated Hospital, Sun Yat-sen University, Zhuhai, 519000, China

[4]Department of Civil & Environmental Engineering, College of Engineering & Computer Science, Syracuse University, Syracuse, New York 13244, USA

*Correspondence to:

Gang Li: gangli73@163.com; Zhao Qin: zqin02@syr.edu; Yang Li: liyang266@mail.sysu.edu.cn

**Section 1:** Supplementary Figures
Supplementary Fig. 1. CD spectra of X-CMPs and T_m curves under the heating rate of 0.1 °C min^{-1}
Supplementary Fig. 2. Differential scanning calorimetry scans of X-CMPs
Supplementary Fig. 3. Thermodynamic parameters of triple-helix folding for N2pic- and Nme$_2$ae-CMPs in different protonation states
Supplementary Fig. 4. MD simulation for the distribution of dihedral angles of X residues
Supplementary Fig. 5. Refolding kinetic curves and rate constants of X-CMPs
Supplementary Fig. 6. CD refolding curves of Nleu-, Nchx-, Nphe-CMP in PBS and NaOH solution
Supplementary Fig. 7. Schematic of CMP-collagen hybridization
Supplementary Fig. 8. CD unfolding curves of N2pic3-CMP in 1 mM HCl solution
Supplementary Fig. 9. In vivo targeting of denatured collagen by Cy5-N2pic3-CMP
Supplementary Fig. 10. In vivo targeting of denatured collagen in the fibrotic scar of myocardial infarction with Cy5-X7
Supplementary Fig. 11. Relationship between the stability of X-CMPs and $K_{cis/trans}$ of X residues
**Section 2:** Supplementary Methods
Materials
Solid phase synthesis
Cleavage protocols
Purification and mass spectrometry
Circular dichroism spectroscopy
Differential scanning calorimetry
Molecular dynamics simulations
Gelatin binding assays
In vivo skeleton targeting
The myocardial infarction model and in vivo targeting
Cryosections and immunofluorescence staining
Tissue clearing and light sheet fluorescence microscopy
**Section 3:** Synthesis and Characterization of Ac-X-OMe Model Compounds
**Section 4:** CD and MALDI of All Peptides
Characterizations of X-CMP peptides
Characterizations of X-PP5 peptides (arranged according to Fig. 4e)
**Supplementary References**

Section 1: Supplementary Figures

a

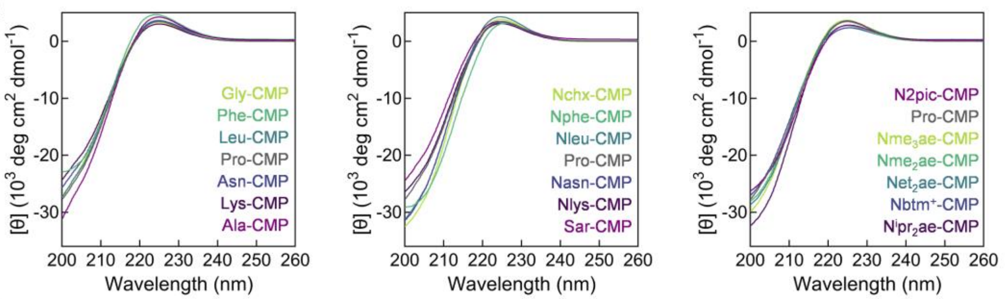

b

	X	$K_{cis/trans}$	$[\theta]_{max}$	λ_{max}		X	$K_{cis/trans}$	$[\theta]_{max}$	λ_{max}		X	$K_{cis/trans}$	$[\theta]_{max}$	λ_{max}
	Pro	0.26	3488	225.0		Pro	0.26	3488	225.0		Pro	0.26	3488	225.0
	Gly	< 0.01	3389	224.8		Sar	0.40	3493	224.7		N2pic	3.78	2846	225.5
	Asn	<0.01	3617	225.1		Nleu	0.43	4312	224.5		Nme_2ae	5.32	2739	224.9
AA	Lys	< 0.01	3050	225.0	N-gly (K < 1)	Nchx	0.44	3856	224.7	N-gly (K > 1)	Net_2ae	5.49	3479	224.8
	Ala	< 0.01	4268	224.9		Nphe	0.64	3182	226.0		$Nbtm^+$	5.76	2383	225.5
	Leu	< 0.01	3660	224.8		Nasn	0.73	3388	225.3		Nme_3ae	6.10*	3698	224.7
	Phe	< 0.01	4688	224.1		Nlys	0.80	3127	225.0		N^ipr_2ae	10.85	3570	224.9

c

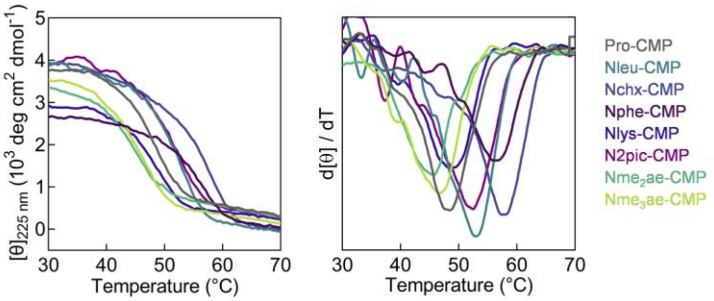

d

X-CMP	$K_{cis/trans}$	Heating rate: 0.5 °C min^{-1}		Heating rate: 0.1 °C min^{-1}		$\Delta\Delta T_m$ (°C) (0.5 vs. 0.1 °C min^{-1})
		T_m (°C)	ΔT_m (°C) vs. Pro	T_m (°C)	ΔT_m (°C) vs. Pro	
Pro-CMP	0.26	55	0	49	0	0
Nleu-CMP	0.43	59	4	53	4	0
Nchx-CMP	0.44	64	9	58	9	0
Nphe-CMP	0.64	61	6	57	8	-2
Nlys-CMP	0.80	55	0	48	-1	1
N2pic-CMP	3.78	59	4	53	4	0
Nme_2ae-CMP	5.32	52	-3	46	-3	0
Nme_3ae-CMP	6.10	53	-2	47	-2	0

Supplementary Fig. 1. a The full CD spectra of X-CMPs featuring amino acids, weakly trans-biased N-glys, and strongly cis-demanding N-glys as the guest X unit, recorded in PBS solution (except for N2pic-CMP, which was measured in 1 mM HCl, pH 3.0). **b** The $K_{cis/trans}$ values of the X residues and the $[\theta]_{max}$ (unit: deg cm^2 dmol^{-1}) as well as λ_{max} values (unit: nm) of the corresponding X-CMP CD curves. **c** The CD thermal unfolding curves and their first derivatives of the X-CMPs recorded under the slow heating rate of 0.1 °C min^{-1}. **d** The difference in T_m between Pro-CMP and each N-gly featuring X-CMP remains virtually unchanged under different CD heating rates (i.e., $\Delta\Delta T_m$ = 0 for 0.5 vs. 0.1 °C min^{-1}), confirming that the high stability of these peptoid X-CMPs is not artifacts caused by our heating rate.

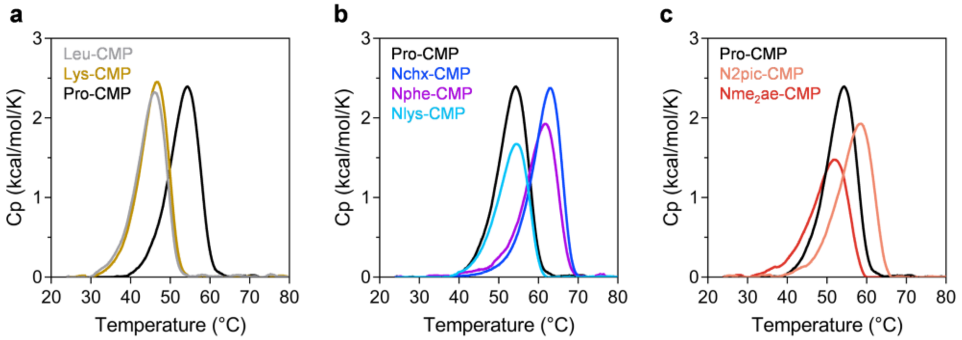

Supplementary Fig. 2. Differential scanning calorimetry (DSC) scans of X-CMPs featuring amino acid (**a**), weakly trans-favoring N-gly (**b**), and strongly cis-biased N-gly (**c**) guest residues. For all DSC curves, the endothermic change is in positive direction.

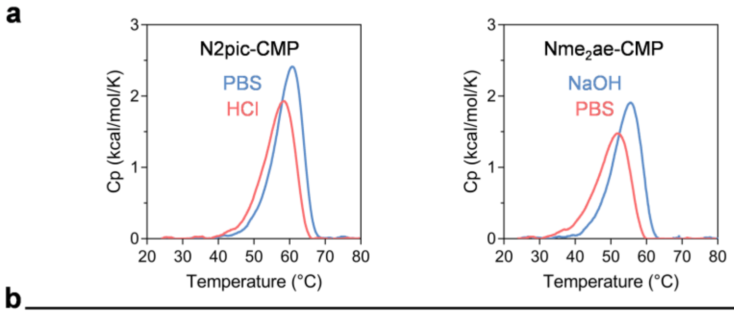

b

X	pH	Charge	$K_{cis/trans}$	T_m (°C)	ΔG* (kcal mol^{-1})	ΔH* (kcal mol^{-1})	$-T\Delta S$* (kcal mol^{-1})
N2pic	3.0	H^+	3.78	59	-11.2	-20.8	9.6
	7.4	neutral	0.72	62	-11.4	-23.9	12.5
Nme_2ae	7.4	H^+	5.32	52	-10.8	-16.8	6.0
	11.5	neutral	0.79	57	-11.0	-19.1	7.7

Supplementary Fig. 3. a DSC scans of N2pic-CMP in 1 mM HCl solution (pH 3.0, red) and PBS buffer (pH 7.4, blue) as well as Nme_2ae-CMP in PBS buffer (pH 7.4, red) and 3.5 mM NaOH solution (pH 11.5, blue). **b** Thermodynamic parameters ΔG, ΔH, and $T\Delta S$ of X-CMP triple-helix folding with opposite cis-trans propensities of N2pic and Nme_2ae in different protonation states. T_m values were measured by CD. For all DSC curves, the endothermic change is in positive direction. *: values were determined by DSC and reported here at T = 55 °C.

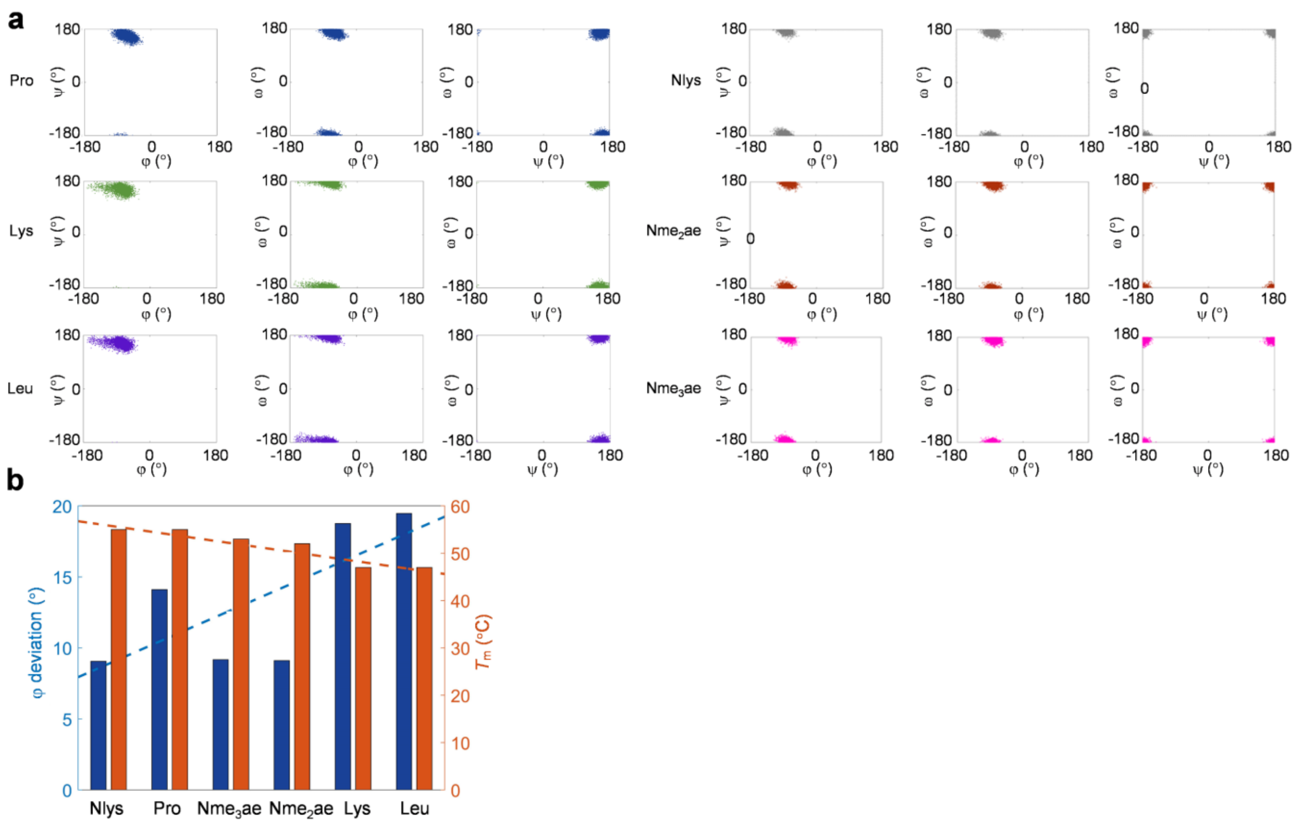

Supplementary Fig. 4. a The distribution of the φ, ψ, ω dihedral angles of various X residues within the X-CMP triple-helix during the structural relaxation process in MD simulation: N-glys, including the strongly cis-favoring Nme_2ae and Nme_3ae, have a distribution range of dihedral angles similar to Pro, while amino acids Leu and Lys have a notably wider variation of φ during the relaxation. **b** The X-residues with a larger φ deviation seemed to form X-CMP triple-helices with lower stabilities.

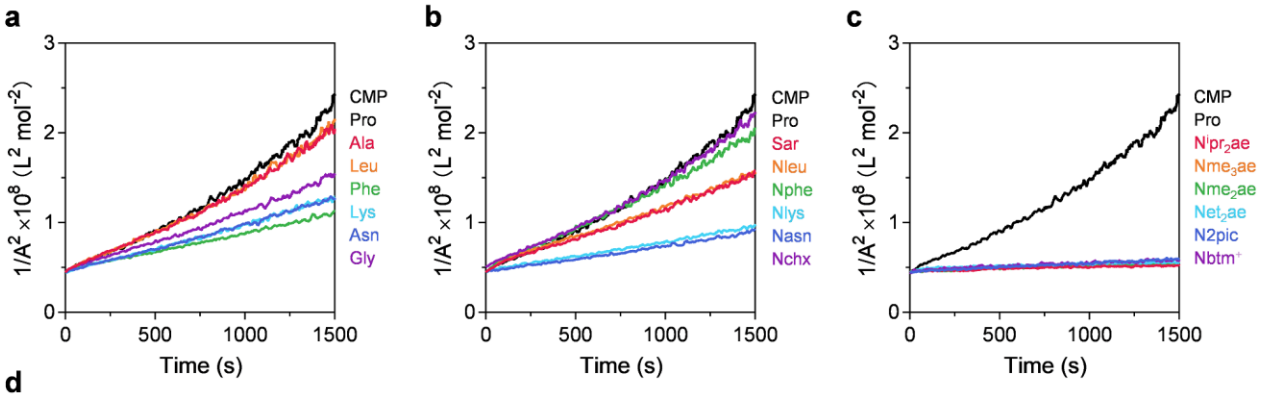

d

Residue X	$K_{cis/trans}$	$t_{1/2}$ (min)	k_3 ($\times$ 10^3 M^{-2} s^{-1})	Residue X	$K_{cis/trans}$	$t_{1/2}$ (min)	k_3 ($\times$ 10^3 M^{-2} s^{-1})	Residue X	$K_{cis/trans}$	$t_{1/2}$ (min)	k_3 ($\times$ 10^3 M^{-2} s^{-1})
Pro	0.26	21.8	20.0	Pro	0.26	21.8	20.0	Pro	0.26	21.8	20.0
Gly	< 0.01	28.5	11.7	Nchx	0.44	24.9	18.3	N2pic	3.78	>>120	1.5
Ala	< 0.01	22.5	16.7	Sar	0.40	29.9	11.7	Nme$_2$ae	5.32	>>120	0.8
Leu	< 0.01	22.4	18.3	Nleu	0.43	29.9	11.7	Net$_2$ae	5.49	>>120	0.8
Phe	< 0.01	46.1	6.7	Nphe	0.64	22.6	16.7	Nbtm$^+$	5.76	>>120	1.3
Asn	< 0.01	36.7	8.3	Nasn	0.73	60.4	5.0	Nme$_3$ae	6.10	>>120	1.0
Lys	< 0.01	35.8	10.0	Nlys	0.80	59.2	5.0	N^{i}pr$_2$ae	10.85	>>120	0.8

Supplementary Fig. 5. Processed refolding kinetic curves of X-CMPs derived from Fig. 5e-g featuring amino acids (**a**), weakly trans-biased N-glys (**b**), and strongly cis-demanding N-glys (**c**) as the guest residue. See Supplementary Methods for details. **d** The refolding half-time values ($t_{1/2}$) and the third-order rate constants (k_3) of the each X-CMP.

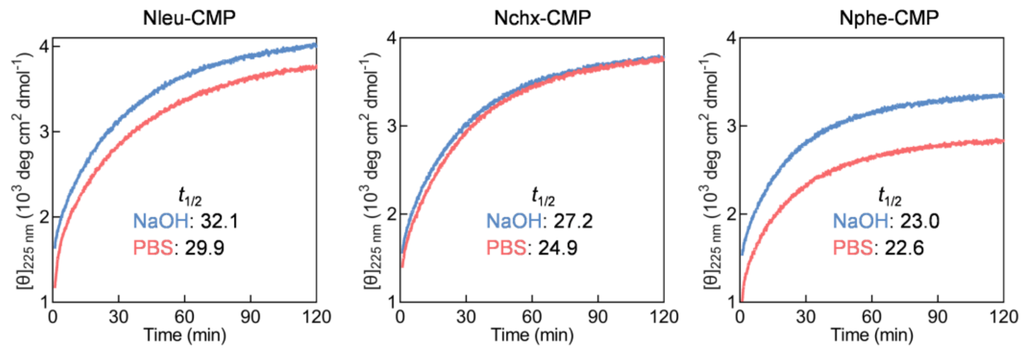

Supplementary Fig. 6. With sidechains that cannot be protonated, Nleu-CMP, Nchx-CMP, and Nphe-CMP had highly similar CD refolding curves and $t_{1/2}$ values (unit: min) in PBS (pH 7.4, red) and 3.5 mM NaOH solution (pH 11.5, blue) at 4 °C.

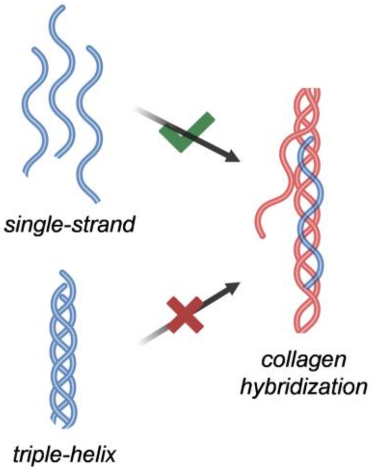

Supplementary Fig. 7. The CMP single-strands, but not the triple-helix can hybridize with denatured collagen through triple-helix formation. But the single-stranded CMPs (monomers) can spontaneously and gradually form peptide homotrimers in solution, thereby losing their driving force for collagen hybridization. Consequently, a CMP solution usually needs to be heated (e.g., at 80 °C) to dissociate the peptide into monomers immediately before a hybridization application (e.g., tissue staining or in vivo injection).

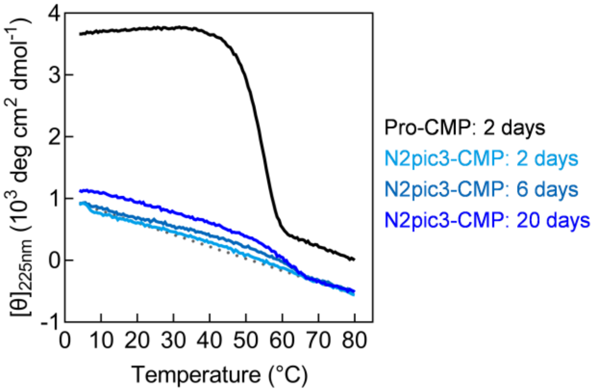

Supplementary Fig. 8. The CD thermal unfolding curves of N2pic3-CMP (150 μM in 1 mM HCl solution) after incubation at 4 °C for 2, 6, and 20 days. Compared to Pro-CMP, all three curves showed little sign of triple-helix formation.

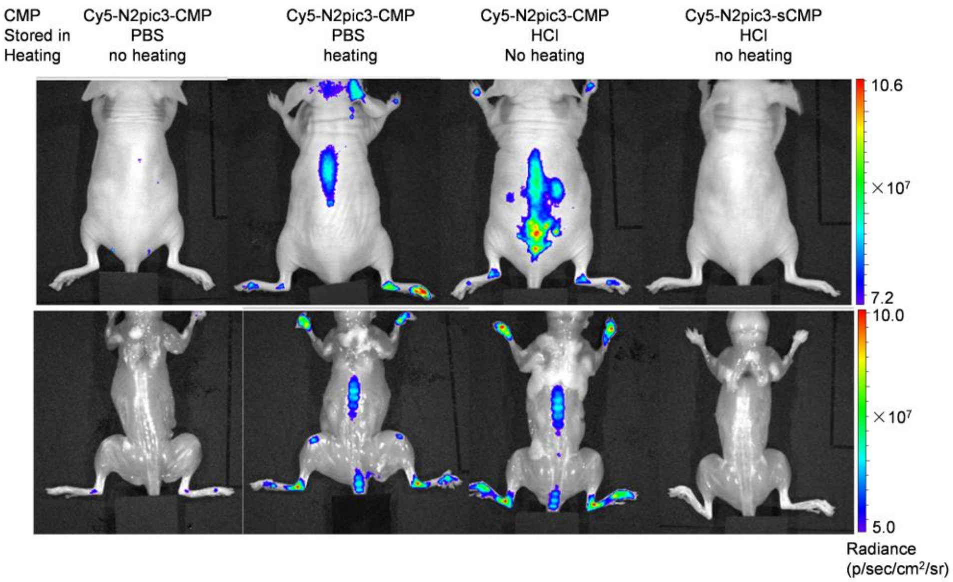

Supplementary Fig. 9. In vivo targeting of the physiological level of denatured collagen in skeletal tissue by Cy5-N2pic3-CMP without preheating. Whole-body near-infrared fluorescence images of four nude mice injected intravenously with 1 nmol of Cy5-N2pic3-CMP or the sequence-flipped control peptide Cy5-N2pic3-sCMP. The Cy5-N2pic3-CMP were either kept in 1 mM HCl until being directly injected without preheating or stored in PBS and injected with and without preheating. The in vivo fluorescence images showed clear skeletal uptake of only the Cy5-N2pic3-CMP single-strands (top row). Near-infrared fluorescence images of nude mice after skin removal showing uptake of Cy5-N2pic3-CMP in the spine and joints (bottom row). All images were representative of similar results obtained from three independent experiments.

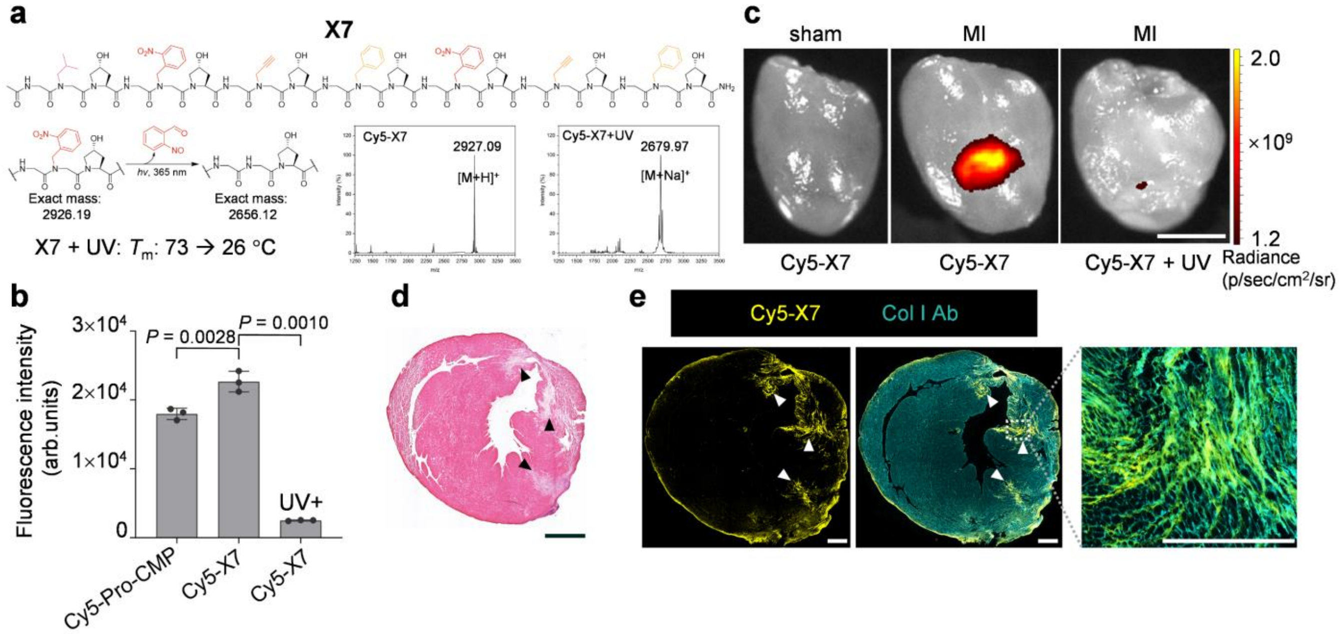

Supplementary Fig. 10. In vivo targeting of denatured collagen in the fibrotic scar of myocardial infarction (MI) with CMP X7. **a** The structure of CMP X7, featuring no Pro but various N-glys at all Xaa positions, whose T_m value reduces from 73 to 26 °C after photo-triggered cleavage of the two nitrobenzyl sidechains by UV irradiation. MALDI-MS for Cy5-X7, calculated 2927.19 $[M+H]^+$, observed: 2927.13 $[M+H]^+$; after UV exposure, Cy5-X7+UV, calculated 2679.13 $[M+Na]^+$, observed: 2679.97 $[M+Na]^+$. **b** Fluorescence of the gelatin films treated with Cy5-labeled X-CMPs, demonstrating Cy5-X7's higher affinity to denatured collagen which was diminished for the UV-irradiated control peptide (n = 3 independent samples). data were represented as mean ± standard deviation and analyzed with one-way analysis of variance (ANOVA), followed by *post-hoc* Tukey tests. **c** Near-infrared fluorescence images of the hearts harvested from mice 8 days after MI showed a robust fluorescence signal compared to the sham group, 1 h post intravenous injection of the preheated Cy5-X7, but not the UV-irradiated control peptide (n = 3 mice). **d** Hematoxylin and eosin staining of the slides of the MI heart sectioned from (**c**) indicated the presence of infarction (arrowheads), n = 3 mice. **e** Fluorescence micrographs of the cryosections of the MI heart from the mouse injected with Cy5-X7 in vivo in (**c**), co-stained with an anti-collagen I antibody, showing specific binding of Cy5-X7 to denatured collagen co-localizing with the collagen I antibody within the infarcted fibrotic areas (white arrowheads), n = 3 mice. Scale bars: 5 mm (**c**), 1 mm (**d**), and 0.5 mm (**e**).

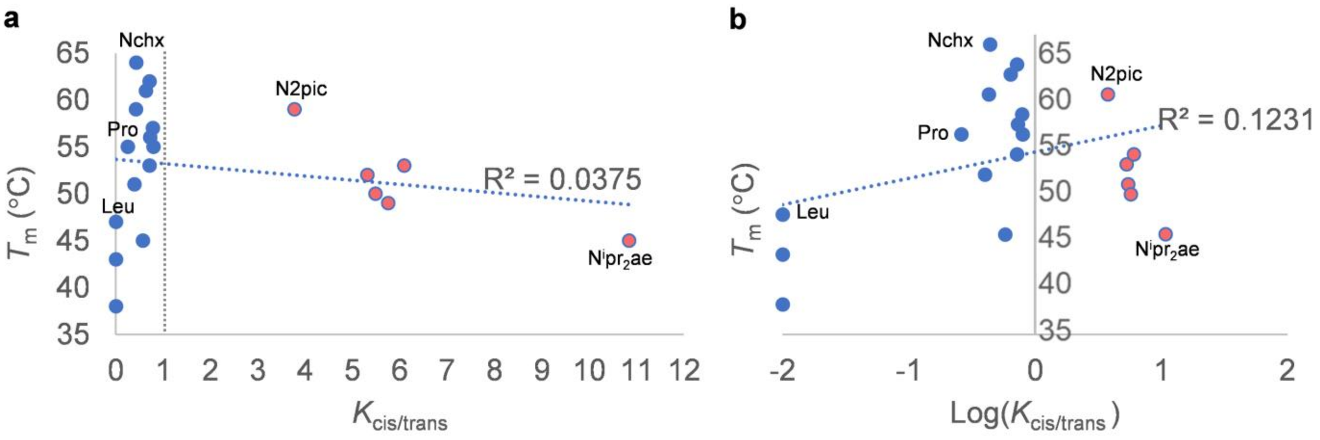

Supplementary Fig. 11. Whether linearly (left, **a**) or logarithmically (right, **b**), no strong correlation was found between the X residues' $K_{cis/trans}$ values and the triple-helix stability of the X-CMPs.

Section 2: Supplementary Methods

Materials

All materials and reagents were purchased from commercial sources and used without further purification. The solvents, resin, Fmoc amino acids, and reagents used in the synthesis and purification of the peptides and peptoids were purchased from the suppliers listed in the table below. The water used for peptide preparation and purification was Milli-Q water with a resistivity of 18.2 MΩ.cm.

General reagents	Suppliers	Catalog number
N, *N*-Dimethylformamide (DMF)	Aladdin	D112002-500ml
Dimethyl sulfoxide (DMSO)	Aladdin	D106264-50g
Methylene Chloride (DCM)	Aladdin	D116146-4L
Acetonitrile (MeCN)	Macklin	A800362-4L
Ethyl ether	Guangzhou chemical reagent factory	20201205 10
Rink Amide AM resin	Tianjin Nankai Hecheng Science & Technology	GRAV0921
Fmoc-Gly-OH	Aladdin	F103019-100g
Fmoc-Pro-OH	Macklin	F809731-100g
Fmoc-Hyp(tBu)-OH	Aladdin	F117058-5g
Fmoc-Lys(Boc)-OH	GL Biochem	36802
Fmoc-Phe-OH	GL Biochem	35701
Fmoc-Ala-OH	GL Biochem	35001
Fmoc-Asn-OH	GL Biochem	35102
Fmoc-Leu-OH	GL Biochem	35501
Fmoc-Sar-OH	Macklin	F809960-25g
Fmoc-Flp-OH	GL Biochem	24175
Fmoc-6-Ahx-OH	Energy Chemical	A0703220250-A01
Fmoc-(2-nitrobenzyl)Gly-OH	GL Biochem	22075
Piperidine	Detian Fine Chemicals	20191105
Bromoacetic acid	Macklin	B802565-25g
N, *N*'-Diisopropylcarbodiimide (DIC)	Aladdin	D106162-100ml
O-(7-Azabenzotriazol-1-yl)-*N*, *N*, *N*', *N*'-tetramethyluronium hexafluorophosphate (HATU)	Aladdin	H109327-100g
1-Hydroxy-7-azabenzotriazole (HOAt)	Macklin	H811122-100g
N-Ethyldiisopropylamine (DIEA)	EMD Millipore	S1807494945
Acetic acid (AcOH)	Aladdin	A116174-500ml
Trifluoroacetic acid (TFA)	Macklin	T818782-500ml
Triisopropylsilane (TIS)	Macklin	T819181-100ml
Methyl iodide (MeI)	Energy Chemical	W6107051000-A01

Solid phase synthesis

Coupling and Fmoc deprotection

All sequences were prepared on Rink Amide AM resin (substitution level: 0.37 mmol g^{-1}) using the Fmoc/*t*Bu strategy for solid phase synthesis.[1] Typically, 27 mg of resin (containing 10 µmol of reaction sites) was used to prepare one host-guest peptide/peptoid sequence. First, the Rink Amide AM resin was swelled in 0.5 mL of DMF for over 30 min. Fmoc-deprotection was carried out by treating the resin with 20% piperidine in DMF (V/V) for 20 min. Following each reaction, the resin was drained and washed with DMF three times (3×10 mL). Each amino acid residue was coupled by agitating the resin with a solution of Fmoc amino acid (50 µmol), HATU (50 µmol, 19 mg), HOAT (50 µmol, 6.8 mg), and DIEA (75 µmol, 13 µL) in 0.4 mL of DMF for over 3 hours. All coupling and deprotection reactions were monitored by the Kaiser and chloranil tests.

Acylation and displacement (peptoid residues)

Sar and Nnbz were coupled by HATU chemistry, other peptoid residues were incorporated on-resin by the submonomer method using bromoacetic acid and N,N'-diisopropylcarbodiimide (DIC) in DMF reported by Zuckermann et al.[2]

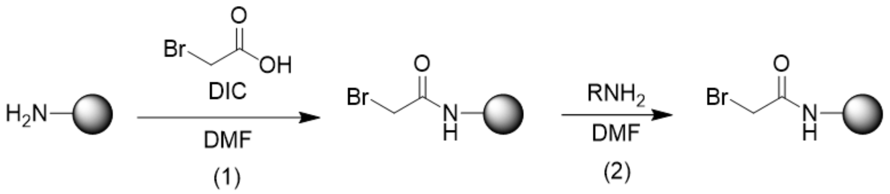

(1) Acylation reactions were performed by the addition of a solution of bromoacetic acid (100 µmol, 13.9 mg) and DIC (98 µmol, 15.2 µL) in DMF (0.5 mL) to 10 µmol of resin-bound amine. Reaction mixtures were agitated at room temperature for 30 min, drained, and washed with DMP (3×10 mL). Each acylation was repeated once more before washing (double-coupling). **(2)** Each displacement reaction was performed by the addition of a primary amine (200 µmol) in 0.5 mL of DMF, followed by agitation overnight at room temperature. For poorly nucleophilic amines such as the quaternary ammonium-containing amine, 0.1 eq of KI was added to the reaction mixture, and the displacement time was extended up to 24 hours.[3] Hydrochloride amine salts were free-based *in situ* with 21 equiv. DIEA. Following the displacement, the resin was drained and washed with DMF (3×10 mL). The reaction was monitored by the chloranil test. In the table below, the primary amine used to create each peptoid residue was listed, and special reaction conditions and observations were noted. In general, the next amino acid following a peptoid residue (e.g., Fmoc-Gly-OH or Fmoc-Pro-OH) was coupled with full completion using the above-mentioned 5 eq HATU protocol.

Residue	R-NH_2 Reagent	Supplier	Catalog number	Note
Nakn	Propargylamine	Aladdin	P103552-5G	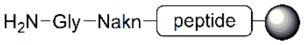: in the Kaiser test, resin beads and the solution do not turn dark blue despite the presence of primary amine
Nasn	Glycinamide hydrochloride	Aladdin	G106202-5g	The displacement reaction was carried out in 1 mL DMSO.
Nchx	Cyclohexanemethylamine	Macklin	A835490-5ml	
Nleu	Isobutylamine	Aladdin	I103753-5ml	
Nlys	*N*-Boc-1,4-butanediamine	Aladdin	B102317-5g	
Nphe	Benzylamine	Aladdin	B108477-100ml	
N2pic	2-picolylamine	Aladdin	P107146-100g	
Nme$_2$ae	N, N-Dimethylethylenediamine	Aladdin	D105347-25ml	
Net$_2$ae	N, N-Diethylethylenediamine	Aladdin	D105350-25ml	
N^{i}pr$_2$ae	N, N-diisopropylethylenediamine	Aladdin	D109869-25ml	
Nme$_3$ae	(2-Aminoethyl) trimethylammonium chloride	Sigma-Aldrich	284556-1G	The displacement reaction was carried out in a 1 mL mixture of DMSO and water (1:1, v/v).

On-resin synthesis of triazolium peptoid side chains

Benzylazide (60 μmol, 7.5 μL, Macklin, B830561-1g) was dissolved in 0.5 mL of DMF along with $[Cu(MeCN)_4]PF_6$ (10 μmol, 3.7 mg, Sigma-Aldrich, 346276) and TBTA (20 μmol, 10.6 mg, Sigma-Aldrich, 678937). This solution was added to the resin-bound alkyne-containing peptide Nakn-CMP (5 μmol), and the mixture was agitated at room temperature overnight.[4] The resin was drained and washed with DMF (5×10 mL) before mixing with a solution of a MeI (75 μmol, 4.7 μL) in MeCN (0.75 mL). The resulting mixture was stirred at 60 °C for 24 hours then drained and washed with DMF (5×10 mL).

N-terminal acetylation

The N-terminal amine of each sequence was acetylated by combining the resin (5 μmol) with a solution of AcOH (50 μmol, 3 μL), HATU (50 μmol, 19 mg), HOAt (50 μmol, 6.8 mg), and DIEA (75 μmol, 13 μL) in 0.5 mL of DMF. The mixture was agitated at room temperature for 3 hours. The resin was washed with DMF (3×10 mL).

Cleavage protocols

The resin (5 µmol) was washed with DCM (5×10 mL) and drained, then treated with 1 mL mixture of TFA/TIS/H_2O (95:2.5:2.5) for 3 hours with stirring. Subsequently, the resin was washed with 0.5 mL of TFA twice. After the resin was filtered, the TFA cleavage solution was collected and evaporated under a stream of nitrogen down to approximately 0.5 mL. Crude products were precipitated by adding 5 mL of cold ethyl ether to the TFA solution. The white solid was centrifuged, and the supernatant was decanted.

Purification and mass spectrometry

All peptides were purified by reverse-phase high-performance liquid chromatography (HPLC) on a semi-preparative column (Agilent ZORBAX StableBond 300 C18), using a mixture of water (A) and acetonitrile (B) (linear gradient: 5% to 35% acetonitrile in 25 mins). Both eluents A and B contained 0.1% TFA. The column oven was heated to 85 °C to unwind the triple-helix during purification. The flow rates used for semi-preparative were 4 mL min^{-1}. The pure fractions were lyophilized and were reconstituted in Milli-Q water as stock solutions to be used for structural characterizations. Peptide concentrations in the stock solutions were determined by measuring the ultraviolet (UV) absorbance of the solutions at 214 nm for the collagen mimetic host-guest sequences (extinction coefficient: 2200 M^{-1} cm^{-1} per peptide bond) or 280 nm for the Tyr-containing polyproline host-guest sequences (extinction coefficient: 1280 M^{-1} cm^{-1} per tyrosine residue) on an IMPLEN NP80 Nano drop using a quartz cell with a 1 cm cell path length.

All purified peptides were verified by mass spectrometry. Mass spectra were obtained on a Shimadzu 8020 matrix-aided laser analytical ionization-time of flight (MALDI/TOF) mass spectrometer (see Section 4).

Circular dichroism (CD) spectroscopy

CD spectra of the peptides were recorded in quartz cells with a path length of 0.1 cm, on a JASCO J-1500 CD spectrophotometer. Before experiments, peptide stock solutions were diluted to 150 µM in 1×PBS (pH: 7.4 for the triple-helical peptides) or 5 mM phosphate buffer (pH: 7.0 for the polyproline host-guest peptides) and heated at 85 °C for 5 min followed by incubation at 4 °C for at least 48 hours. Sequences containing guest residues with Lys, Nme_2ae, Net_2ae, and $Nipr_2ae$ were also diluted to 150 µM in 3.5 mM NaOH solution (pH 11.5). Sequences containing N2pic residue were also diluted to 150 µM in 1 mM HCl solution (pH 3.0).

CD spectra were scanned at 4 °C (for triple-helical peptides) or 25 °C (for polyproline host-guest peptides) using the following parameters: bandwidth, 5 nm; digital integration time, 16 s; scanning speed, 20 nm min^{-1}; data pitch, 0.1 nm. All reported CD spectra were the average of two independent scans and were corrected from the blank buffer background.

Thermal melting curves were obtained by monitoring the ellipticity of each peptide solution at 225 nm from 4 °C to 80 °C at a heating rate of 0.5 °C min^{-1} or 0.1 °C min^{-1}. The mean residue ellipticity (MRE, [θ]) was calculated using the equation:

$$[\theta] = (\theta \times m) / (c \times l \times n) \tag{1}$$

where θ is measured ellipticity (mdeg), m is molecular weight (g mol^{-1}), c is the peptide concentration (mg mL^{-1}), l is the path length of the cuvette (mm), and n is the number of amino-acid/peptoid residues in the peptide. The derivative of a melting curve was generated using the JASCO Spectra Manager software (Version 2.15.01), and the temperature at the minimum of the derivative curve was defined as the melting temperature (T_m). Each T_m value reported in this study was averaged from two CD thermal unfolding experiments, in which the difference between the two measured T_m values was less than 1 °C for all CMP host-guest peptides.

Folding curves of the collagen triple helices during the cooling process were obtained by denaturation and cooling experiments. Peptide solutions (150 µM) were heated to 80 °C and gradually cooled down to 4 °C with a rate of 0.5 °C min^{-1}, while the ellipticity at 225 nm was monitored by CD spectroscopy.[5]

For refolding studies, 150 µM of each peptide solution was heated at 85 °C for 5 min followed by incubation at 4 °C for at least 48 hours. The initial ellipticity for collagen triple helices was recorded at 225 nm at 4 °C and was taken as completely assembled (θ_f). Refolding curves of triple helices were obtained by the following steps: (1) heated 150 µM peptide solutions in a 1 mm cuvette at an 85 °C water bath for 10 min, (2) then immediately placed the cuvette in the CD instrument which was pre-cooled to 4 °C and the ellipticity at 225 nm was monitored by CD spectroscopy for 120 min. Since the heated peptide solutions need about one min to equilibrium to 4 °C, the ellipticity in the first one minute (deadtime) was discarded.[6] The fraction of folded peptide (F) is defined as:

$$F = (\theta_t - \theta_u) / (\theta_f - \theta_u) \tag{2}$$

where θ_t represents the ellipticity at time t, θ_u is the ellipticity of the unfolded form and θ_f is the ellipticity of the folded form. θ_u was defined as the ellipticity measured at 1 min at 4 °C.[6] θ_f was measured directly before denaturation at 4 °C. To compare the data of different peptides, refolding half-time ($t_{1/2}$) at which F = 0.5 was determined. We further analyzed the refolding data according to Brodsky and colleagues, who showed that CMP refolding follows a third-order kinetics model at low concentrations (< 0.18 mM).[7,8] To estimate the rate constants of the X-CMP refolding, we first calculated the concentration of monomer peptide [A_t] at time t (s) as:

$$[A_t] = (1-F) \times [A_0] \tag{3}$$

where [A_0] represents the initial monomeric peptide concentration (assumed as 1.5×10^{-4} mol L^{-1}). Using the data from the refolding kinetic curves, the third-order rate constant (k_3) of each X-CMP can be obtained by finding the slope of the line that fits the plot of $1/[A_t]^2$ against t (Supplementary Fig. 5),[7] based on the following equation:

$$1/[A_t]^2 = 1/[A_0]^2 + 6k_3t \quad (4)$$

Triple-helix folding curves of N2pic3-CMP triggered by pH changes

The ellipticity of an N2pic3-CMP solution (150 µM in 1 mM HCl solution, 200 µL, pH 3.0) at 225 nm was monitored by CD spectroscopy at 4 °C using the time course measurement for 10 min. Subsequently, 0.23 M NaOH solution (1 µL) was added to the peptide solution in the CD sample cuvette to adjust the pH level to approximately 7.4. The ellipticity was monitored by CD spectroscopy immediately. Five minutes later, 0.23 M HCl solution (1 µL) was added to the same peptide solution to bring the pH level back to 3.0 and the ellipticity was monitored immediately. Ten minutes later, 0.23 M NaOH solution was added again and the ellipticity was monitored for five minutes.

Differential scanning calorimetry (DSC)

All DSC studies were designed according to Matthew D. Shoulders *et al.*[9,10] and carried out using an Automated MicroCal PEAQ-DSC instrument with a heating range from 20 to 80 °C, scan rate: 0.5 °C min^{-1}, and feedback set to "none" for enhanced signal-to-noise ratio. Before experiments, peptide stock solutions were diluted to 250 µM in 1×PBS (or1 mM HCl / 3.5 mM NaOH solution for specific X-CMPs), and incubated at 4 °C for at least two days before measurement. Each sample was degassed before being added to the sample cell. Measurements were repeated two times and averaged for each peptide. For each sample, the corresponding reference scan was subtracted from the sample scan. The melting temperature (T_m) of the CMPs in DSC was defined as the temperature where the maximum heat capacity (C_p) was observed. The change in enthalpy ΔH was obtained by direct integration of the DSC exotherms. The change in entropy ΔS (at T_m) was calculated with the equation:

$$T_m = \Delta H / (\Delta S + R \cdot \ln(0.75c^2)) \quad (5)$$

where c is the concentration of monomeric peptide.[9,11] Because this model assumed $\Delta C_p = 0$ for triple-helix unfolding (an approximation commonly employed for collagen triple-helix), ΔH and ΔS are independent of temperature.[12,13] The change in Gibbs free energy ΔG was then calculated with the equation:

$$\Delta G = \Delta H - T\Delta S \quad (6)$$

where T = 55 °C, which was the average of the T_m values for the X-CMPs.

Fully atomistic molecular dynamics (MD) of collagen sequences

We started by using the fully equilibrated tropocollagen structure of $[(GPO)_5]_3$ as the template, we used psfgen as a tool of the NAMD package to remove a certain amount of residues from the N- and C-terminus and added

acetylated N-terminus and amidated C-terminus (-$CONH_2$) to each of the collagen chains to obtain the initial molecular structure.[14] Classical, fully atomistic molecular dynamics (MD) simulations were used to fully equilibrate the collagen in the explicit water box with a dimension of 10.5×5×5 nm^3 composed of 100 mM NaCl and TIP3P water molecules. The simulation was carried out with a CHARMM27 all-atom energy force field for collagen peptides[15] and with a CHARMM General Force field (CGenFF)[16] for the peptoid substitution. To incorporate the n→π* interactions for the peptoid residues, we modified the CGenFF parameters by reducing the energy across ω torsion angles (lowering each ω force constant from 2.50 to 2.15 kcal/mol) to lower the cis/trans barrier relative to proteins, according to Weiser *et al.*, who showed that the peptoid conformations computed with the modified-CGenFF reproduce the quantum mechanical potential energy profiles.[17] The net charge of the system was zero by adjusting the ratio between cation and anion and each ion was initially distributed randomly in water with at least 5 Å from the collagen structure. The simulation time step was 2 fs with a rigid bonds model for all the covalent bonds between hydrogen atoms and other heavy atoms. We used the particle mesh Ewald (PME) function with a grid width <1 Å to calculate the electronic interaction as an efficient method to accurately include all the long-distance electrostatic interactions. For the initial run, each of all the backbone atoms was constrained in space by elastic springs with a stiffness of 5 kcal mol^{-1} $Å^{-2}$ in all three directions, while the side chain atoms and solvent molecules were free to move. We ran 10,000 steps of energy minimization by using a conjugate gradient and line search algorithm followed by 2 ns dynamics run for initial equilibrium. The dynamics simulation was performed in the NPT (constant pressure of 1 atm and constant temperature of 310 K) ensemble controlled by the Langevin dynamics to reach a constant pressure and temperature (with 5 ps^{-1} damping coefficient for temperature, 100 fs oscillation period and 50 fs damping time scale for pressure control) with the shrinkable volume during the run. After the initial run, we restarted the simulation, released all the constraints on the backbone atoms, and continued using the NPT ensemble for 100 ns to get fully equilibrated collagen structures and examined the variation of dihedral angles (φ, ψ, ω) during the simulation. The simulations were performed by using a NAMD package (v2.12) in the local Linux workstation with multiple graphics processing units (NVIDIA RTX 3060) support through Compute Unified Device Architecture (CUDA v11.4).[14]

Gelatin binding assays

Cy5-labeled peptides Cy5-Pro-CMP, Cy5-N2pic3-CMP, and Cy5-N2pic3-sCMP [sequences: Cy5-Ahx-$(GlyProHyp)_7$, Cy5-Ahx-GlyProHyp-$(GlyN2picHyp)_3$-$(GlyProHyp)_3$, Cy5-Ahx-GlyProHyp-$(N2picHypGly)_3$-$(GlyProHyp)_3$, respectively] were prepared by reacting the N-terminal amines of the sequences on-resin (1 eq) with 0.5 eq of sulfo-Cyanine5 NHS ester (designated as Cy5, Lumiprobe, 23320) and 3 eq of DIEA in DMSO for over 24 hours. The labeled peptides were cleaved from the resin, purified by HPLC, and analyzed by MALDI (Cy5-Pro-CMP, MALDI-MS, calculated 2625.50 $[M+H]^+$, observed: 2625.17 $[M+H]^+$; Cy5-N2pic3-CMP, MALDI-MS, calculated 2778.19 $[M+H]^+$, observed: 2777.46 $[M+H]^+$; Cy5-N2pic3-sCMP, MALDI-MS, calculated 2778.19

$[M+H]^+$, observed: 2779.68 $[M+H]^+$). The pure fluorescent peptides were lyophilized and were diluted to 50 µM in 1×PBS or 1 mM HCl solution as the stock solutions. Concentrations of Cy5-labeled peptide solution were determined by measuring the ultraviolet (UV) absorbance of the solutions at 646 nm (extinction coefficient: 271000 M^{-1} cm^{-1}).

Wells of a 96-well plate were coated with approximately 6 μL of a solution of 85°C gelatin (Sigma-Aldrich, V900863-100G) in 1× PBS (10% w/v) and incubated at 4°C for 15 min to gelatinize. The thin gelatin hydrogel films were crosslinked with an MES buffered solution (pH 4.7, 100 μL per well) containing 2 mM of NHS (N-hydroxysuccinimide) and 10 mM EDC [1-ethyl-3-(3-dimethylaminopropyl)carbodiimide] with shaking overnight at room temperature. The cross-linked gelatin films were washed with 200 µL 1×PBS solution (10 min×3). In the heating group, 1×PBS solutions (50 µL) containing 10 µM of Cy5-Pro-CMP were heated at 85 °C for 5 min to dissociate the triple-helices before being immediately cooled in an ice-water bath for 15 s and added to each well. In the unheated groups, probe stock solutions were diluted to 10 µM in 1×PBS solution and added to the wells immediately (50 µL). The cross-linked gelatin films were allowed to bind for overnight at 4 °C. Afterwards, the gelatin films were washed with 1×PBS at room temperature three times before their fluorescence was measured (ex: 646 nm, em: 662 nm).

In vivo skeleton targeting

All animal studies were approved by the experimental animal use and ethics committee of the Fifth Affiliated Hospital of Sun Yat-sen University and undertaken in compliance with the regulations (Project License: 00299). All mice were purchased from the Guangdong Medical Laboratory Animal Center. Each normal female BALB/c nude mouse (8–12 weeks old) was injected with 1 nmol Cy5-N2pic3-CMP or Cy5-N2pic3-sCMP in 100 μL PBS solution with or without preheating. In the heating group, 10 µM Cy5-N2pic3-CMP in 100 μL PBS solution was heated at 85 °C for 5 min and cooled in an ice-water bath for 15 s immediately before injection. In the other group, the peptide stock solutions were diluted to 10 µM in 100 μL PBS solution and were injected directly without heating. After 90 min, the mice were fluorescence imaged with an IVIS Spectrum imager (ex: 620 nm, em: 670 nm, PerkinElmer Lumina III). Then, the mice were sacrificed and their skins were removed to allow imaging of deep tissues. Major organs including hearts, lungs, kidneys, livers, and spleens were also harvested and imaged. The similar results obtained from three independent experiments.

The myocardial infarction (MI) model and in vivo targeting

The C57BL/6J mice (male, 8 weeks old) underwent surgical myocardial infarction or sham surgery.[18,19] Briefly, following pretreatment with butorphanol analgesia (2 mg kg^{-1}, sc), mice were anesthetized with isoflurane (induction at 3% isoflurane, 3 L min^{-1} oxygen, maintenance 1.5%), intubated, and mechanically ventilated

(respiratory frequency: 120 breaths per min, inspiratory to expiratory time ratio: 1:1, tidal volume: 0.5 mL). The heart was exposed by thoracotomy through the fourth intercostal space, and the left anterior descending artery (LAD artery) was permanently ligated with an 8-0 nylon suture. The myocardial area below the suture became pale after ligation in a few seconds. Mice in the sham group underwent the same surgical procedures as the MI group except for LAD ligation. The surgical wound was closed and mice recovered in a separate cage after being awake in an insulated and oxygenated environment.

Mice were used for in vivo tests at 7-14 days after surgical ligation of the left coronary artery. Cy5-N2pic3-CMP and Cy5-N2pic3-sCMP in 1 mM HCl solutions (~80 μL) were directly injected into the tail vein of mice with a dose of 4 nmol without preheating. After one hour, the mice were sacrificed and were transcardiacally perfused with 0.02% heparin in 1×PBS (m/V) and 1×PBS solution to remove blood. Perfused hearts were harvested and imaged in Z field of view on an IVIS Spectrum imager (ex: 620 nm, em: 670 nm, PerkinElmer Lumina III). The similar results obtained from three independent experiments.

Cryosections and immunofluorescence staining

Hearts of MI mice were harvested immediately after imaging and cryosectioned to 10 μm thickness on charged glass slides without any chemical fixation. The slides (with the in vivo administered Cy5-N2pic3-CMP bound) were incubated in PBS for 5 min to remove the OCT and were stained with an anti-collagen I antibody (Abcam ab34710; 1:300) overnight at 4 °C, followed by staining with a Cy3-labeled secondary antibody (goat anti-rabbit IgG H&L, Jackson, ab159083, diluted to 5 μg mL^{-1} in PBS) for 1 hour at room temperature. The slides were washed and sealed in an antifade mounting medium (Vector, H-1000). The slides were also stained with H&E (Phygene, PH0516).

All tissue sections were imaged using an EVOS M7000 imaging system (Thermo Fisher) with 10×, 20×, and 40× objective lenses, RFP, and Cy5 light cubes. The slides stained with H&E were imaged in a bright field. Large full-section images were generated through view-to-view scanning and image stitching performed automatically by the EVOS imaging system. Pseudo colors were assigned to the images using the LUT color scheme available in the ImageJ software.

Tissue clearing and light sheet fluorescence microscopy

One hour after intravenous injection of Cy5-N2pic3-CMP, the MI mice were sacrificed and were transcardiacally perfused with 0.02% heparin in 1×PBS (m/v) and 1×PBS solution to remove blood. The heart samples were cleared following the PEGASOS method.[20,21] First, heart specimens were fixed overnight at room temperature with 4% PFA (10 mL). Then the samples were washed with 1×PBS (10 min×3) and decolorized with 25% Quadrol aqueous solution (Sigma-Aldrich, 122262) for 48 hours. Then the samples were delipidated with *t*-BuOH (Sigma-Aldrich, 360538) and dehydrated with 70% v/v *t*-BuOH, 27% v/v PEGMEMA500 (Sigma-Aldrich, 447943), and 3% w/v

Quadrol for 48 hours. Finally, the samples were immersed in a clearing medium (BB-PEG) made by 75% v/v benzyl benzoate (Sigma-Aldrich, W213802), 22% v/v PEGMEMA500, and 3% w/v Quadrol for at least 48 hours. Care was taken to avoid light exposure during the entire clearing process.

Cleared mouse heart specimens were imaged on a LaVision Biotec Ultramicroscope II equipped with an sCMOS camera. The specimen was immersed in the imaging chamber filled with the BB-PEG medium. When imaged at a magnification of 4, each sample was scanned on both sides, and each side of the light sheet was composed of three beams with a step of 5 μm in the Z-axis. The images were acquired by continuous light sheet scanning, and the blend algorithm was used to stitch the images on both sides. Images were acquired by ImSpector (LaVision BioTec), saved as 16-bit grayscale TIFF images for each channel, and reconstructed with the Imaris software. Movies were produced at a frame rate of 25 fps.

Section 3: Synthesis and Characterization of Ac-X-OMe Model Compounds

All chemicals and solvents were of analytical grade. Reactions were monitored by thin-layer chromatography (TLC) using precoated silica gel plates (silica gel GF/UV 254), and spots were visualized under UV light (254 nm) or iodine vapor. Column chromatography was performed with silica gel (100-200 mesh). The purity of the compounds was verified by ^1H NMR and ^{13}C NMR spectra which were measured in CD_3OD on a Bruker AVANCE 400 MHz or 500 MHz at 25 °C. Chemical shifts (d) were expressed in parts per million relative to tetramethylsilane (TMS) which was used as an internal standard, and J values were given in Hz. The following multiplicity abbreviations were used: (s) singlet, (d) doublet, (t) triplet, (q) quartet, (m) multiplet, and (br) broad. ^1H NMR spectrum of Ac-X-OMe model compounds was recorded in CD_3OD to determine the $K_{cis/trans}$ ratios of the residue X. They were calculated by averaging the integration of two or three sets of related rotamer signals.[3,22-24] High-resolution mass spectra (HRMS) were recorded on a Thermo Q-Tof micro mass spectrometer. ^1H NMR spectra of Ac-Ala-OMe and Ac-Phe-OMe were acquired to verify that amino acids except proline favor the trans configuration overwhelmingly.

General procedure A: amine substitution

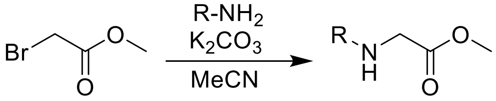

Bromoacetic acid (6.5 mmol, 1.00 eq) in MeCN (10.0 mL) was added dropwise into a solution of primary amine (16.3 mmol, 2.50 eq) and K_2CO_3 (1.80 g, 13.0 mmol, 2.00 eq) in MeCN (10.0 mL) at room temperature.[25] The reaction mixture was stirred for an additional 14 hours. The resulting mixture was filtered, and the filter cake was washed with MeCN (10.0 mL). The filtrate was concentrated under reduced pressure and was chromatographed over silica gel to give a pure secondary amine.

General procedure B: terminal acetylation

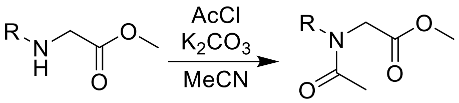

To a solution of secondary amine (6.5 mmol, 1.0 eq) and K_2CO_3 (1.80 g, 13 mmol, 2 eq) in MeCN (30.0 mL) was dropwise added AcCl (1.1 eq) in an ice bath. The reaction mixture was allowed to warm up to room temperature and was stirred for 6 hours. The resulting mixture was filtered, and the filter cake was washed with MeCN (10.0 mL). The solvent was removed under reduced pressure and added 30 mL water. The reaction mixture was extracted with EtOAc or 2% MeOH / DCM (15 mL×3), washed with brine, and dried over anhydrous sodium sulfate. The solvent was removed and the residue was chromatographed over silica gel to give a pure compound.

General procedure C: formation of TFA salt

Trifluoroacetic acid (44.1 μL,0.55 mmol, 1.05 eq) in CH_3OH (1.0 mL) was added dropwise into a solution of a peptoid (0.5 mmol, 1.00 eq) in CH_3OH (10.0 mL) in an ice bath. The solution was evaporated to yield TFA salt.

Ac-Ala-OMe

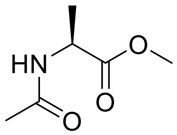

Ac-Ala-OMe was purchased from Macklin (A848899-250mg). HRMS (ESI): m/z calcd for $C_6H_{11}NO_3$ $[M+H]^+$ 146.0818, found 146.0812. ^{1}H NMR (400 MHz, CD_3OD) trans conformer δ 4.40 (q, J = 7.3 Hz, 1H), 3.73 (s, 3H), 1.98 (s, 3H), 1.38 (d, J = 7.3 Hz, 3H). ^{13}C NMR (101 MHz, CD_3OD) δ 173.40, 171.65, 51.33, 48.07, 20.89, 16.01.

Ac-Phe-OMe

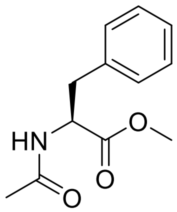

Ac-Phe-OMe was purchased from Energy Chemical (A023064-1g-A01). HRMS (ESI): m/z calcd for $C_{12}H_{15}NO_3$ $[M+H]^+$ 222.1131, found 222.1123. ^{1}H NMR (400 MHz, CD_3OD) trans conformer δ 7.30 (t, J = 7.2 Hz, 2H), 7.26 – 7.18 (m, 3H), 4.67 (dd, J = 8.8, 5.8 Hz, 1H), 3.70 (s, 3H), 3.15 (dd, J = 13.8, 5.7 Hz, 1H), 2.96 (dd, J = 13.8, 8.9 Hz, 1H), 1.92 (s, 3H). ^{13}C NMR (101 MHz, CD_3OD) δ 172.19, 171.73, 136.80, 128.79, 128.11, 126.51, 53.95, 51.28, 37.05, 20.88.

Ac-Pro-OMe

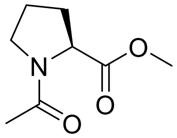

Ac-Pro-OMe was synthesized by application procedure B using methyl L-prolinate hydrochloride as the secondary amine. The crude product was purified by flash column chromatography (PE/EA = 2:1-1:2) to get a colorless oil (710 mg, 64%). HRMS (ESI): m/z calcd for $C_8H_{13}NO_3$ $[M+H]^+$ 172.0974, found 172.0970. ^{1}H NMR (500 MHz, CD_3OD) major conformer (trans) δ 4.41 (dd, J = 8.7, 4.0 Hz, 1H), 3.71 (s, 3H), 3.69 – 3.57 (m, 2H), 2.38 – 2.15 (m, 1H), 2.09 (s, 3H), 2.07 – 1.95 (m, 3H). Minor conformer (cis) δ 4.60 (dd, J = 8.7, 4.0 Hz, 1H), 3.77 (s, 3H), 3.47 –

3.56 (m, 2H), 2.38 – 2.15 (m, 1.14H), 2.07 – 1.95 (m, 3H), 1.97 (s, 3H). ^{13}C NMR (101 MHz, CD_3OD) δ 173.00, 172.64, 171.07, 170.71, 60.12, 58.69, 51.77, 51.33, 47.68, 46.09, 30.72, 29.07, 24.30, 22.29, 20.70.

Ac-Sar-OMe

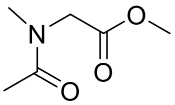

Ac-Sar-OMe was synthesized by application procedure B using sarcosine methyl ester hydrochloride as the secondary amine. The crude product was purified by flash column chromatography (DCM/MeOH = 200:1-50:1) to get a colorless oil (500 mg, 53%). HRMS (ESI): m/z calcd for $C_6H_{11}NO_3$ $[M+H]^+$ 146.0818, found 146.0812. ^{1}H NMR (500 MHz, CD_3OD) major conformer (trans) δ 4.14 (s, 2H), 3.74 (s, 3H), 3.13 (s, 3H), 2.15 (s, 3H). Minor conformer (cis) δ 4.24 (s, 2H), 3.79 (s, 3H), 2.95 (s, 3H), 2.05 (s, 3H). ^{13}C NMR (151 MHz, CD_3OD) δ 172.78, 172.74, 170.01, 169.90, 51.60, 51.51, 51.21, 48.81, 36.46, 33.65, 19.88, 19.75.

Ac-Nleu-OMe

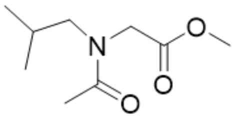

Ac-Nleu-OMe was synthesized by application of the general procedures: procedure A using isobutylamine as the primary amine and procedure B. The crude product was purified by flash column chromatography (PE/EA = 10:1-2:1) to get a colorless oil (650 mg, 53%). HRMS (ESI): m/z calcd for $C_9H_{17}NO_3$ $[M+H]^+$ 188.1287, found 188.1281. ^{1}H NMR (500 MHz, CD_3OD) major conformer (trans) δ 4.07 (s, 2H), 3.73 (s, 3H), 3.25 (d, J = 7.5 Hz, 2H), 2.16 (s, 3H), 1.99 – 1.92 (m, 1H), 0.98 (d, J = 6.6 Hz, 6H). Minor conformer (cis) δ 4.23 (s, 2H), 3.79 (s, 3H), 3.21 (d, J = 7.5 Hz, 2H), 2.05 (s, 3H), 1.92 – 1.84 (m, 1H), 0.90 (d, J = 6.6 Hz, 6H). ^{13}C NMR (151 MHz, CD_3OD) δ 172.97, 172.67, 170.17, 169.99, 57.19, 54.18, 51.52, 51.14, 50.33, 47.85, 27.57, 26.74, 20.17, 19.92, 19.06, 18.85.

Ac-Nphe-OMe

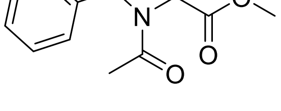

Ac-Nphe-OMe was synthesized by application of the general procedures: procedure A using benzylamine as the primary amine and procedure B. The crude product was purified by flash column chromatography (PE/EA = 10:1-2:1) to get a colorless oil (630 mg, 44%). HRMS (ESI): m/z calcd for $C_{12}H_{15}NO_3$ $[M+H]^+$ 222.1131, found 222.1123. ^{1}H NMR (500 MHz, CD_3OD) major conformer (trans) δ 7.42 – 7.25 (m, 5H), 4.71 (s, 2H), 4.07 (s, 2H), 3.71 (s,

3H), 2.21 (s, 3H). Minor conformer (cis) δ 7.42 – 7.25 (m, 5H), 4.62 (s, 2H), 4.14 (s, 2H), 3.70 (s, 3H), 2.14 (s, 3H). ^{13}C NMR (151 MHz, CD_3OD) δ 172.84, 172.77, 169.84, 136.63, 136.31, 128.64, 128.29, 127.97, 127.52, 127.30, 126.74, 52.94, 51.50, 51.23, 49.44, 49.02, 47.22, 20.11, 19.99.

Ac-Nchx-OMe

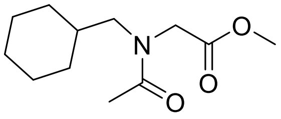

Ac-Nchx-OMe was synthesized by application of the general procedures: procedure A using cyclohexanemethanamine as the primary amine and procedure B. The crude product was purified by flash column chromatography (PE/EA = 10:1-2:1) to get a colorless oil (590mg, 40%). HRMS (ESI): m/z calcd for $C_{12}H_{21}NO_3$ $[M+H]^+$ 228.1600, found 228.1595. ^{1}H NMR (500 MHz, CD_3OD) major conformer (trans) δ 4.06 (s, 2H), 3.72 (s, 3H), 3.27 (d, J = 7.3 Hz, 2H), 2.15 (s, 3H), 1.85 – 1.53 (m, 6H), 1.37 – 1.16 (m, 3H), 1.00 (m, 2H). Minor conformer (cis) δ 4.22 (s, 2H), 3.78 (s, 3H), 3.23 (d, J = 7.4 Hz, 2H), 2.15 (s, 3H), 1.85 – 1.53 (m, 6H), 1.37 – 1.16 (m, 3H), 1.00 (dd, J = 11.8, 3.6 Hz, 2H). ^{13}C NMR (151 MHz, CD_3OD) δ 172.89, 172.64, 170.17, 170.00, 56.21, 53.07, 51.52, 51.14, 50.49, 48.03, 37.15, 36.25, 30.45, 26.18, 26.10, 25.64, 20.16, 19.92.

Ac-Nasn-OMe

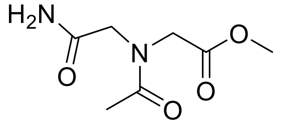

Ac-Nasn-OMe was synthesized by application of the general procedures: procedure A using glycinamide hydrochloride as the primary amine and procedure B. The crude product was purified by flash column chromatography (DCM/MeOH = 100:1-10:1) to get a colorless oil (200 mg, 17%). HRMS (ESI): m/z calcd for $C_7H_{12}N_2O_4$ $[M+H]^+$ 189.0876, found 189.0870. ^{1}H NMR (500 MHz, CD_3OD) major conformer (trans) δ 4.17 (s, 2H), 4.17 (s, 2H), 3.76 (s, 3H), 2.12 (s, 3H). Minor conformer (cis) δ 4.32 (s, 2H), 4.05 (s, 2H), 3.80 (s, 3H), 2.10 (s, 3H). ^{13}C NMR (151 MHz, CD_3OD) δ 173.22, 173.20, 172.49, 172.02, 170.75, 170.43, 52.48, 52.45, 51.71, 51.44, 51.04, 49.83, 49.01, 19.74, 19.69.

Ac-Nlys-OMe·TFA

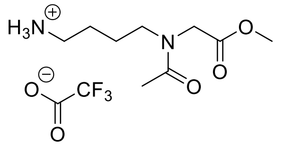

Boc-protected Ac-Nlys-OMe was synthesized by application of the general procedures: procedure A using N-Boc-1,4-butanediamine as the primary amine and procedure B. The Boc-protected peptoid was purified by flash column chromatography (PE/EA = 10:1-1:1) to get a colorless oil (630 mg, 32%). Boc-protected peptoid in 50% TFA/DCM (10 mL) was stirred for 30 min at room temperature. The mixture was evaporated to yield a pale-yellow oil (659 mg, 99%). HRMS (ESI): m/z calcd for $C_9H_{18}N_2O_3$ $[M+H]^+$ 203.1396, found 203.1392. ^{1}H NMR (500 MHz, CD_3OD) major conformer (trans) δ 4.10 (s, 2H), 3.74 (s, 3H), 3.52 – 3.46 (m, 2H), 3.03 – 2.93 (m, 2H), 2.18 (s, 3H), 1.76 – 1.69 (m, 2H), 1.69 – 1.64 (m, 2H). Minor conformer (cis) δ 4.27 (s, 2H), 3.80 (s, 3H), 3.44 (t, J = 6.9 Hz, 2H), 3.03 – 2.93 (m, 2H), 2.05 (s, 3H), 1.76 – 1.69 (m, 2H), 1.64 – 1.58 (m, 2H). ^{13}C NMR (151 MHz, CD_3OD) δ 173.00, 172.41, 170.38, 170.25, 51.67, 51.65, 51.30, 51.28, 50.02, 49.47, 46.23, 39.14, 39.05, 25.20, 24.34, 24.30, 24.03, 20.20, 19.80.

Ac-N2pic-OMe

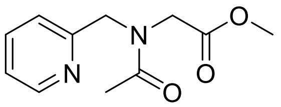

Ac-N2pic-OMe was synthesized by application of the general procedures: procedure A using 2-picolylamine as the primary amine and procedure B. The crude product was purified by the flash column chromatography (DCM/MeOH = 120:1-40:1) to get a colorless oil (721 mg, 50%).^{1}H NMR (400 MHz, CD_3OD) major conformer (trans) δ 8.50 (dddd, J = 39.2, 4.9, 1.8, 0.9 Hz, 1H), 7.91 – 7.74 (m, 1H), 7.48 – 7.26 (m, 2H), 4.78 (s, 2H), 4.14 (s, 2H), 3.70 (s, 2H), 2.19 (s, 2H). Minor conformer (cis) δ 8.50 (dddd, J = 39.2, 4.9, 1.8, 0.9 Hz, 1H), 7.91 – 7.74 (m, 1H), 7.48 – 7.26 (m, 2H), 4.69 (s, 2H), 4.30 (s, 2H), 3.73 (s, 2H), 2.12 (s, 3H).

Ac-N2pic-OMe·TFA

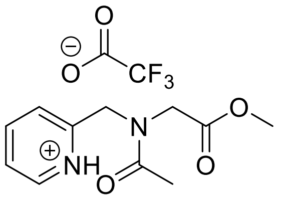

Ac-N2pic-OMe·TFA was synthesized by application procedure C using Ac-N2pic-OMe as the material and obtained a colorless oil (168 mg, 100%). HRMS (ESI): m/z calcd for $C_{11}H_{14}N_2O_3$ $[M+H]^+$ 223.1083, found 223.1076. ^{1}H NMR (400 MHz, CD_3OD) major conformer (cis) δ 8.82 – 8.35 (m, 2H), 8.14 – 7.78 (m, 2H), 4.91 (s, 2H), 4.51 (s, 2H), 3.81 (s, 3H), 2.16 (s, 3H). Minor conformer (trans) δ 8.82 – 8.35 (m, 2H), 8.14 – 7.78 (m, 2H), 5.07 (s, 2H), 4.21 (s, 2H), 3.74 (s, 3H), 2.21 (s, 3H). ^{13}C NMR (101 MHz, CD_3OD) δ 173.94, 170.31, 153.47, 146.19, 141.07, 125.89, 125.35, 51.80, 51.26, 49.33, 19.76.

Ac-Nme$_2$ae-OMe

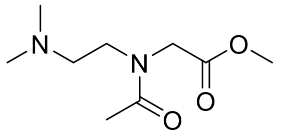

Ac-Nme$_2$ae-OMe was synthesized by application of the general procedures: procedure A using N,N-dimethyl ethylenediamine as the primary amine and procedure B. The crude product was purified by the flash column chromatography (DCM/MeOH = 100:1-10:1) to get a colorless oil (260 mg, 20%).^{1}H NMR (400 MHz, CD_3OD) major conformer (trans) δ 4.13 (s, 2H), 3.73 (s, 2H), 3.59 – 3.50 (m, 2H), 3.37 (s, 3H), 2.57 (m, 2H), 2.30 (s, 3H), 2.19 (s, 3H). Minor conformer (cis) δ 4.26 (s, 2H), 3.79 (s, 2H), 3.59 – 3.50 (m, 2H), 2.57 (m, 2H), 2.30 (s, 3H), 2.04 (s, 2H).

Ac-Nme$_2$ae-OMe·TFA

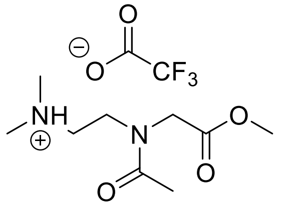

Ac-Nme$_2$ae-OMe·TFA was synthesized by application procedure C using Ac-Nme$_2$ae-OMe as the material and obtained a colorless oil (155 mg, 98%). HRMS (ESI): m/z calcd for $C_9H_{18}N_2O_3$ $[M+H]^+$ 203.1396, found 203.1389. ^{1}H NMR (400 MHz, CD_3OD) major conformer (cis) δ 4.34 (s, 2H), 3.82 (t, 3H), 3.82 (s, 2H), 3.36 – 3.34 (m, 2H), 2.98 (s, 6H), 2.07 (s, 3H). Minor conformer (trans) δ 4.15 (s, 2H), 3.88 (t, 3H), 3.76 (s,2H), 3.45 – 3.42 (m, 2H), 2.98 (s, 6H), 2.23 (s, 3H). ^{13}C NMR (101 MHz, CD_3OD) δ 174.21, 172.48, 170.91, 170.69, 55.94, 54.65, 51.88, 51.45, 50.35, 44.74, 42.85, 42.62, 42.45, 20.00, 19.61.

Ac-Net$_2$ae-OMe

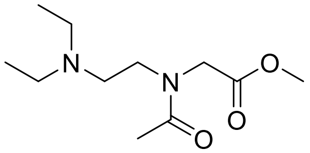

Ac-Net$_2$ae-OMe was synthesized by application of the general procedures: procedure A using N, N-diethyl ethylenediamine as the primary amine and procedure B. The crude product was purified by flash column chromatography (DCM/MeOH = 100:1-10:1) to get a colorless oil (330 mg, 22%). ^{1}H NMR (400 MHz, CD_3OD) major conformer (trans) δ 4.14 (s, 2H), 3.73 (m, 3H), 3.55 – 3.47 (m, 2H), 2.71 – 2.57 (m, 6H), 2.20 (s, 3H), 1.08

(dt, J = 8.8, 7.2 Hz, 7H). Minor conformer (cis) δ 4.29 (s, 2H), 3.79 (s, 3H), 3.55 – 3.47 (m, 2H), 2.71 – 2.57 (m, 6H), 2.04 (s, 3H), 1.08 (dt, J = 8.8, 7.2 Hz, 6H).

Ac-Net$_2$ae-OMe·TFA

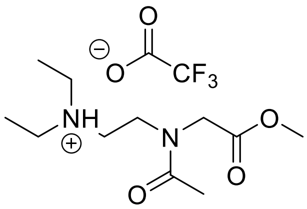

Ac-Net$_2$ae-OMe·TFA was synthesized by application procedure C using Ac-Net$_2$ae-OMe as the material and obtained a colorless oil (171 mg, 99%). HRMS (ESI): m/z calcd for $C_{11}H_{22}N_2O_3$ $[M+H]^+$ 231.1709, found 231.1700. ^{1}H NMR (400 MHz, CD_3OD) δ major conformer (cis) δ 4.37 (s, 2H), 3.82 (s, 3H), 3.78 (t, *J* = 6.3 Hz, 2H), 3.38 – 3.25 (m, 6H), 2.08 (s, 3H), 1.35 (t, *J* = 7.3 Hz, 6H). Minor conformer (trans) δ 4.17 (s, 2H), 3.90-3.86 (t, *J* = 6.0 Hz, 2H), 3.70 (s, 3H), 3.38 – 3.25 (m, 6H), 2.24 (s, 3H), 1.35 (t, J = 7.3 Hz, 6H). ^{13}C NMR (101 MHz, CD_3OD) δ 174.38, 172.48, 170.55, 170.44, 51.82, 51.41, 50.92, 50.04, 49.34, 44.62, 43.05, 20.05, 19.72, 7.77, 7.72.

Ac-N^{i}pr$_2$ae-OMe

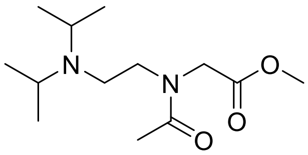

Ac-N^{i}pr$_2$ae-OMe was synthesized by application of the general procedures: procedure A using N, N-diisopropyl ethylenediamine as the primary amine and procedure B. The crude product was purified by flash column chromatography (DCM/MeOH = 100:1-10:1) to get a colorless oil (280 mg, 17%). ^{1}H NMR (400 MHz, CD_3OD) major conformer (trans) δ 4.16 (s, 2H), 3.74 (s, 3H), 3.44 – 3.35 (m, 2H), 3.06 (p, *J* = 6.6 Hz, 2H), 2.69 (dd, J = 7.4, 6.3 Hz, 2H), 2.21 (s, 3H), 1.05 (d, *J* = 6.6 Hz, 12H). Minor conformer (cis) δ 4.31 (s, 2H), 3.79 (s, 3H), 3.44 – 3.35 (m, 2H), 3.06 (p, J = 6.6 Hz, 2H), 2.65 – 2.59 (m, 2H), 2.04 (s, 3H), 1.05 (d, *J* = 6.6 Hz, 12H).

Ac-N^{i}pr$_2$ae-OMe·TFA

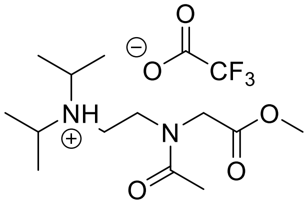

Ac-N^{i}pr$_2$ae-OMe·TFA was synthesized by application procedure C using Ac-N^{i}pr$_2$ae-OMe as the material and obtained a colorless oil (186 mg, 100%). HRMS (ESI): m/z calcd for $C_{13}H_{26}N_2O_3$ $[M+H]^+$ 259.2022, found 259.2010. ^{1}H NMR (400 MHz, CD_3OD) major conformer (cis) δ 4.41 (s, 2H), 3.83 (s, 3H), 3.84 – 3.80 (m, 4H), 3.40 (t, J = 6.4 Hz, 2H), 2.11 (s, 3H), 1.48 – 1.44 (m, 1H), 1.41 (dd, J = 6.6, 1.8 Hz, 12H). Minor conformer (trans): 4.19 (s, 2H), 3.76 (s, 3H), 3.77-3.74 (m, 4H), 3.42-3.39 (m, 2H), 2.26 (s, 3H), 1.44 (m, 12 H). ^{13}C NMR (101 MHz, CD_3OD) δ 175.14, 172.35, 171.51, 170.45, 55.38, 54.97, 51.86, 51.82, 51.45, 46.50, 46.32, 45.83, 44.42, 20.00, 19.81, 17.34, 16.19, 16.13.

Ac-Nbtm$^+$-OMe

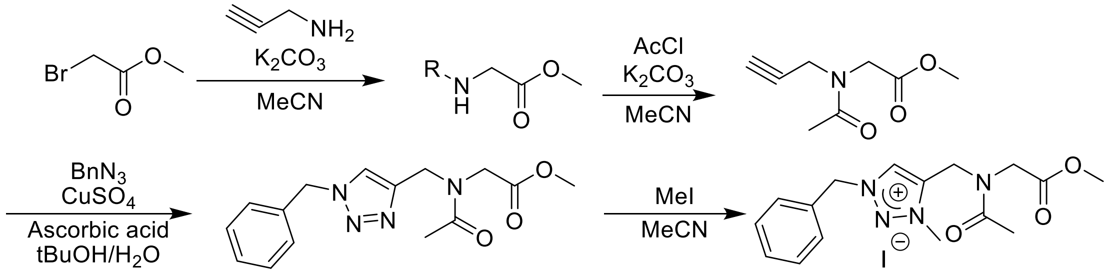

The intermediate Ac-Nakn-OMe was synthesized by application of the general procedures: procedure A using propargylamine as the primary amine and procedure B. The crude product was purified by flash column chromatography (PE/EA = 6:1-2:1) to get a colorless oil (600 mg, 55%). To a solution of Ac-Nakn-OMe (550 mg, 3.2 mmol, 1 equiv) in t-BuOH (10 mL) was added freshly prepared 0.1 M aq. ascorbic acid (0.24 equiv), 0.1 M aq. $CuSO_4$ (0.08 equiv) and the benzylazide (851mg, 6.4 mmol).[24] After stirring for 6 hours at room temperature under a nitrogen atmosphere, water (16 mL) was added and the product was extracted with CH_2Cl_2 (3×10 mL). The combined organic layer was washed with brine solution and dried with Na_2SO_4. The residue was concentrated and subjected to column chromatography on silica (PE/EA = 1:1-1:4) to obtain the triazole product (450 mg, 45%). To a solution of triazole (200 mg, 0.70 mmol, 1 equiv) in anhydrous MeCN (4 mL) was added MeI (0.87 mL, 14 mmol, 20 equiv). The resulting mixture was stirred at 70°C for 24 hours under a nitrogen atmosphere and then evaporated to yield the crude product. The Ac-Nbtm$^+$-OMe was purified by flash column chromatography (DCM/MeOH = 100:1-20:1) to get a solid (130 mg, 58%). HRMS (ESI): m/z calcd for $C_{16}H_{21}N_4O_3$ $[M]^+$ 317.1614, found 317.1606. ^{1}H NMR (400 MHz, CD_3OD) major conformer (cis) δ 7.59 – 7.37 (m, 6H), 5.83 (s, 2H), 4.84 (s, 2H), 4.33 (s, 3H), 3.37 (s, 3 H), 2.10 (s, 3H). Minor conformer (trans) δ 7.59 – 7.37 (m, 6H), 5.85 (s, 3H), 5.06 (s, 2H), 4.32 (s, 3H), 2.25 (s, 3H). ^{13}C NMR (101 MHz, CD_3OD) δ 173.30, 172.66, 170.13, 169.85, 141.51, 141.03, 132.15, 132.05, 129.78, 129.45, 129.10, 129.07, 129.00, 57.19, 56.97, 52.02, 51.57, 50.92, 48.45, 44.21, 40.14, 38.42, 38.08, 20.47, 20.16.

HRMS, ^{1}H NMR, and ^{13}C NMR spectra of Ac-X-OMe model compounds

Ac-Ala-OMe

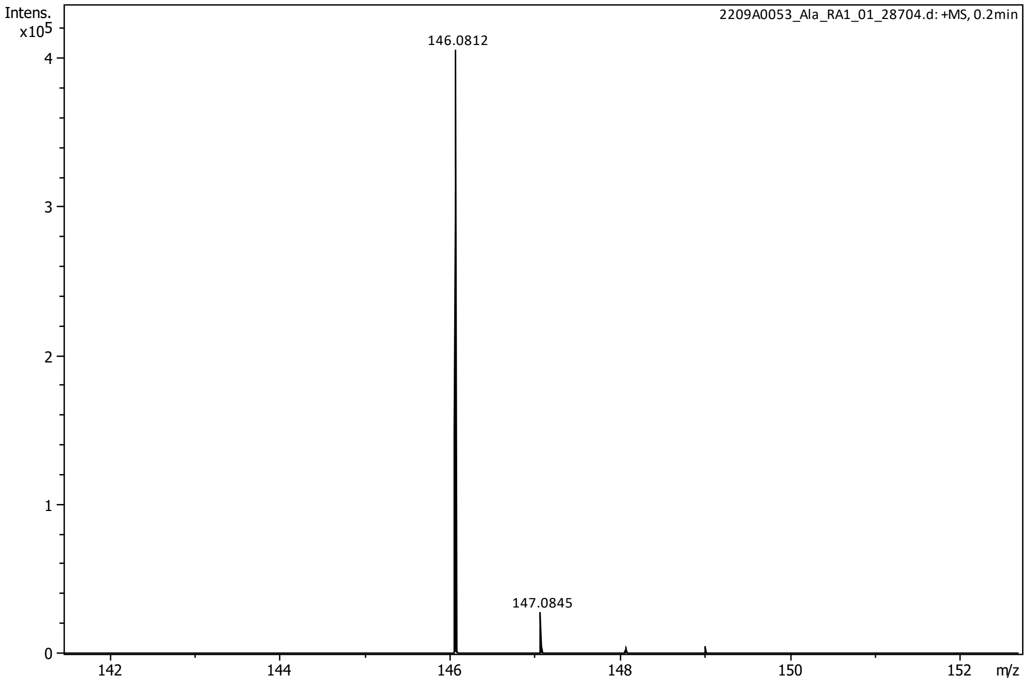

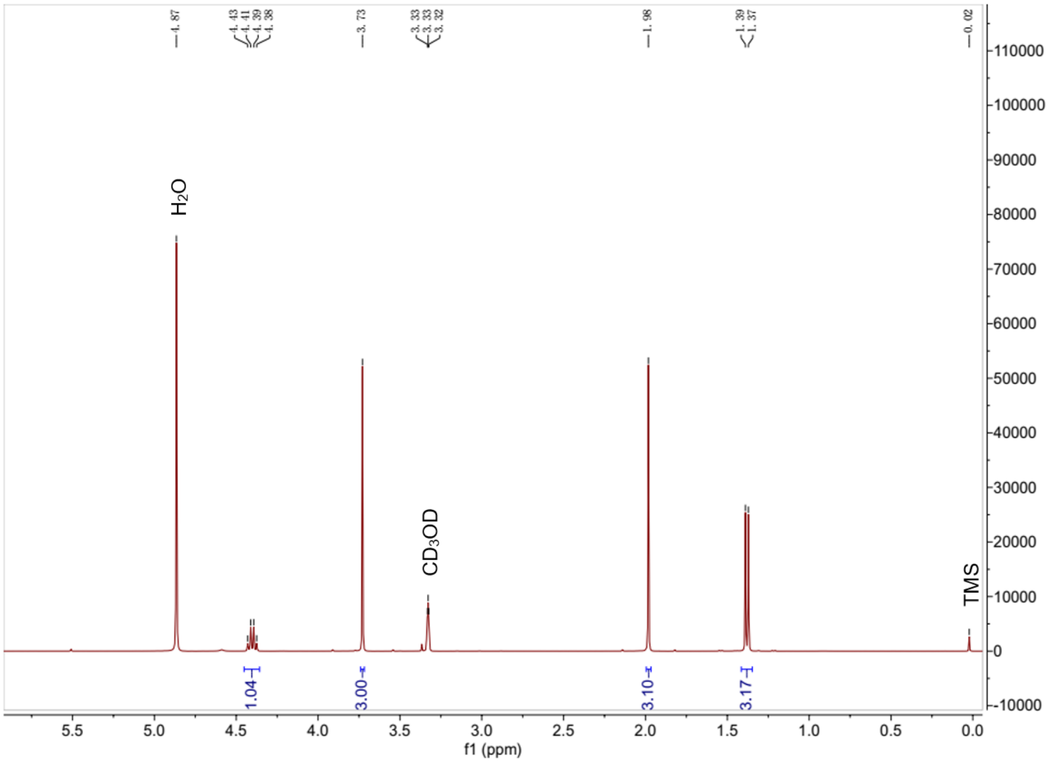

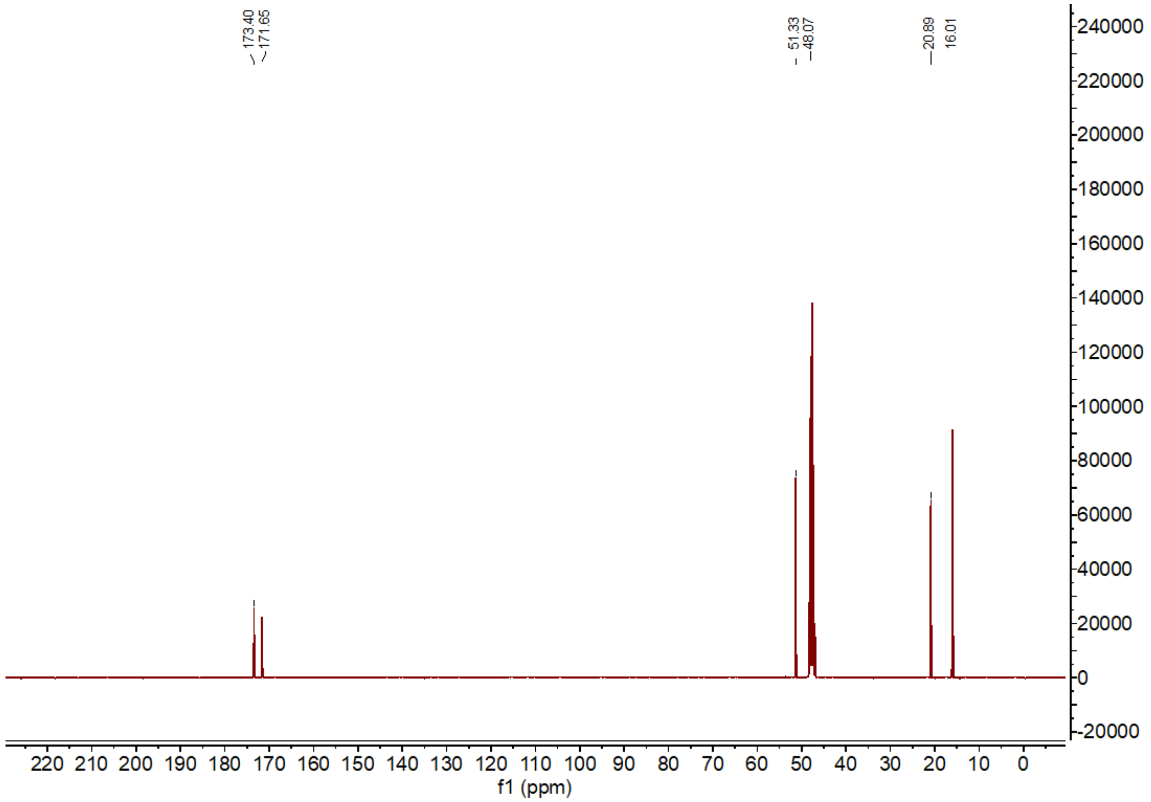

173.40
171.65
51.33
48.07
20.89
16.01
f1 (ppm)

Ac-Phe-OMe

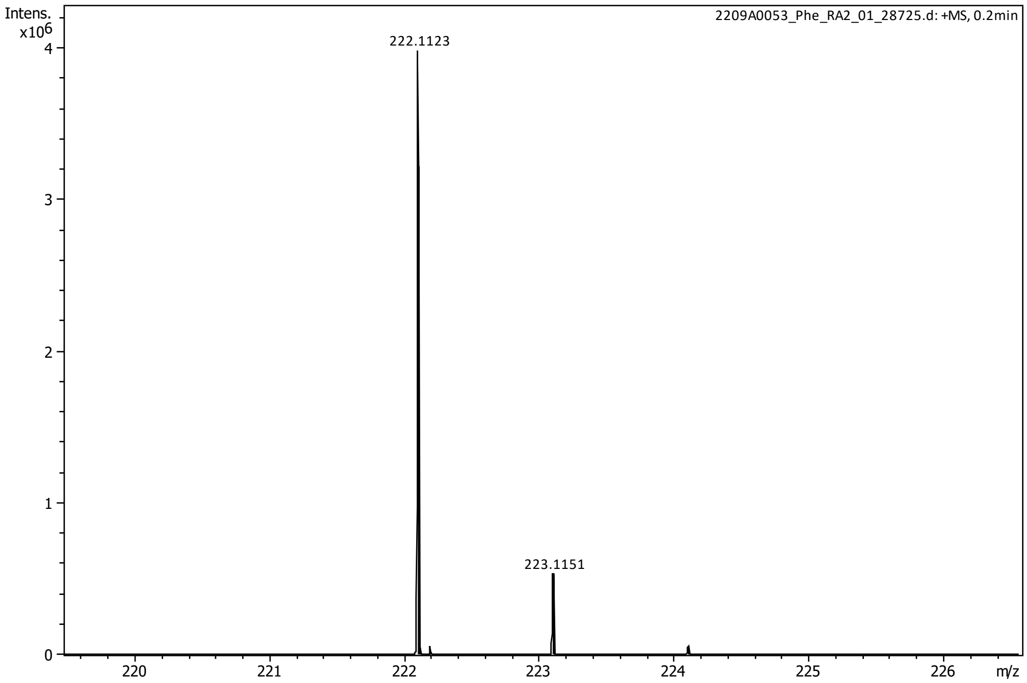

Intens.
x10^6
2209A0053_Phe_RA2_01_28725.d: +MS, 0.2min
222.1123
223.1151
m/z

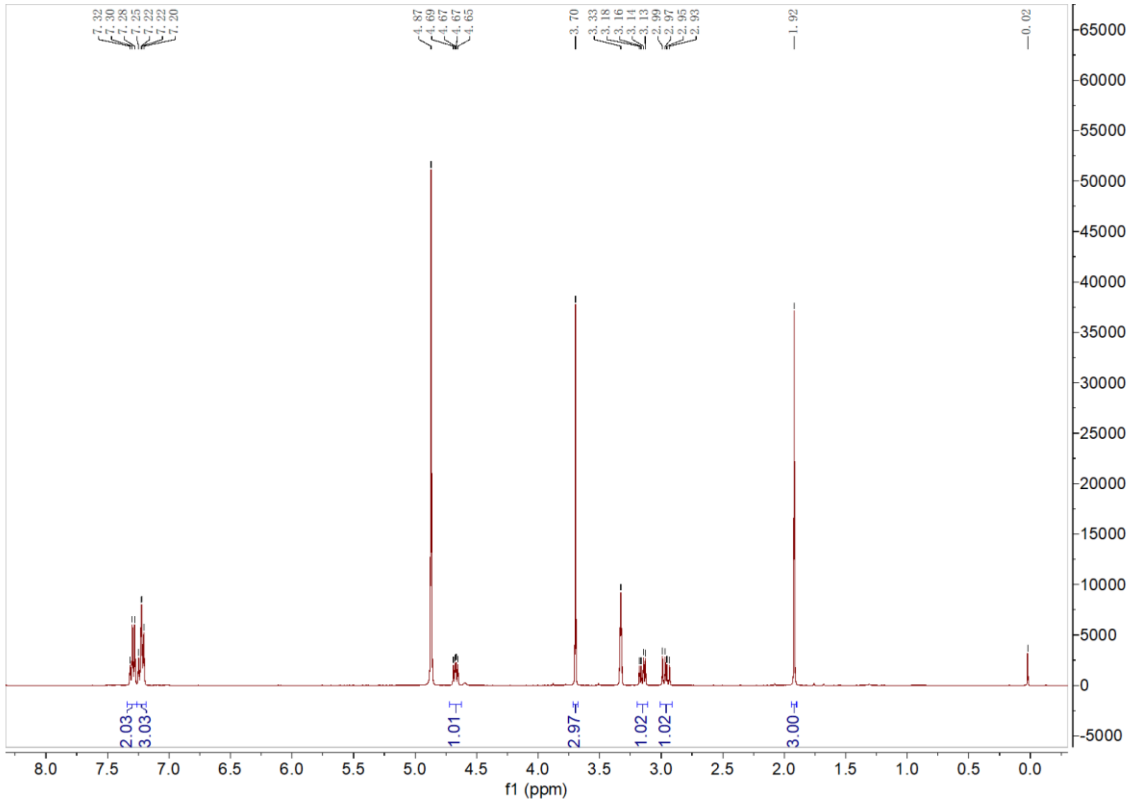

7.32
7.30
7.28
7.25
7.22
7.22
7.20
4.87
4.69
4.67
4.67
4.65
3.70
3.33
3.18
3.16
3.14
3.13
2.99
2.97
2.95
2.93
1.92
0.02
2.03
3.03
1.01
2.97
1.02
1.02
3.00
f1 (ppm)

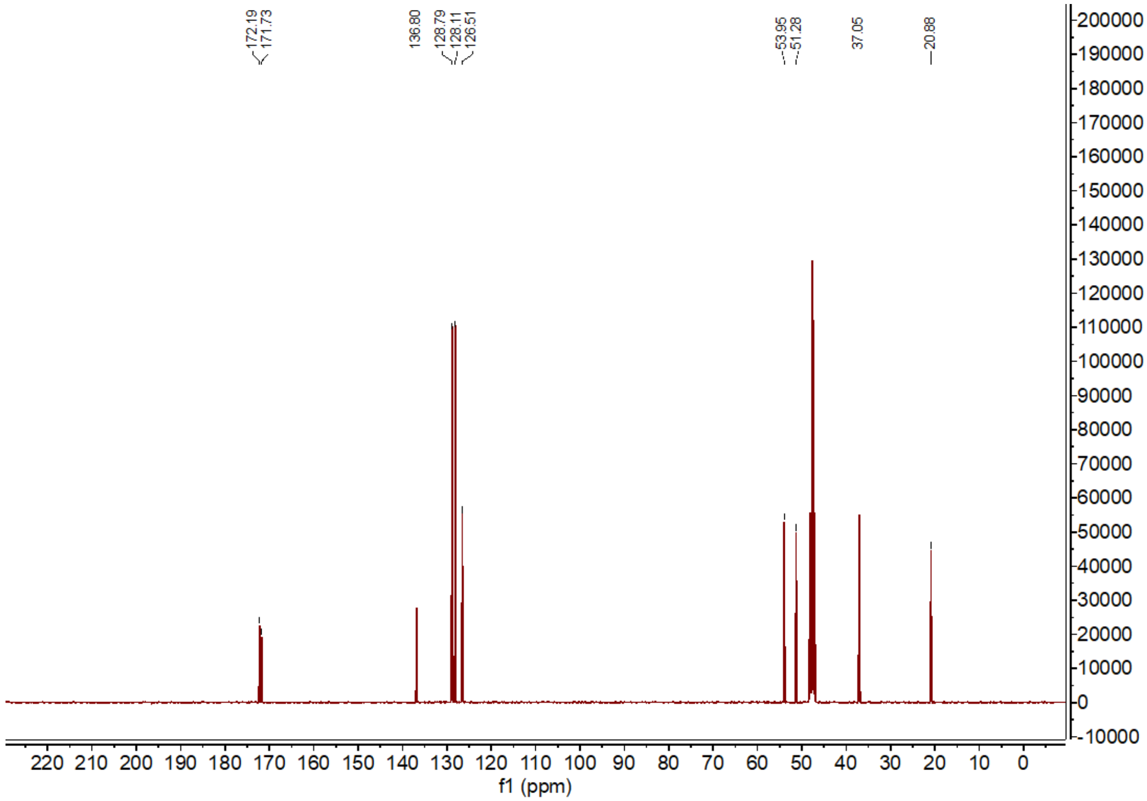

172.19
171.73
136.80
128.79
128.11
126.51
53.95
51.28
37.05
20.88
f1 (ppm)

Ac-Pro-OMe

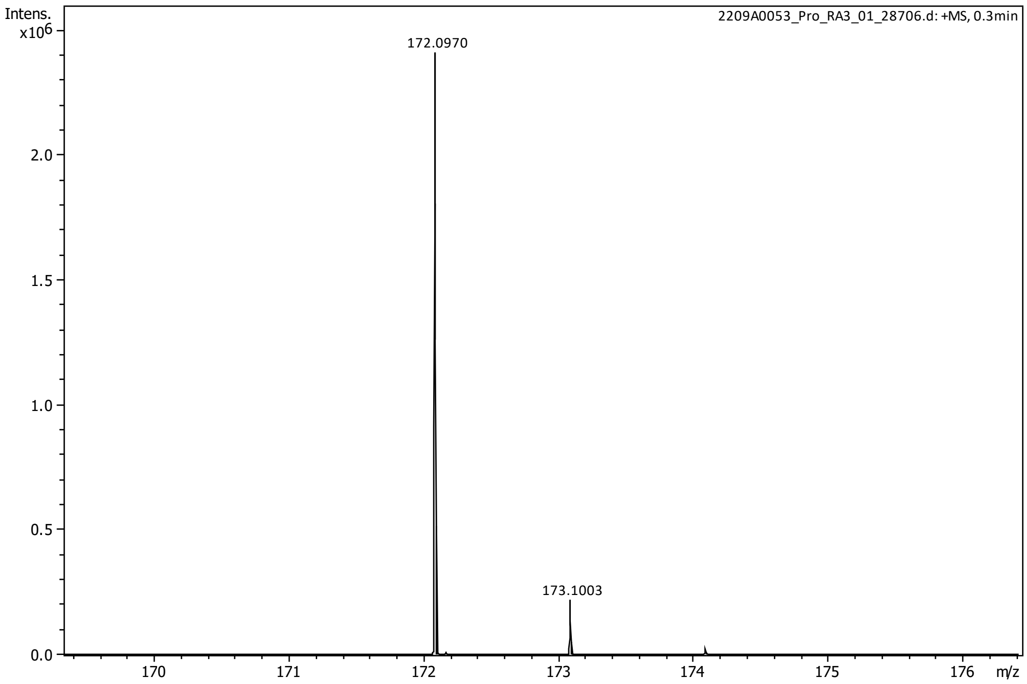

Intens.
x10^6
2209A0053_Pro_RA3_01_28706.d: +MS, 0.3min
172.0970
173.1003
2.0
1.5
1.0
0.5
0.0
170
171
172
173
174
175
176
m/z

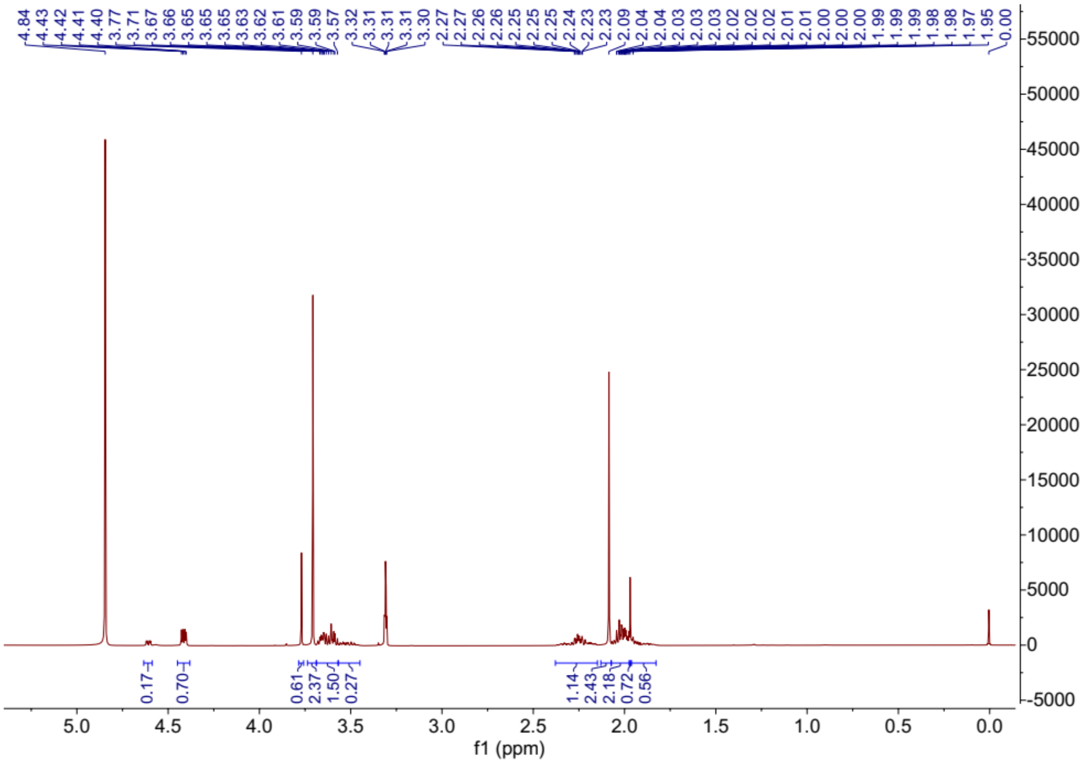

4.84
4.43
4.42
4.41
4.40
3.77
3.71
3.67
3.66
3.65
3.65
3.63
3.62
3.61
3.59
3.57
3.32
3.31
3.31
3.30
2.27
2.27
2.26
2.25
2.25
2.24
2.23
2.09
2.04
2.03
2.03
2.02
2.02
2.01
2.00
2.00
1.99
1.99
1.98
1.98
1.97
1.95
0.00
0.17
0.70
0.61
2.37
1.50
0.27
1.14
2.43
2.18
0.72
0.56
f1 (ppm)

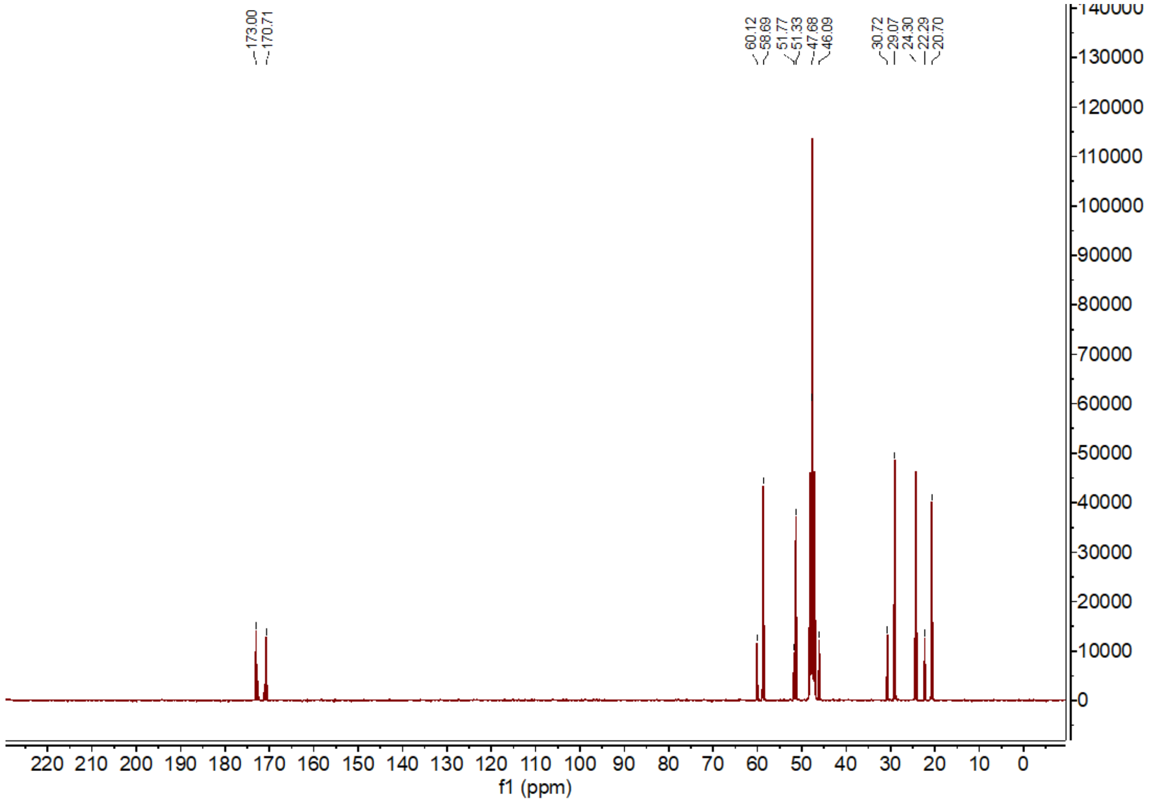
173.00
170.71
60.12
58.69
51.77
51.33
47.68
46.09
30.72
29.07
24.30
22.29
20.70
f1 (ppm)

Ac-Sar-OMe

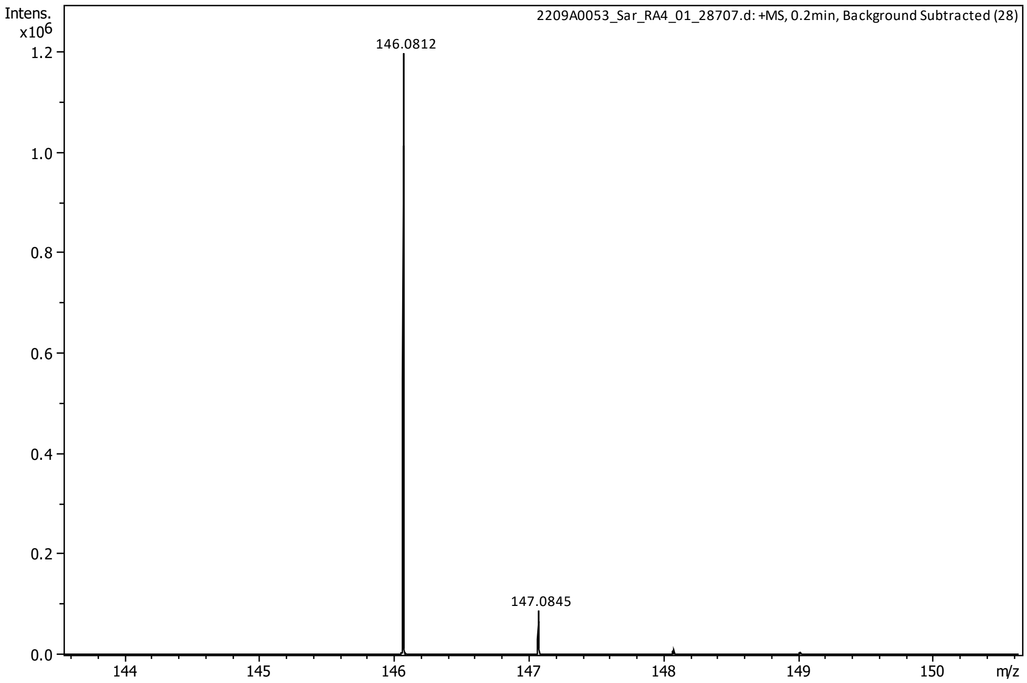
Intens.
x10^6
2209A0053_Sar_RA4_01_28707.d: +MS, 0.2min, Background Subtracted (28)
146.0812
147.0845
m/z

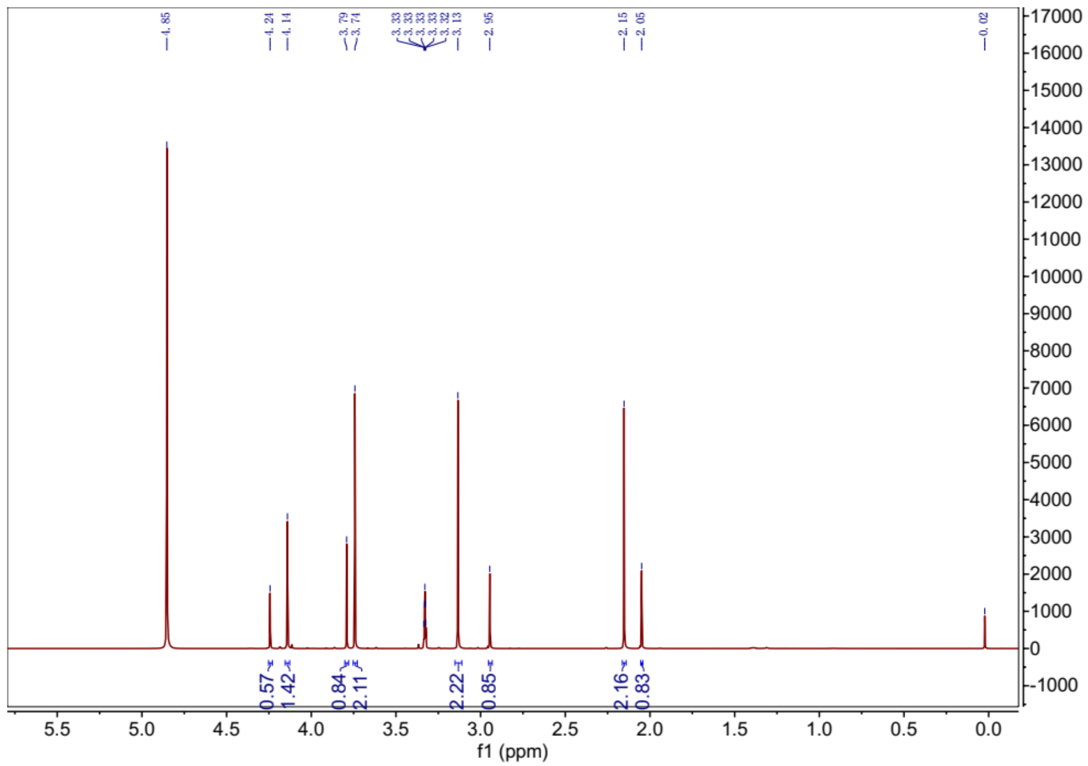
4.85
4.24
4.14
3.79
3.74
3.33
3.33
3.33
3.33
3.32
3.13
2.95
2.15
2.05
0.02
0.57
1.42
0.84
2.11
2.22
0.85
2.16
0.83
f1 (ppm)

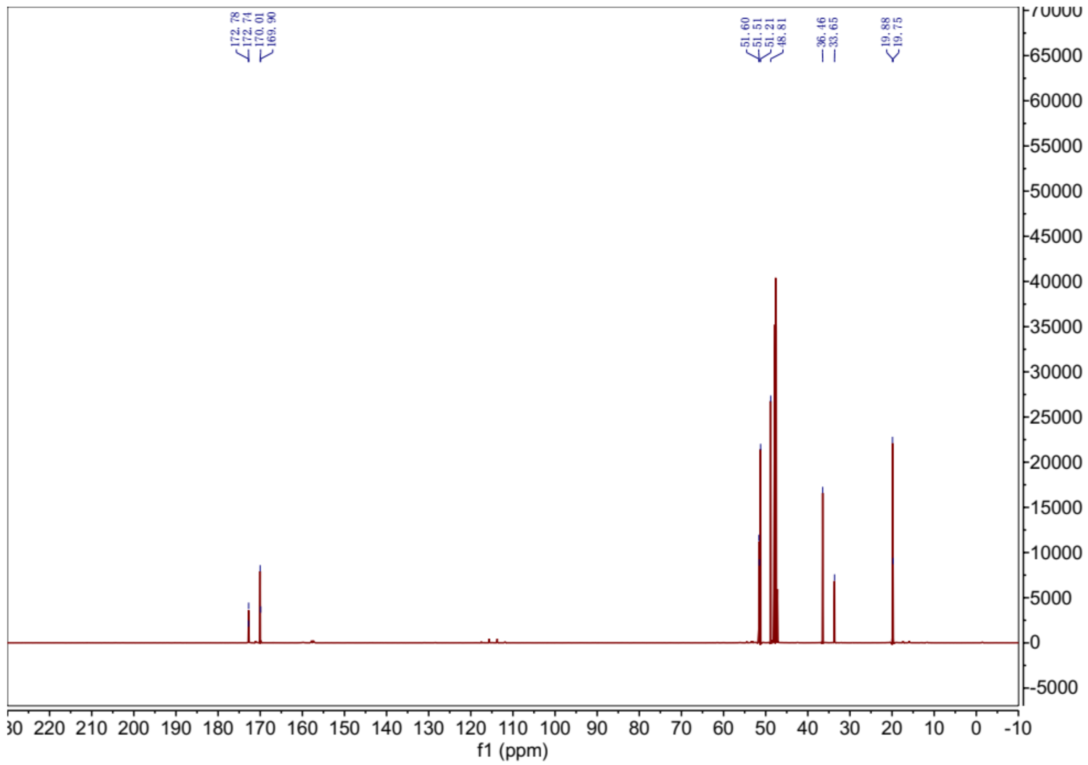
172.78
172.74
170.01
169.90
51.60
51.51
51.21
48.81
36.46
33.65
19.88
19.75
f1 (ppm)

Ac-Nleu-OMe

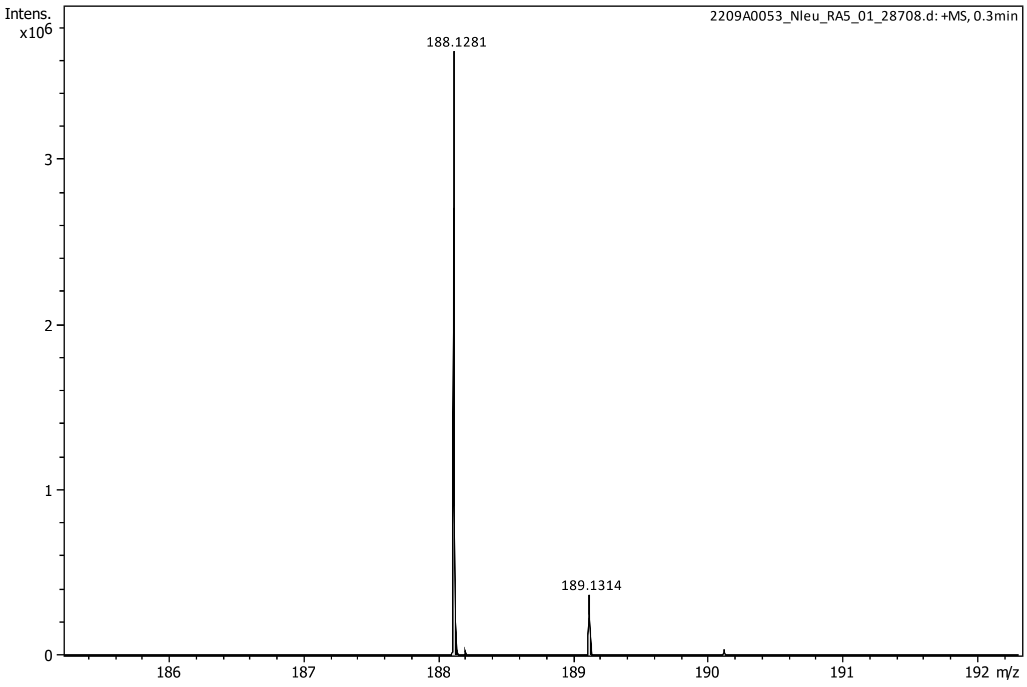

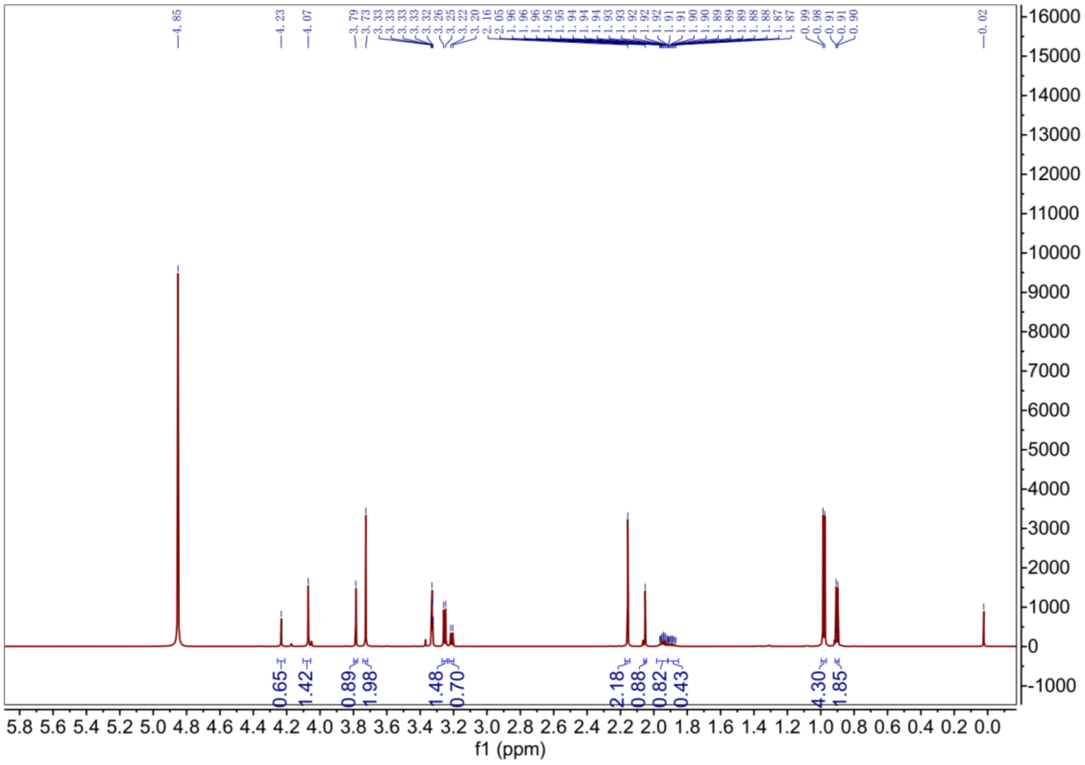

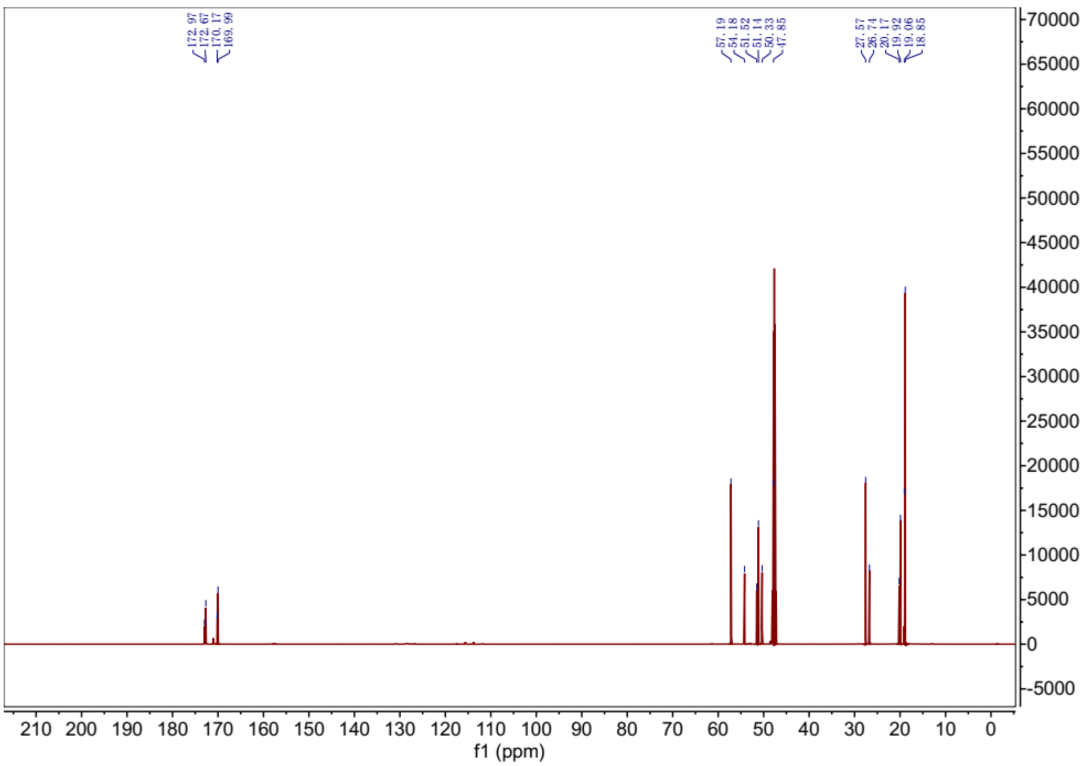

172.97
172.67
170.17
169.99
57.19
54.18
51.52
51.14
50.33
47.85
27.57
26.74
20.17
19.92
19.06
18.85
f1 (ppm)

Ac-Nphe-OMe

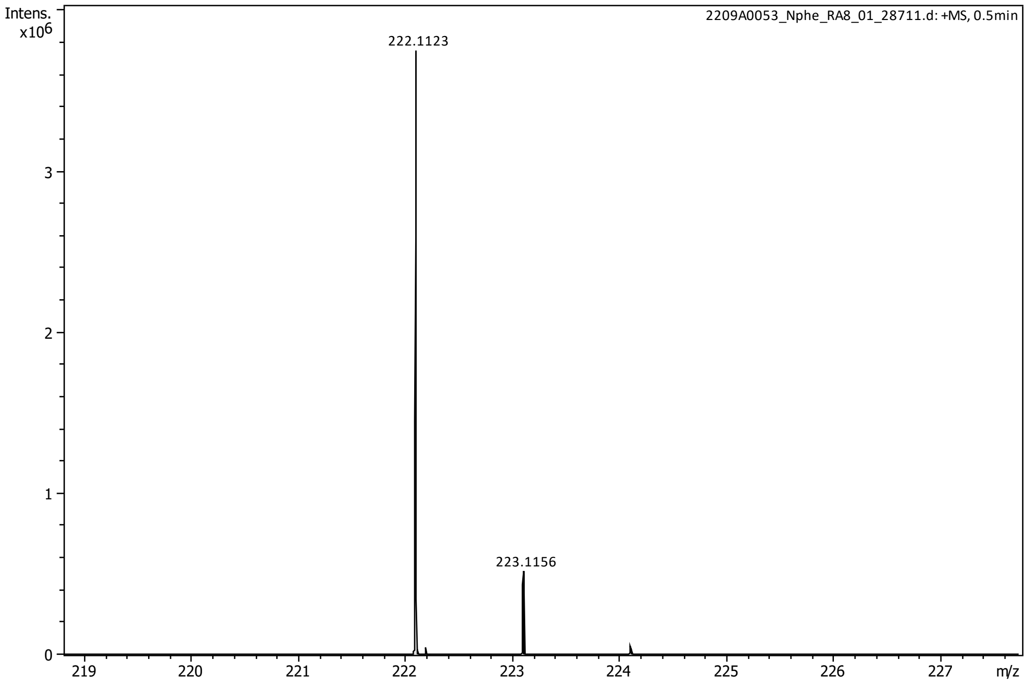

Intens.
x10^6
2209A0053_Nphe_RA8_01_28711.d: +MS, 0.5min
222.1123
223.1156
m/z

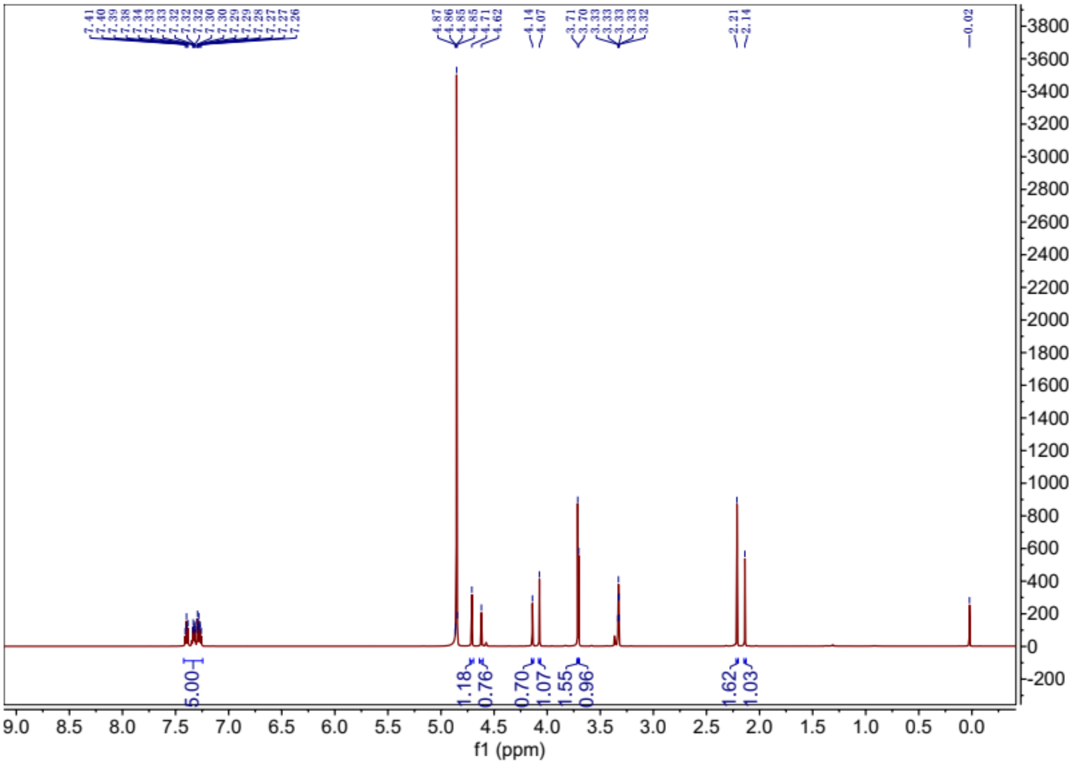
7.41 7.40 7.39 7.38 7.34 7.33 7.33 7.32 7.32 7.32 7.30 7.30 7.29 7.29 7.28 7.27 7.26
4.87 4.86 4.85 4.71 4.62
4.14 4.07
3.71 3.70 3.33 3.33 3.33 3.33 3.32
2.21 2.14
0.02
5.00
1.18 0.76 0.70 1.07 1.55 0.96
1.62 1.03
f1 (ppm)

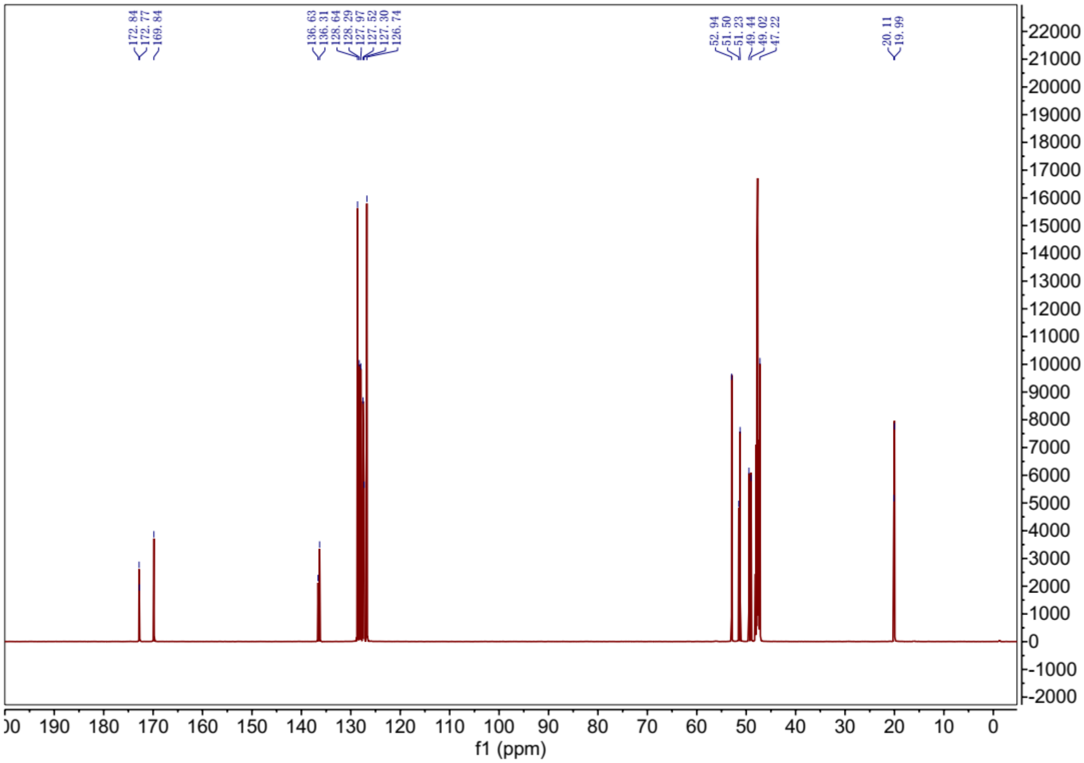
172.84 172.77 169.84
136.63 136.31 128.64 128.29 127.97 127.52 127.30 126.74
52.94 51.50 51.23 49.44 49.02 47.22
20.11 19.99
f1 (ppm)

Ac-Nchx-OMe

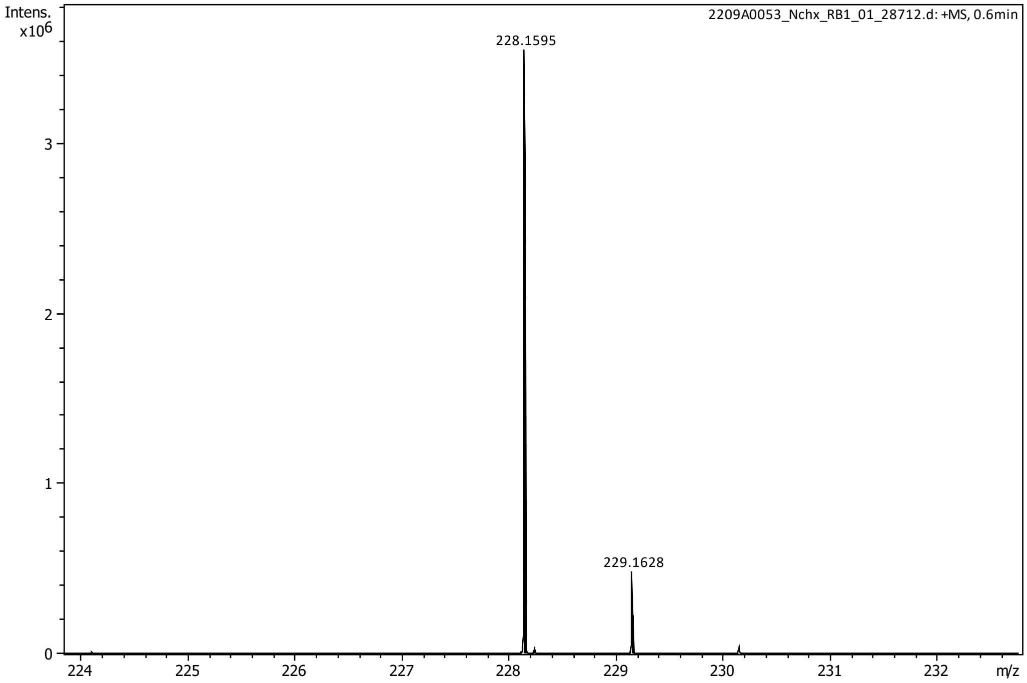
Intens.
x10^6
2209A0053_Nchx_RB1_01_28712.d: +MS, 0.6min
228.1595
229.1628
m/z

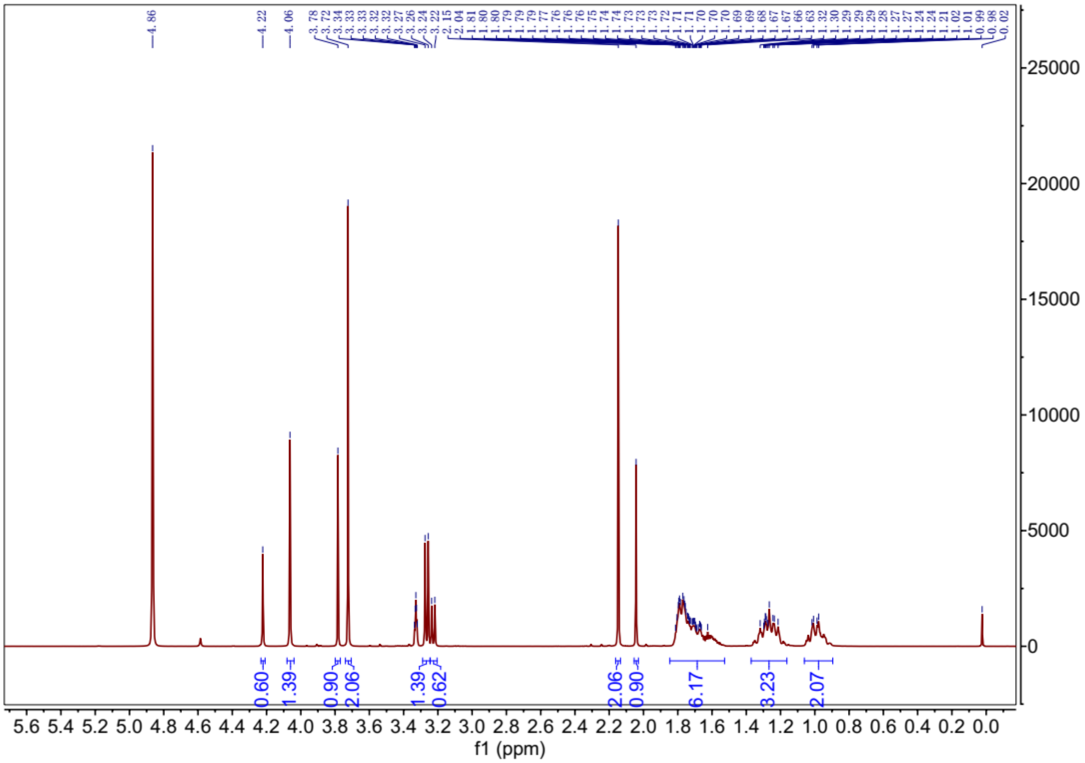
f1 (ppm)

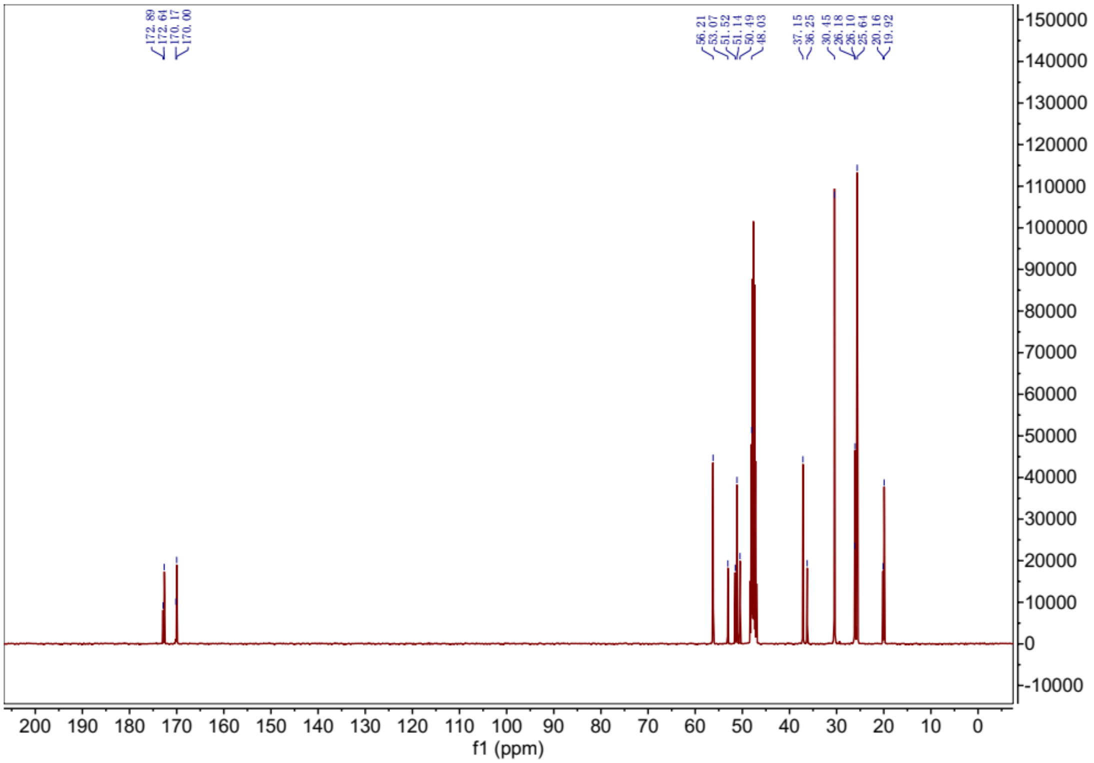
172.89
172.61
170.17
170.00
56.21
53.07
51.52
51.14
50.49
48.03
37.15
36.25
30.45
26.18
26.10
25.64
20.16
19.92
f1 (ppm)

Ac-Nasn-OMe

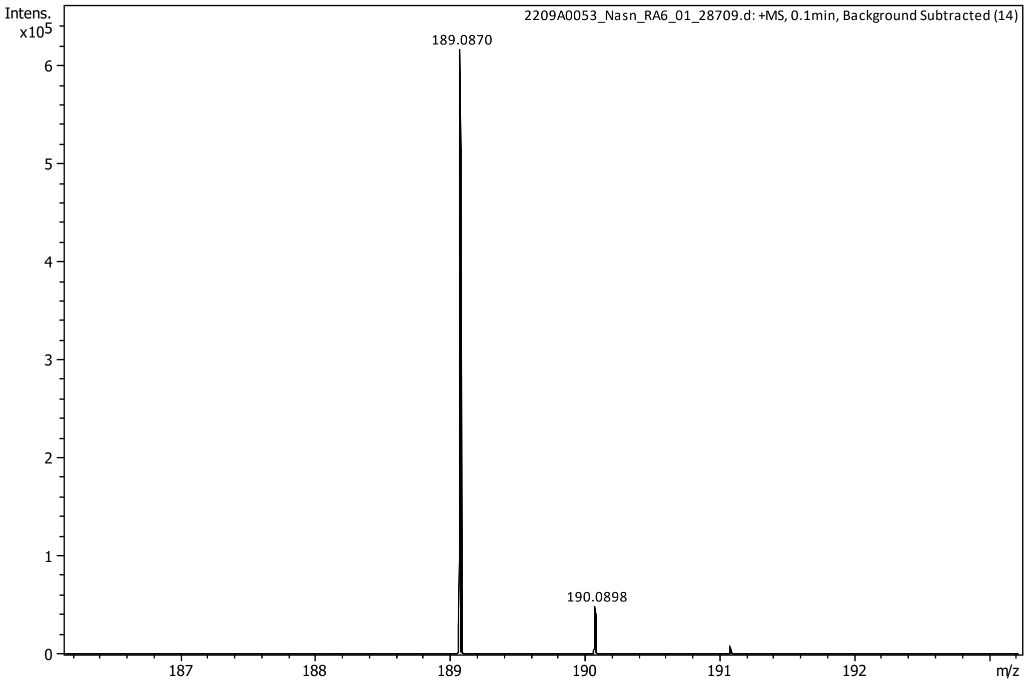
Intens.
x10^5
2209A0053_Nasn_RA6_01_28709.d: +MS, 0.1min, Background Subtracted (14)
189.0870
190.0898
m/z

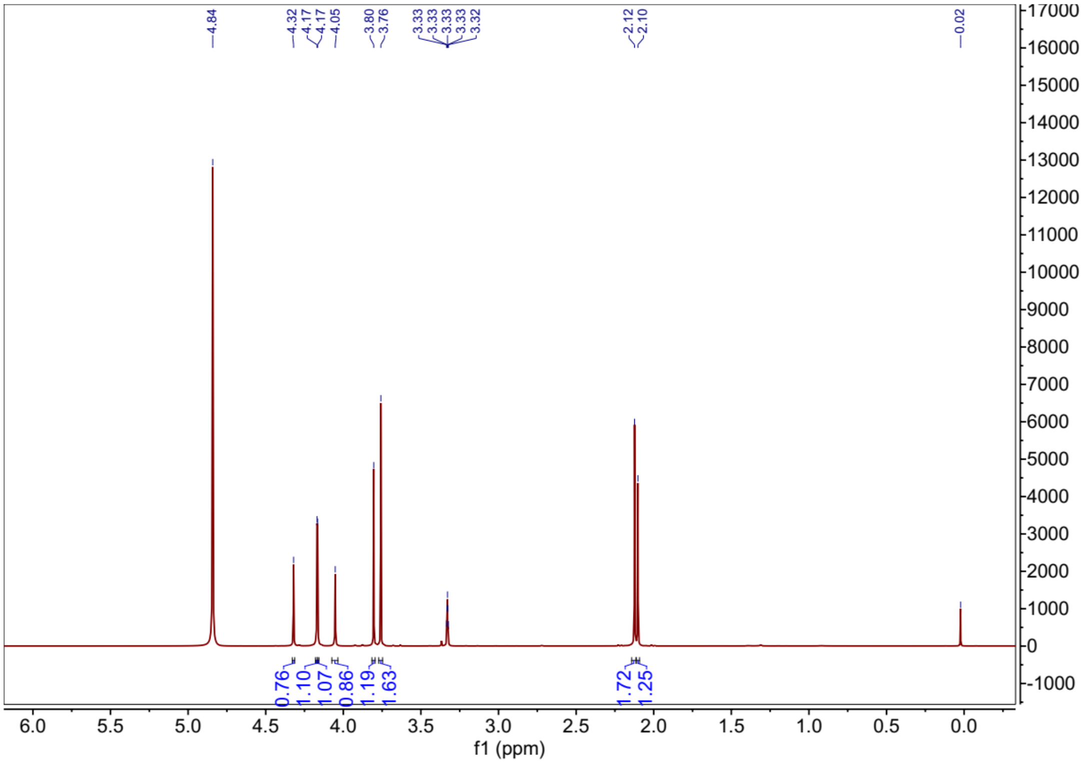
4.84
4.32
4.17
4.17
4.05
3.80
3.76
3.33
3.33
3.33
3.33
3.32
2.12
2.10
0.02
0.76
1.10
1.07
0.86
1.19
1.63
1.72
1.25
f1 (ppm)

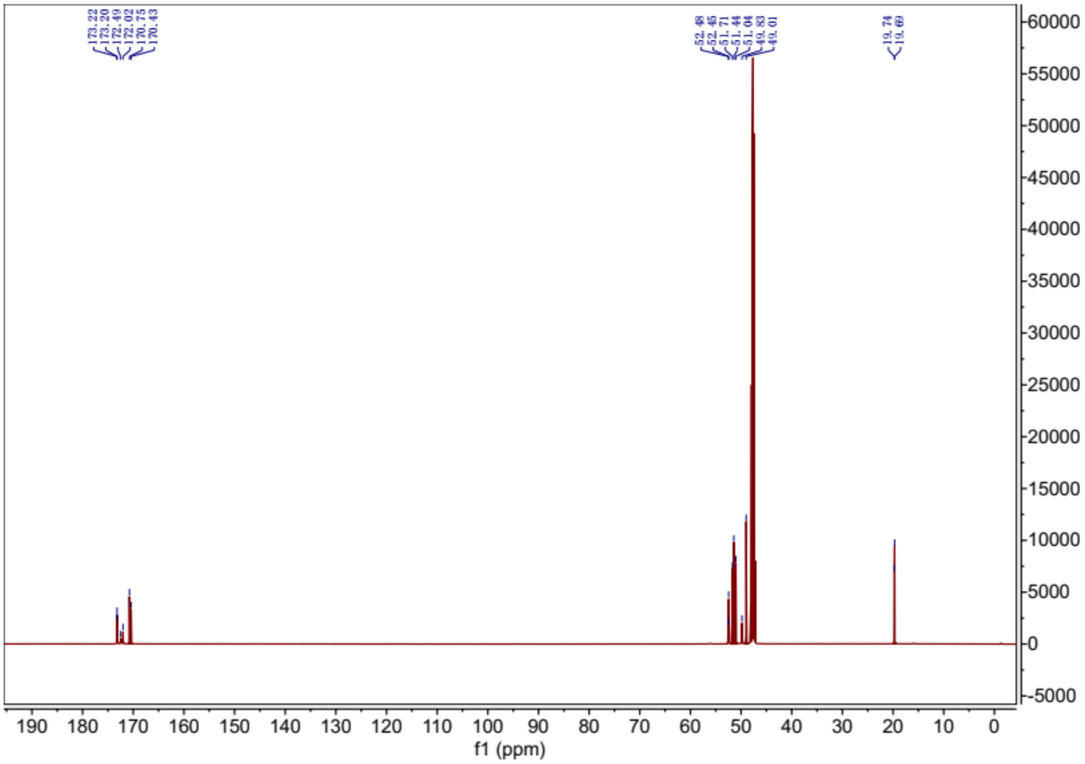
173.22
173.20
172.49
172.02
170.75
170.43
52.48
52.45
51.71
51.44
51.04
49.83
49.01
19.74
19.69
f1 (ppm)

Ac-Nlys-OMe·TFA

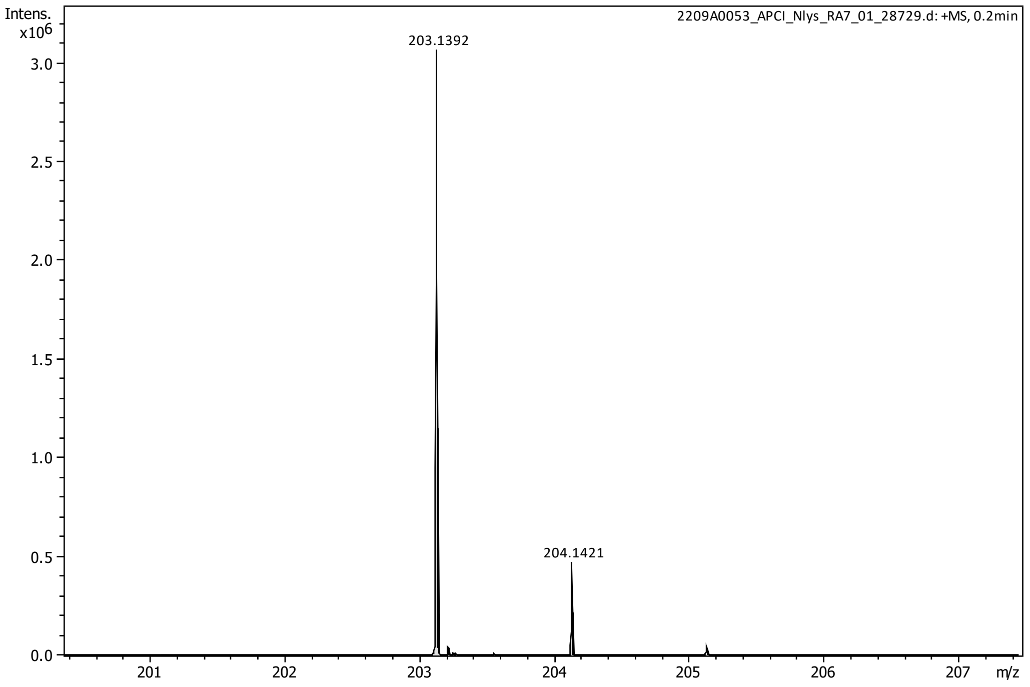

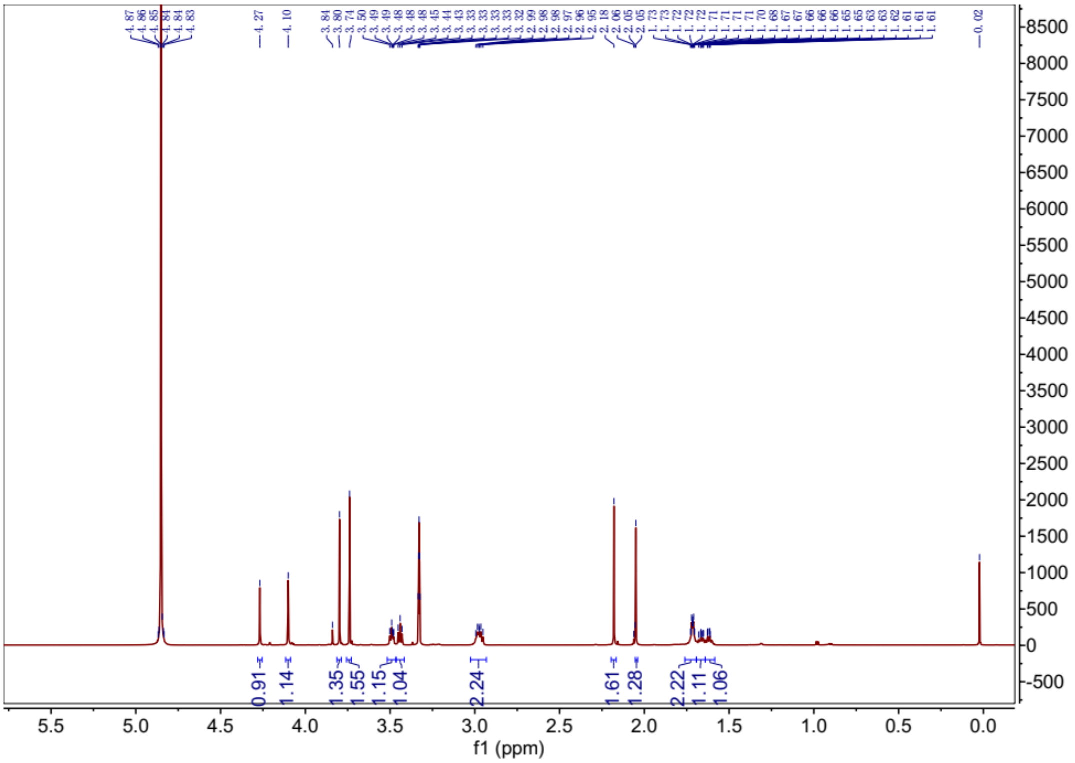

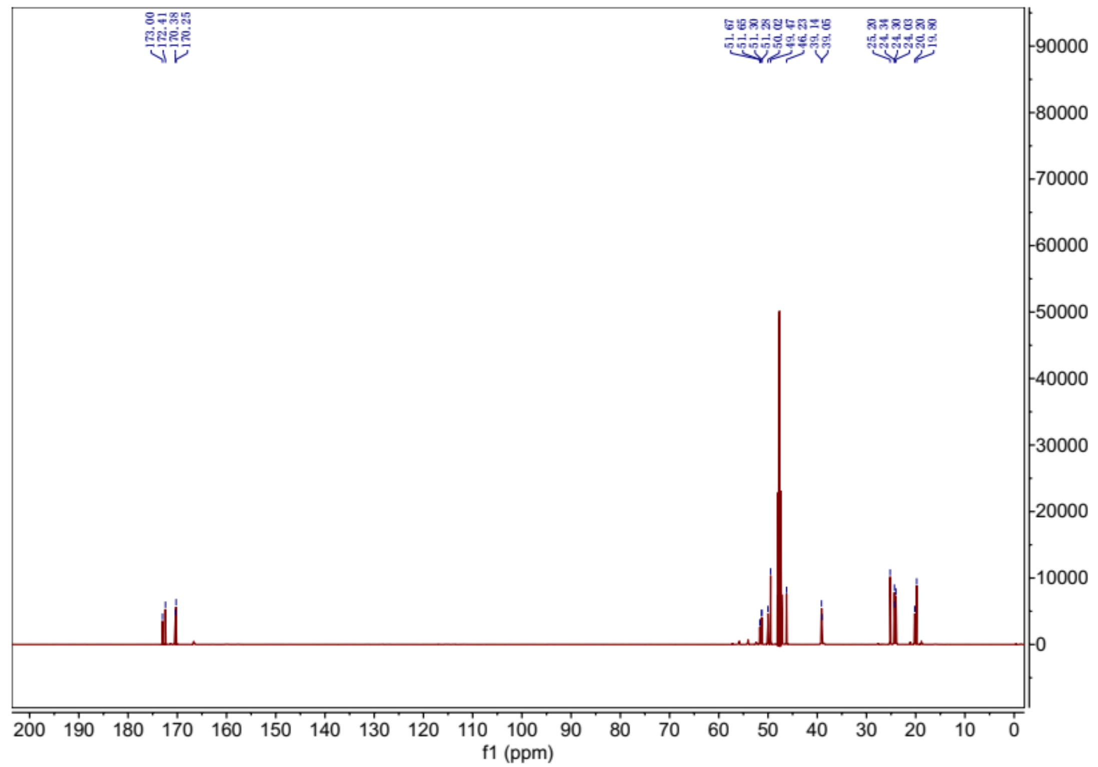

Ac-N2pic-OMe

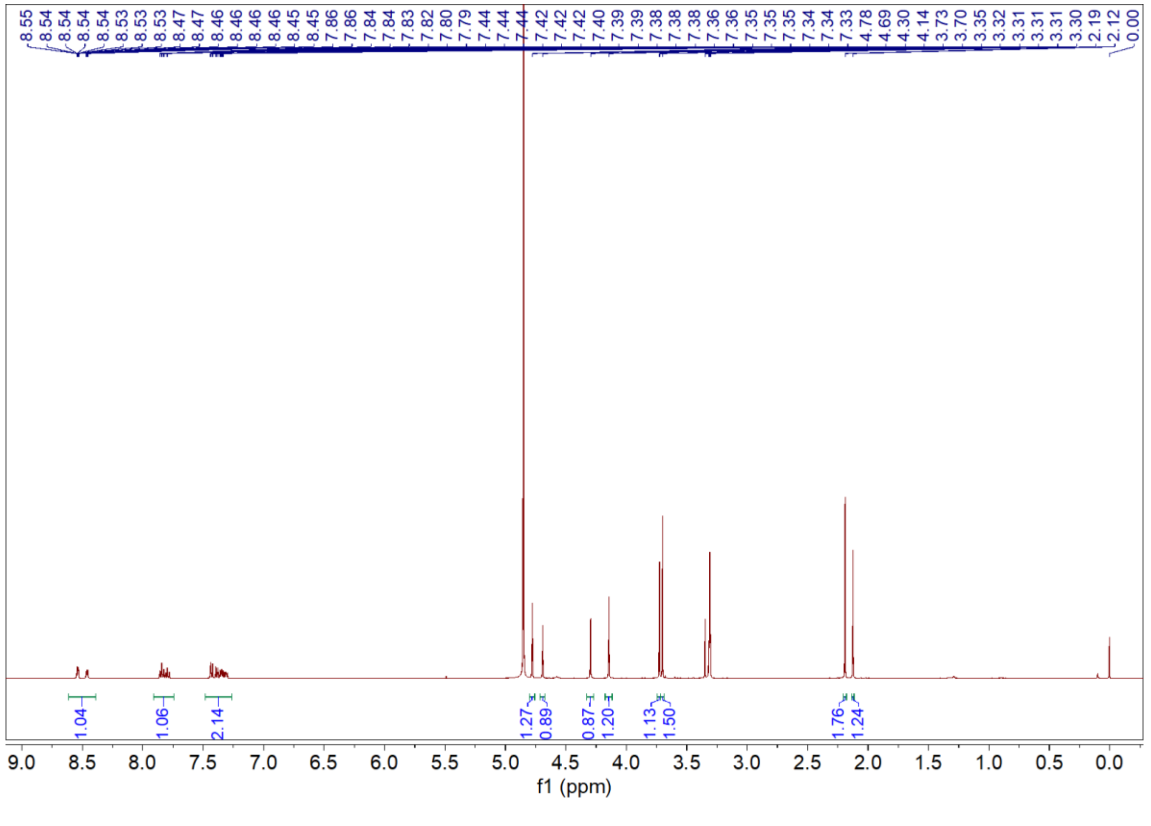

Ac-N2pic-OMe·TFA

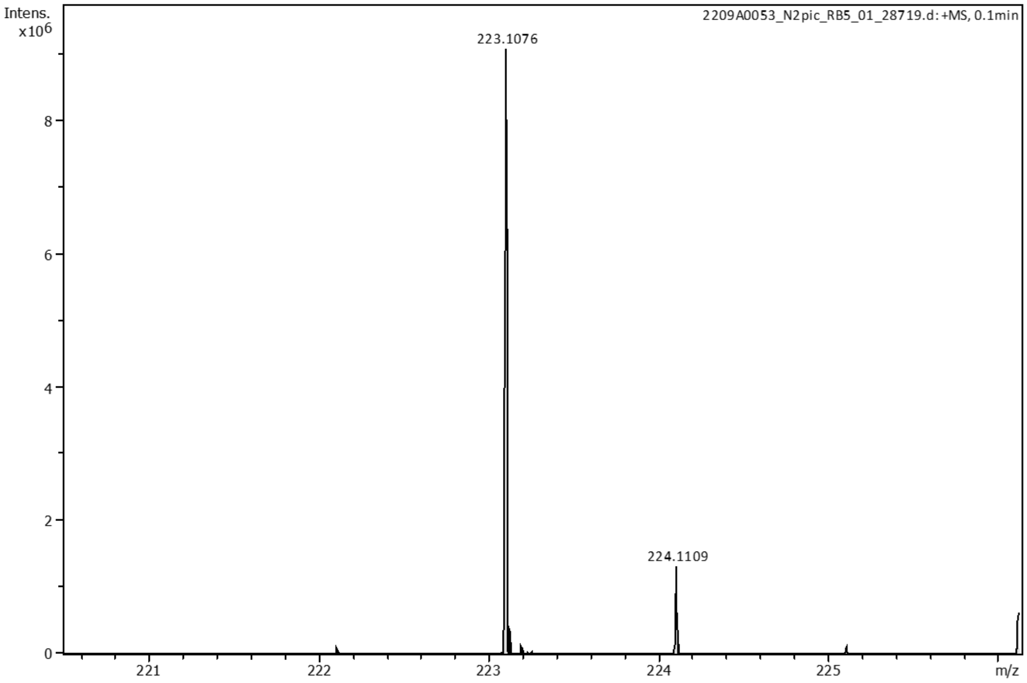

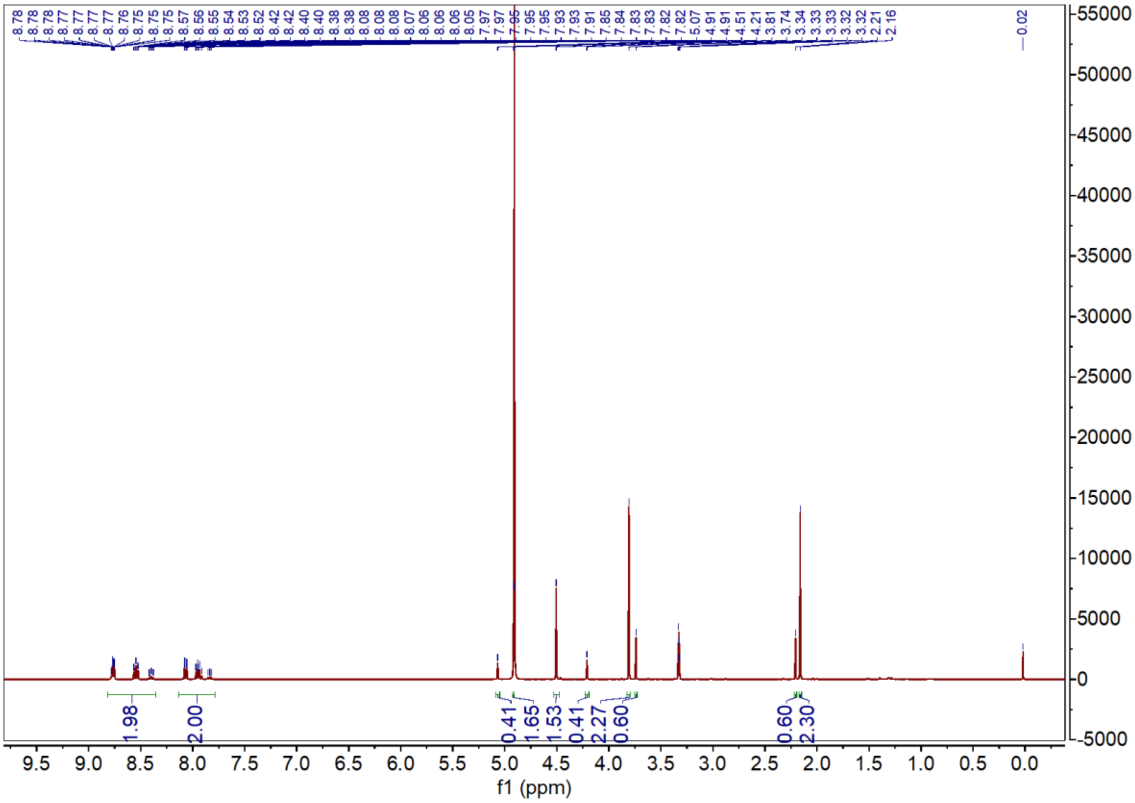

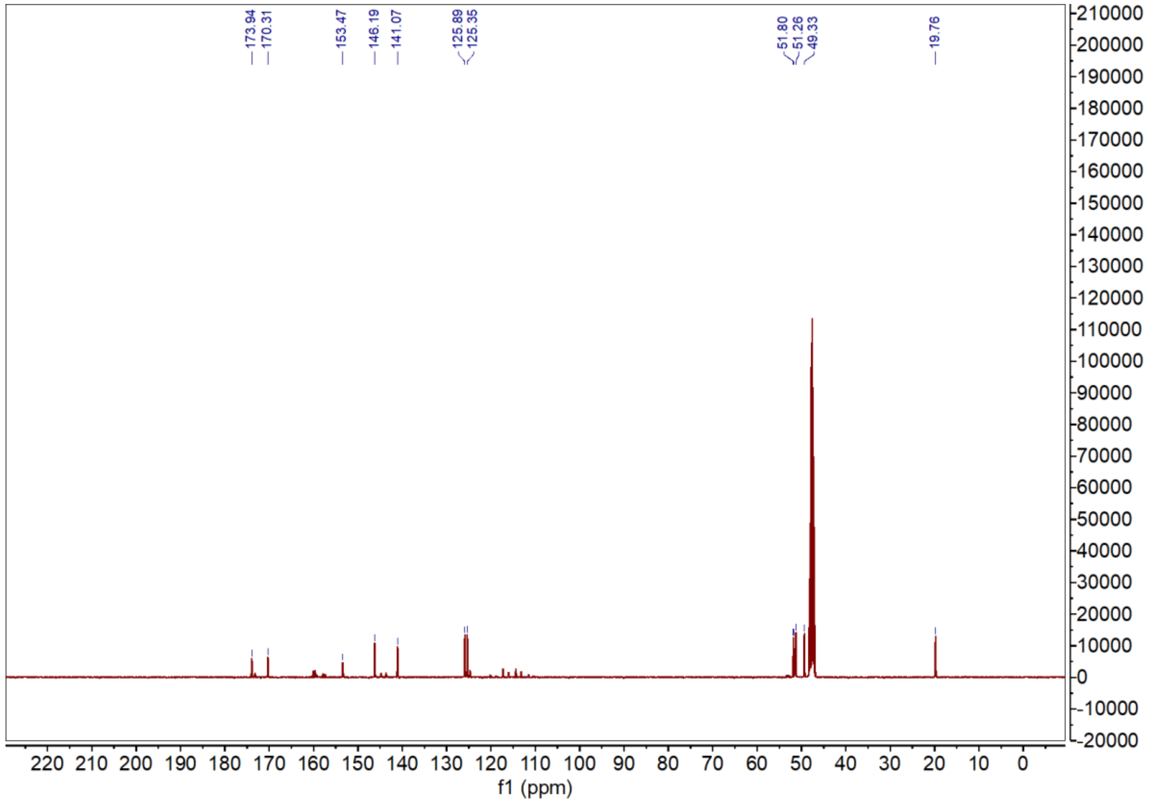

173.94
170.31
153.47
146.19
141.07
125.89
125.35
51.80
51.26
49.33
19.76
f1 (ppm)

Ac-Nbtm^{+}-OMe

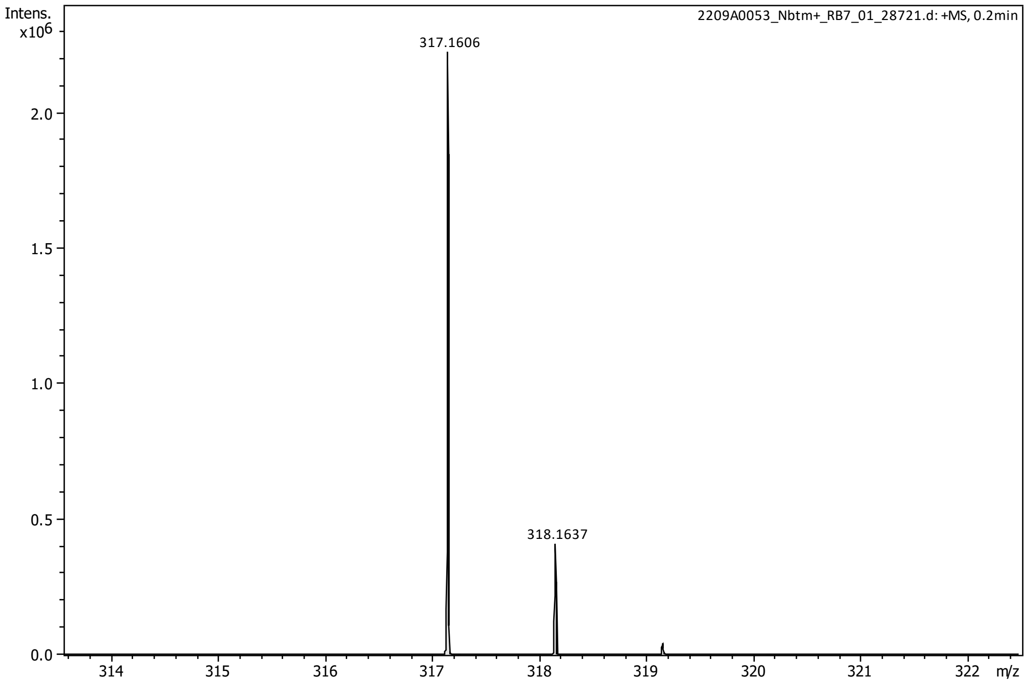

Intens.
x10^6
2209A0053_Nbtm+_RB7_01_28721.d: +MS, 0.2min
317.1606
318.1637
m/z

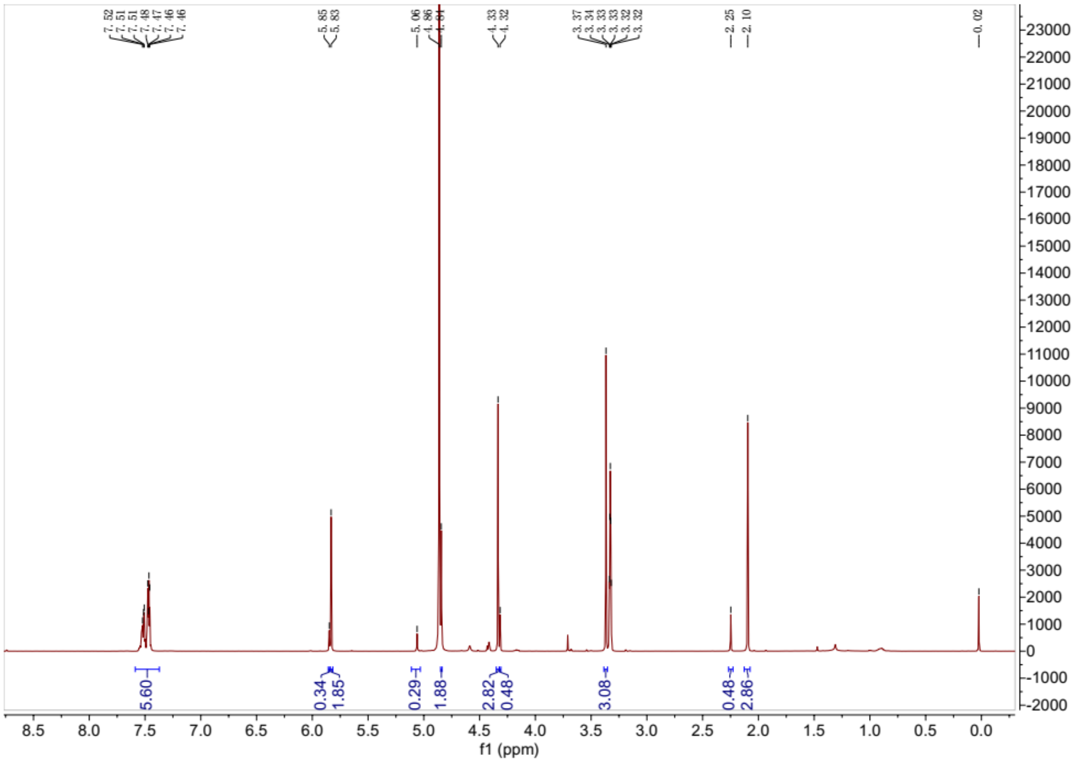
7.52
7.51
7.51
7.48
7.47
7.46
7.46
5.85
5.83
5.06
4.86
4.33
4.32
3.37
3.34
3.33
3.33
3.32
3.32
2.25
2.10
0.02
5.60
0.34
1.85
0.29
1.88
2.82
0.48
3.08
0.48
2.86
f1 (ppm)

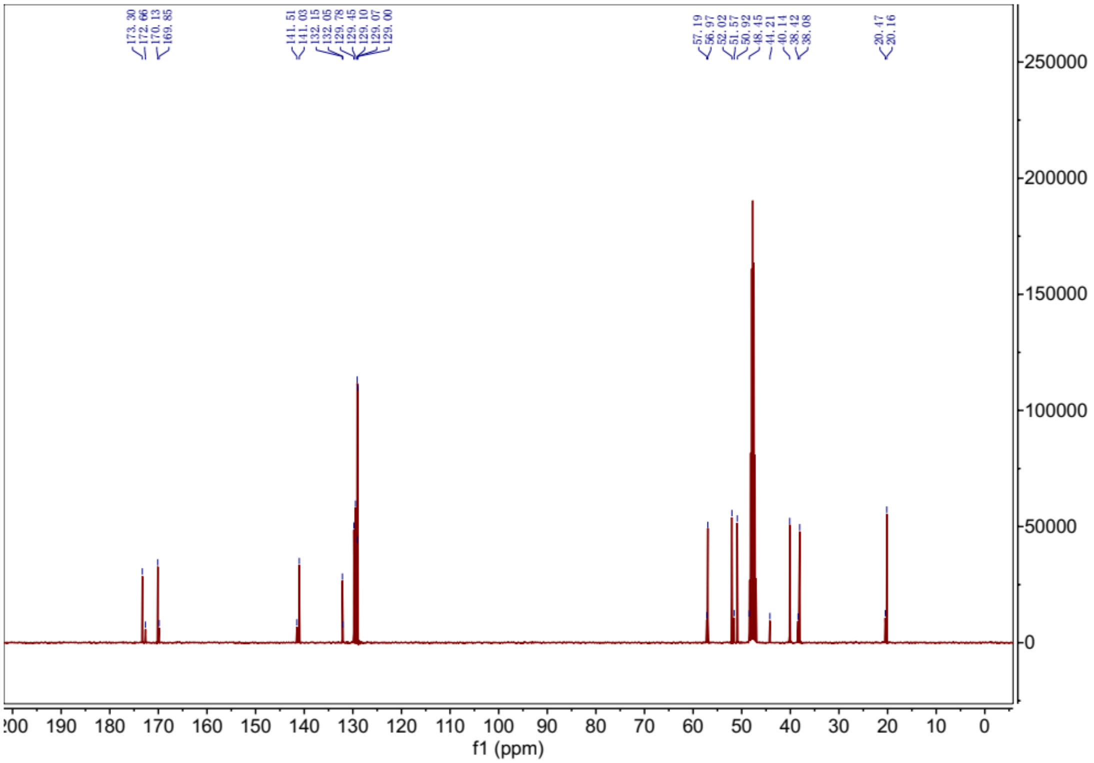
173.30
172.66
170.13
169.85
141.51
141.03
132.15
132.05
129.78
129.45
129.10
129.07
129.00
57.19
56.97
52.02
51.57
50.92
48.45
44.21
40.14
38.42
38.08
20.47
20.16
f1 (ppm)

Ac-Nme$_2$ae-OMe

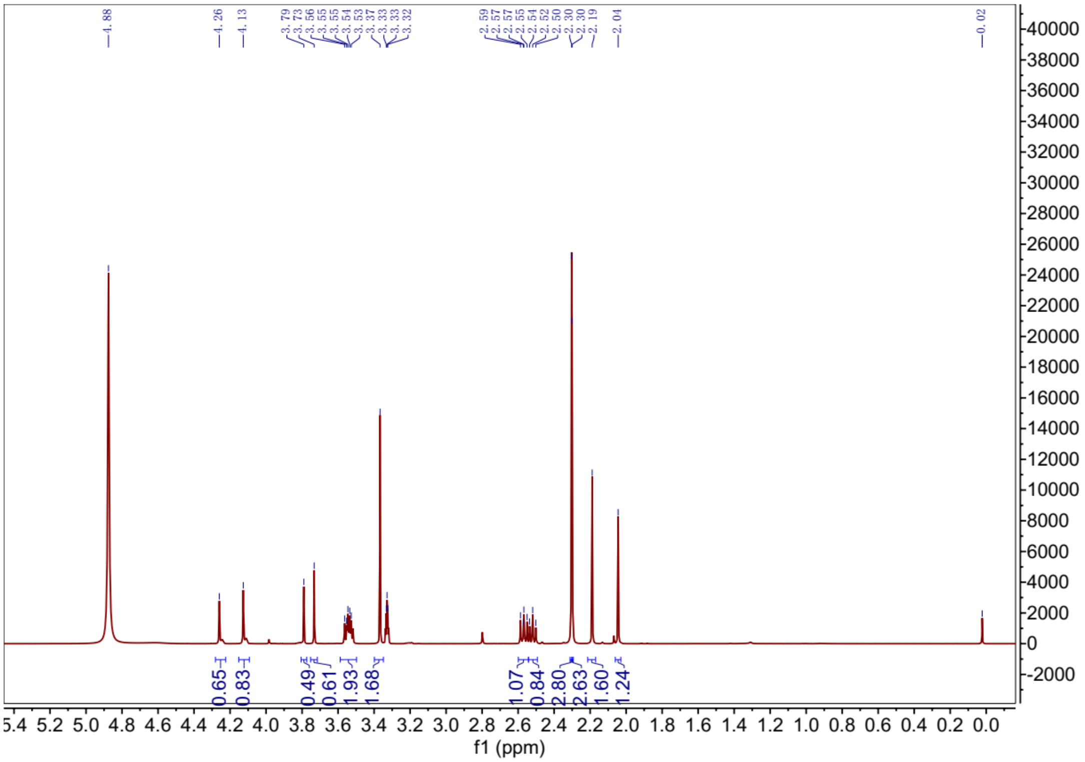

Ac-Nme$_2$ae-OMe·TFA

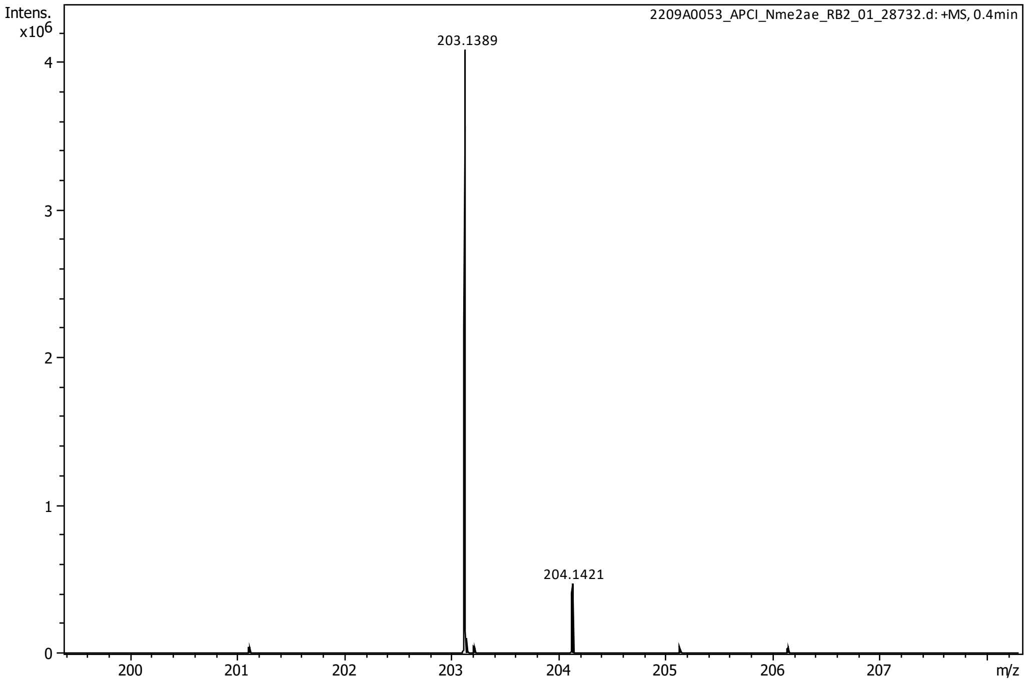

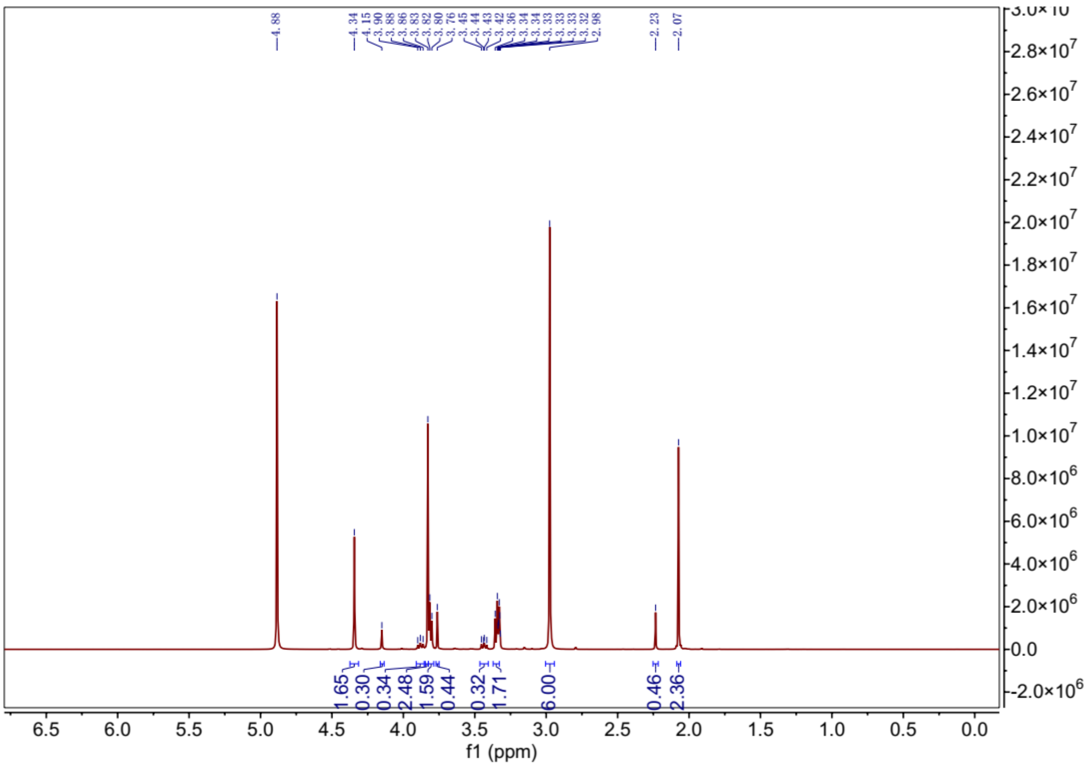

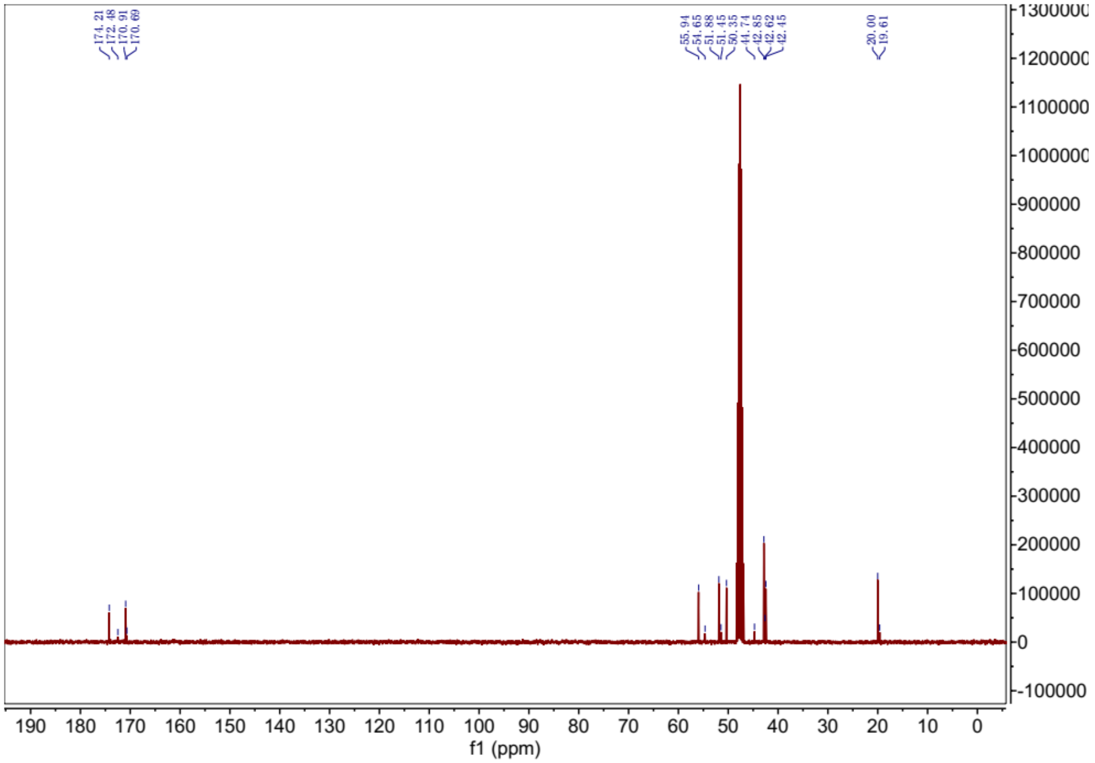

Ac-Net$_2$ae-OMe

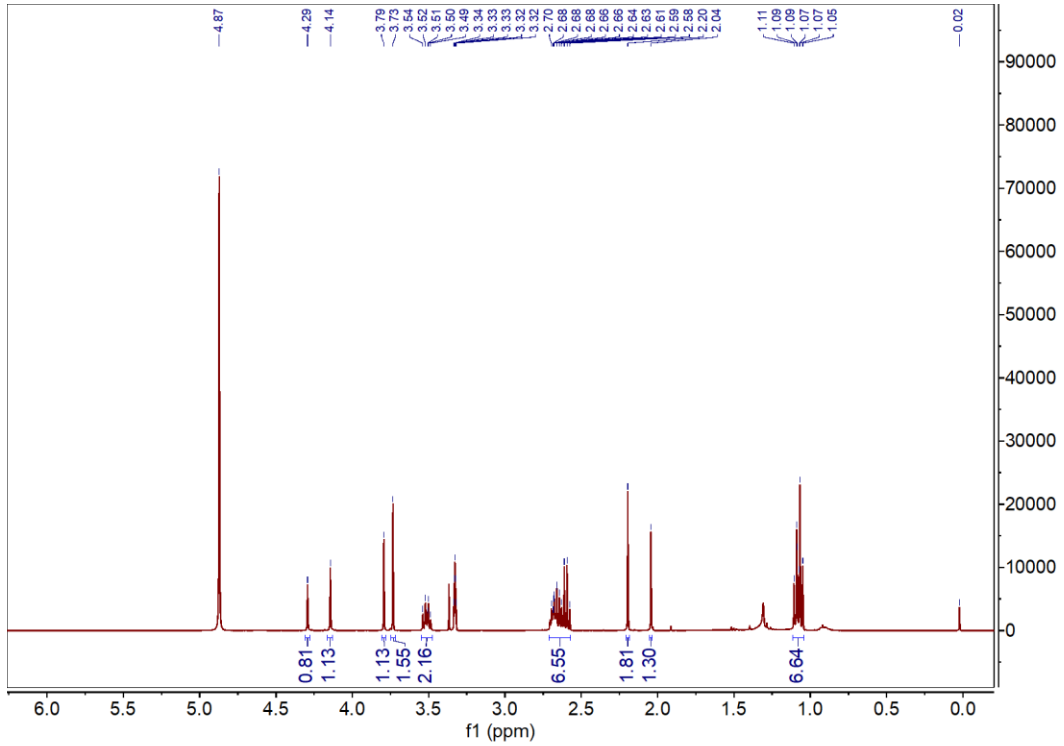

Ac-Net$_2$ae-OMe·TFA

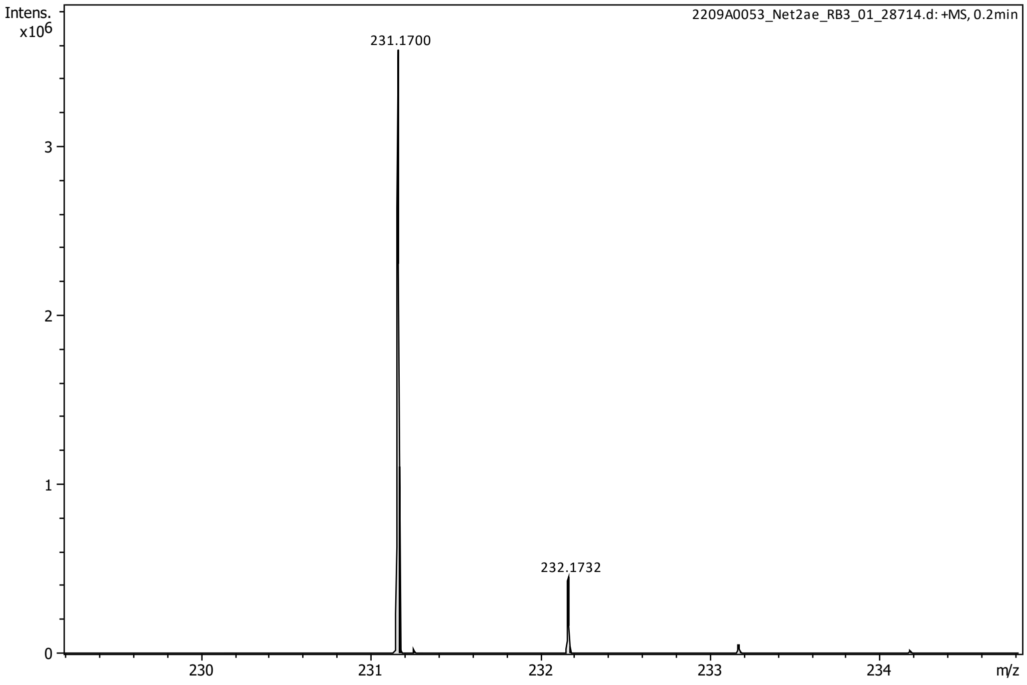

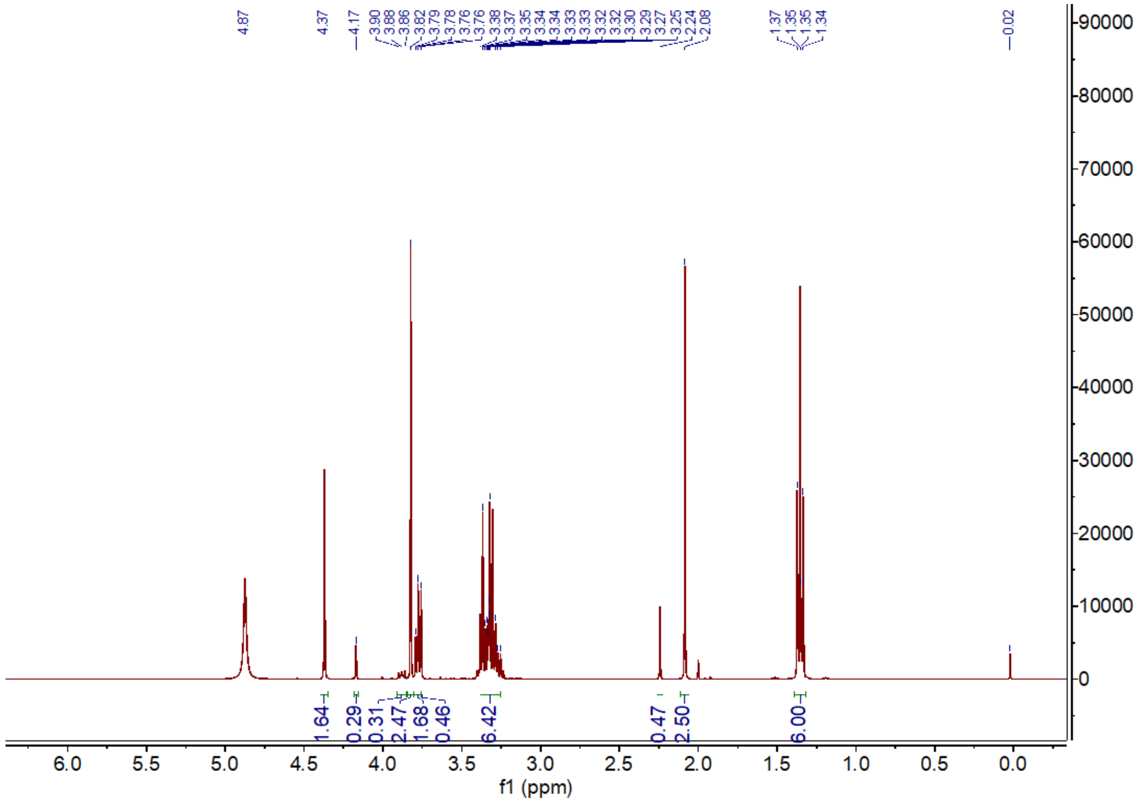

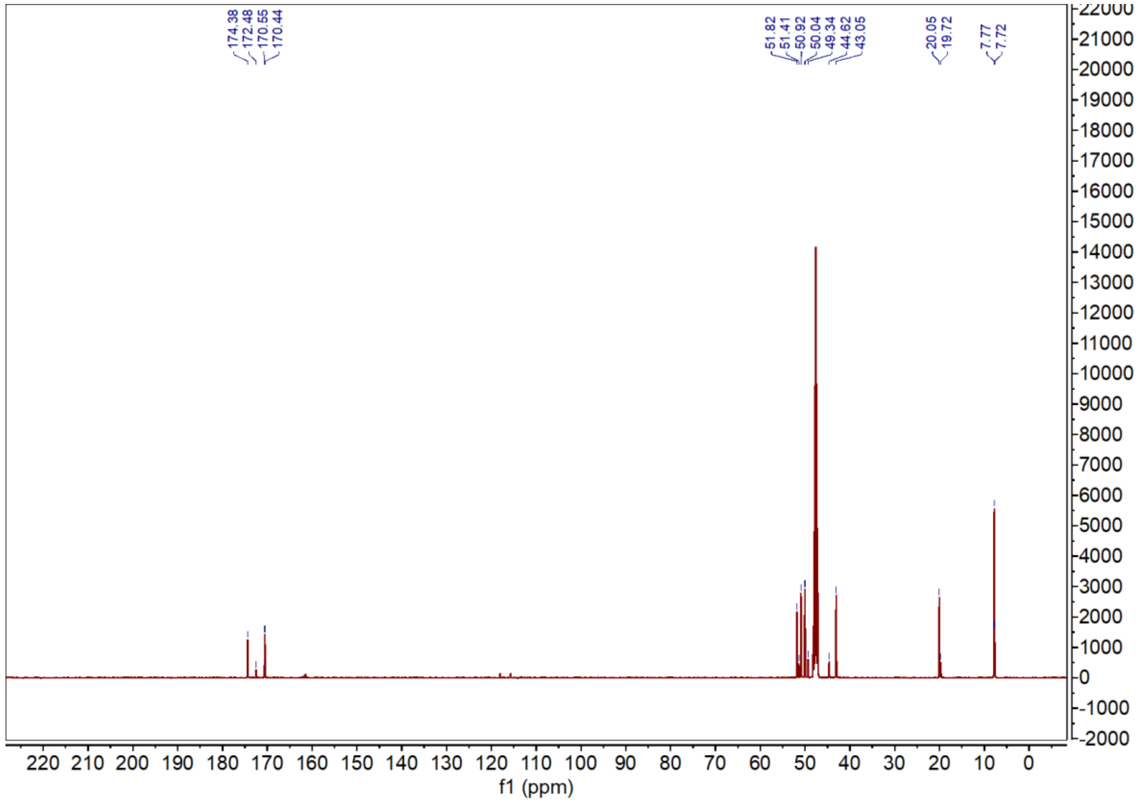

Ac-N^{i}pr$_2$ae-OMe

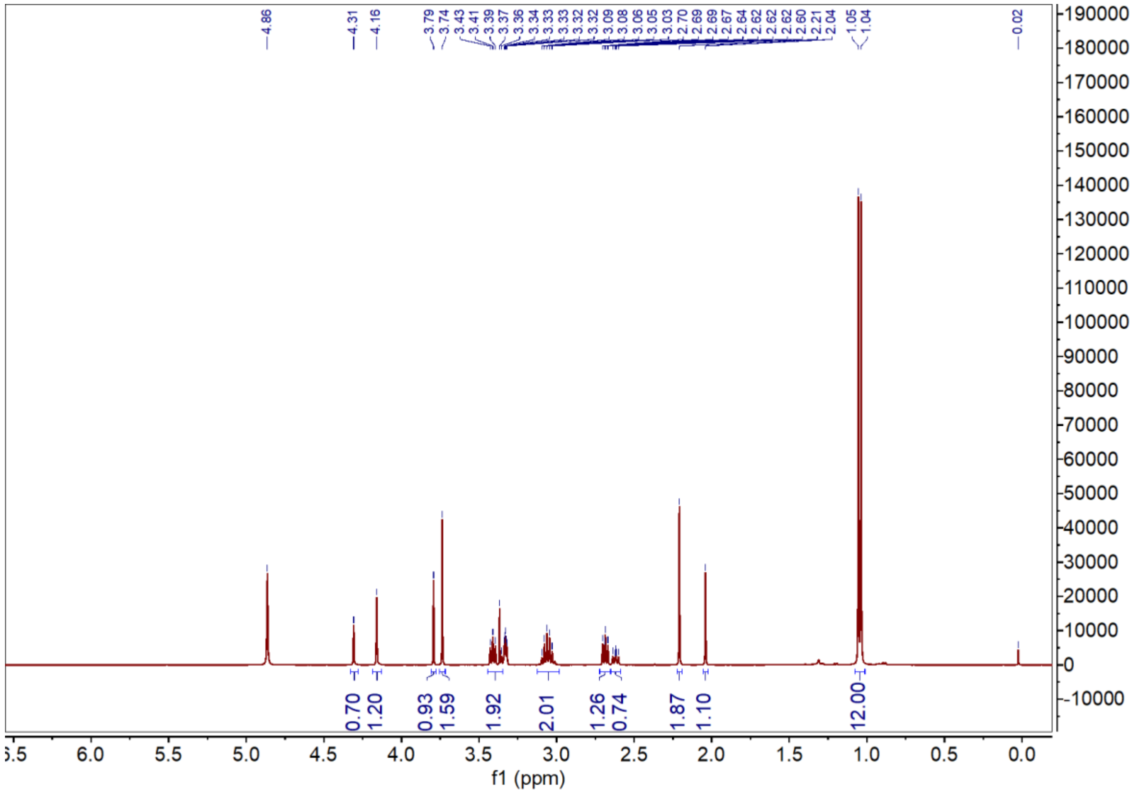

Ac-N^{i}pr$_2$ae-OMe·TFA

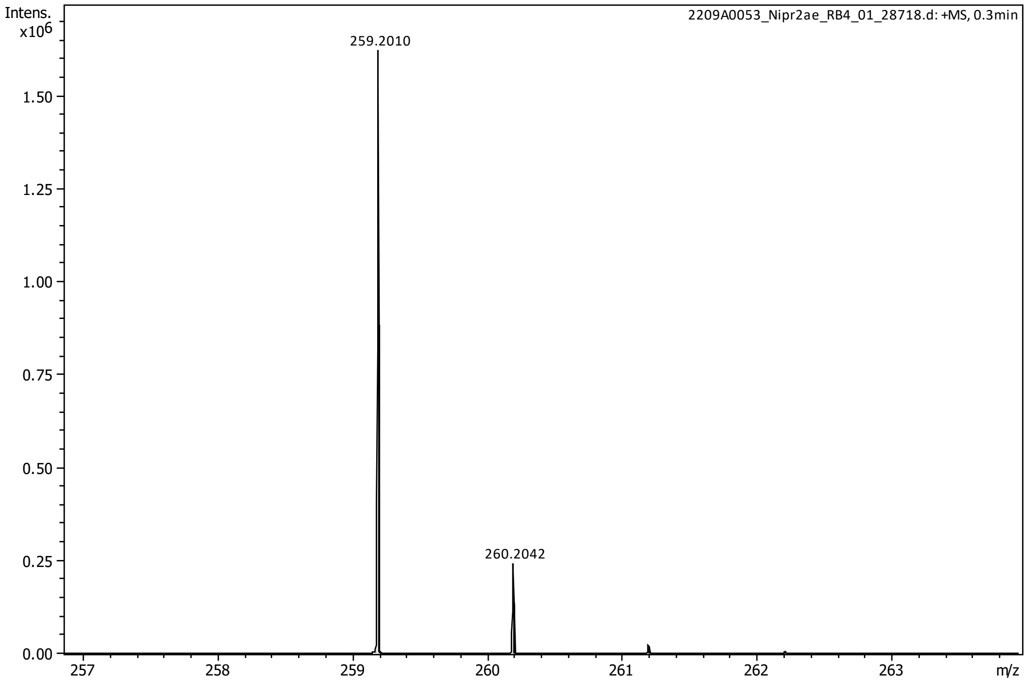

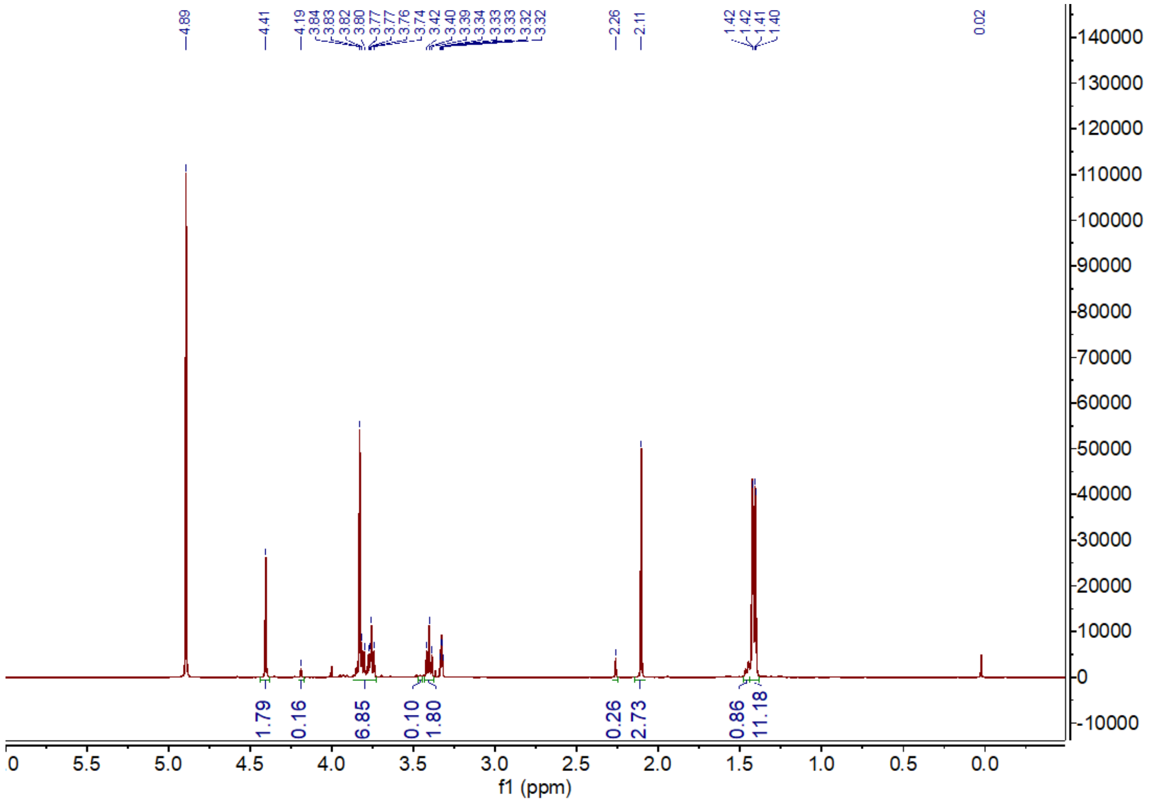

4.89
4.41
4.19
3.84
3.83
3.82
3.80
3.77
3.76
3.74
3.42
3.40
3.39
3.34
3.33
3.33
3.32
3.32
2.26
2.11
1.42
1.42
1.41
1.40
0.02
1.79
0.16
6.85
0.10
1.80
0.26
2.73
0.86
11.18
f1 (ppm)

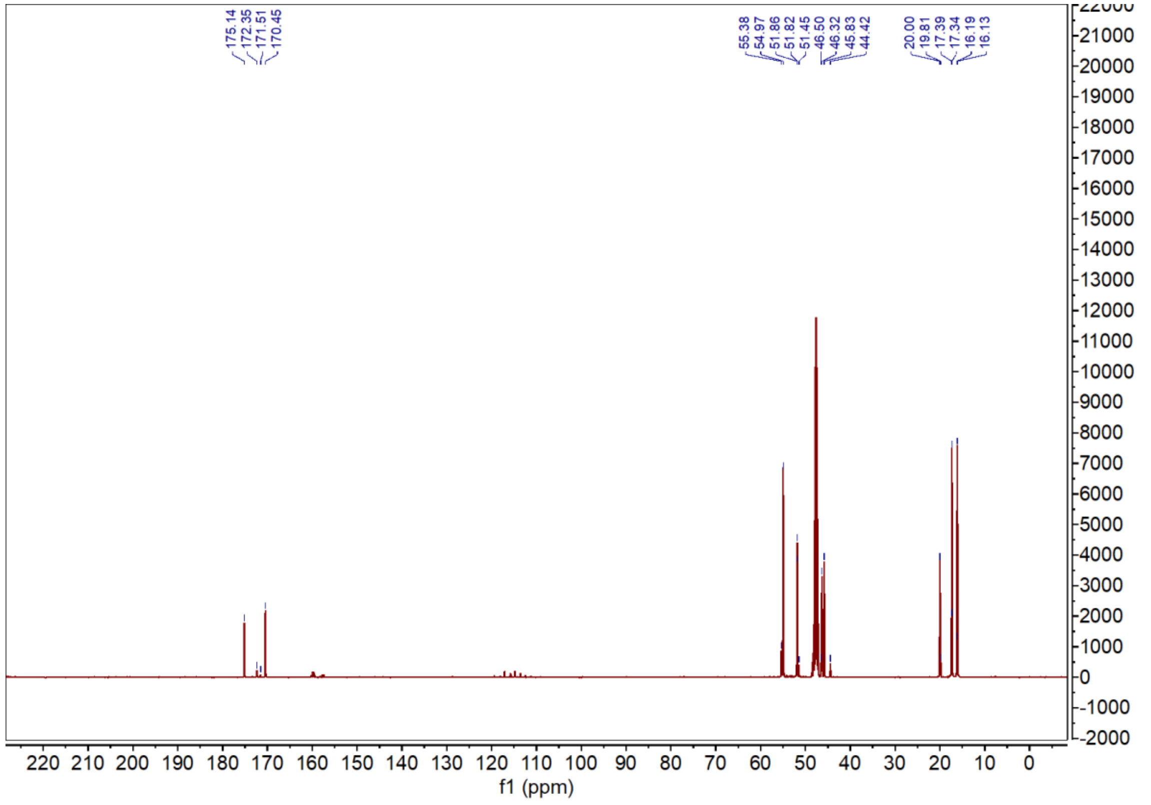

175.14
172.35
171.51
170.45
55.38
54.97
51.86
51.82
51.45
46.50
46.32
45.83
44.42
20.00
19.81
17.39
17.34
16.19
16.13
f1 (ppm)

Section 4: CD and MALDI Spectra of All Peptides

X-CMP peptides

Pro-CMP (in PBS)

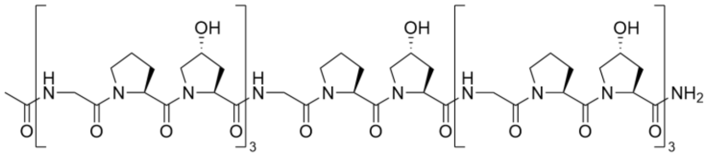

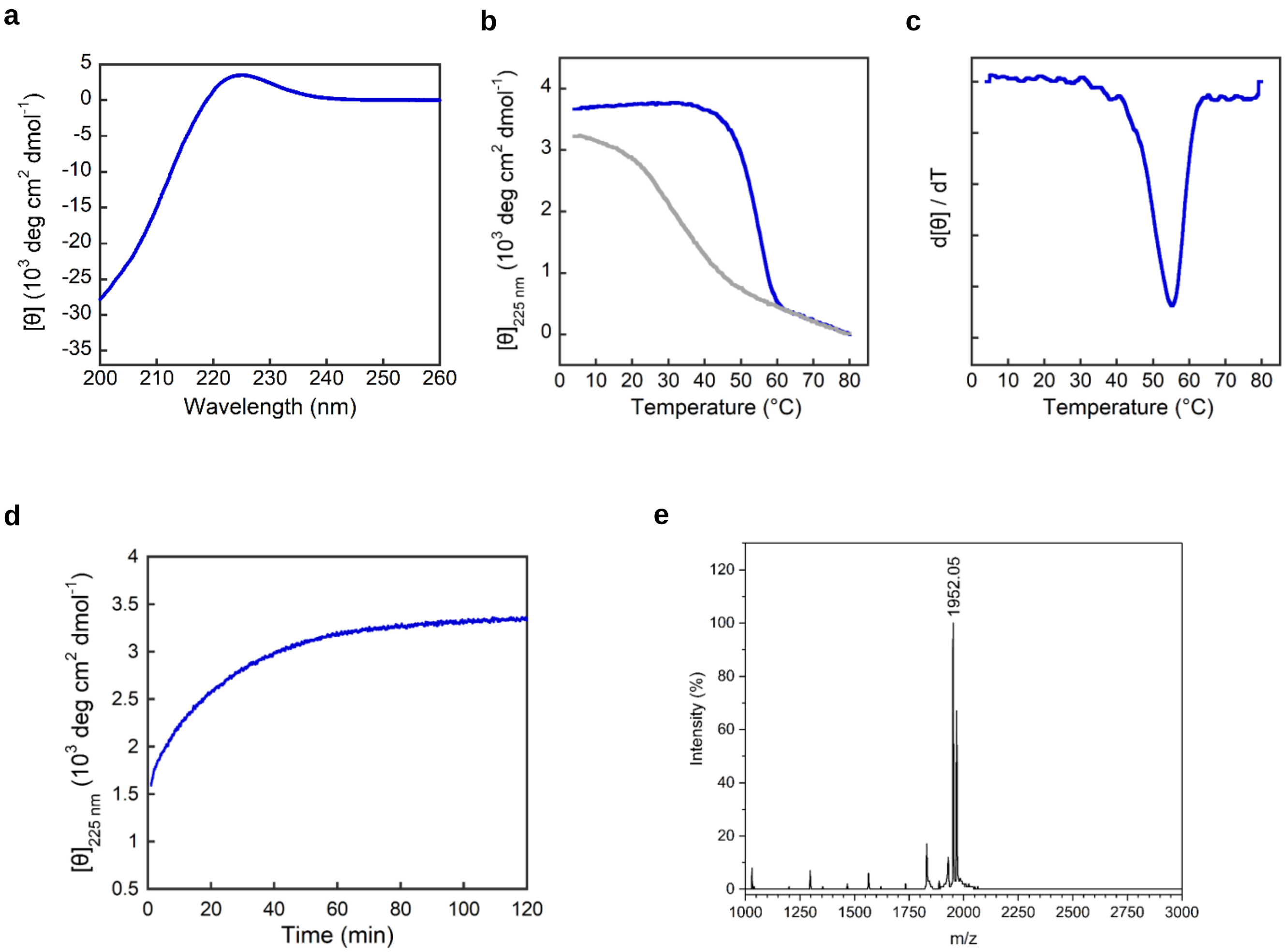

a, The CD spectrum in PBS buffer at 4 °C.

b, The CD thermal unfolding (blue) and cooling (gray) curves in PBS buffer.

c, The first derivative of the thermal unfolding curve, T_m = 55 °C.

d, The CD refolding curve in PBS buffer at 4 °C.

e, MALDI-MS, calculated: 1951.89 $[M+Na]^+$, observed: 1952.05 $[M+Na]^+$.

Pro-CMP (in 1 mM HCl)

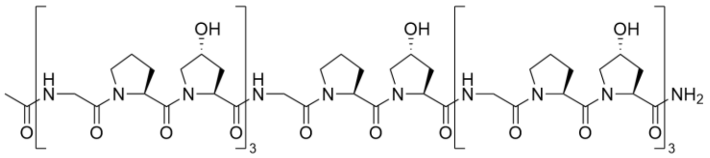

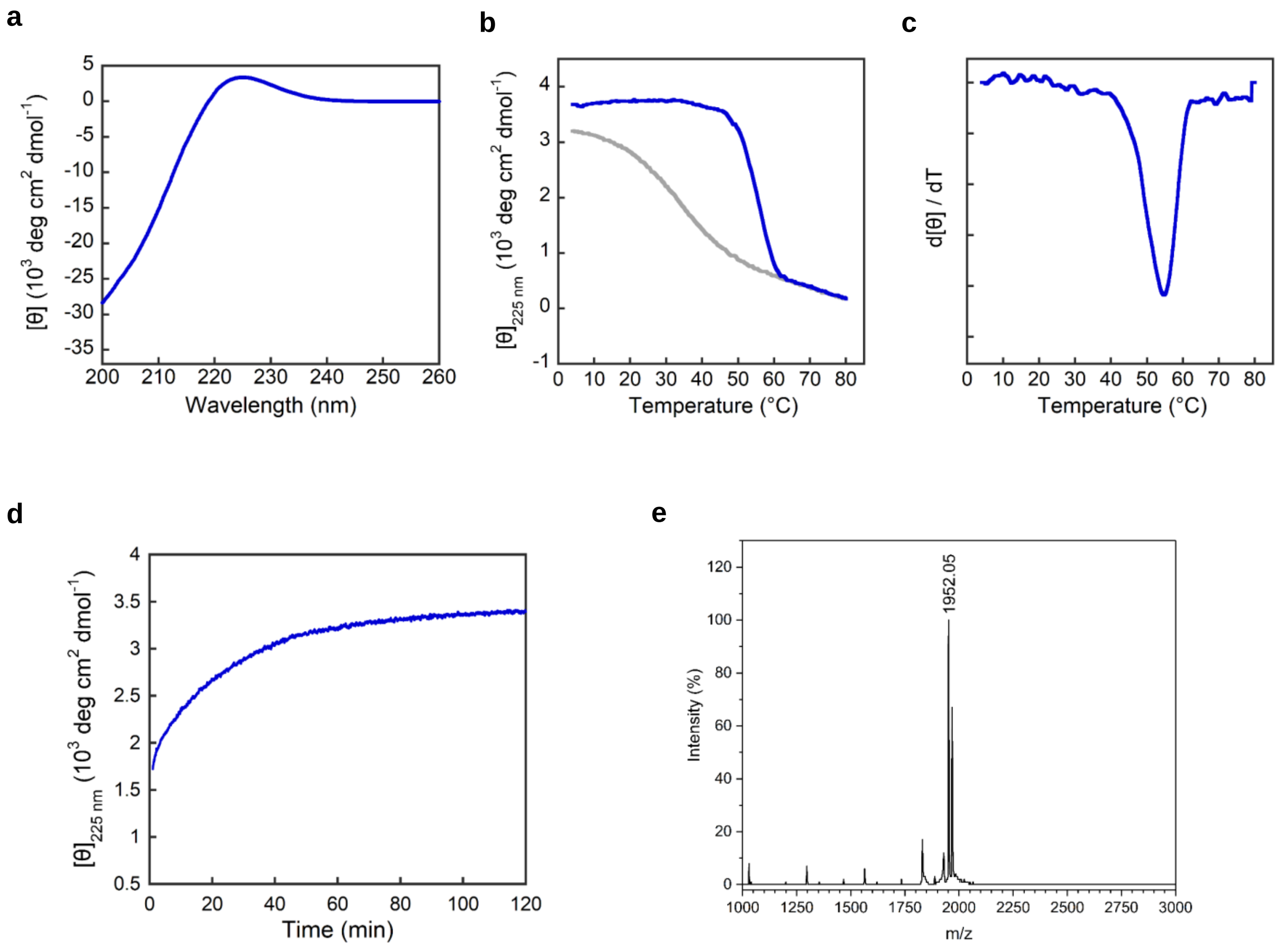

a, The CD spectrum in 1 mM HCl solution at 4 °C.

b, The CD thermal unfolding (blue) and cooling (gray) curves in 1 mM HCl solution.

c, The first derivative of the thermal unfolding curve, T_m = 55 °C.

d, The CD refolding curve in 1 mM HCl solution at 4 °C.

e, MALDI-MS, calculated: 1951.89 $[M+Na]^+$, observed: 1952.05 $[M+Na]^+$.

Gly-CMP (in PBS)

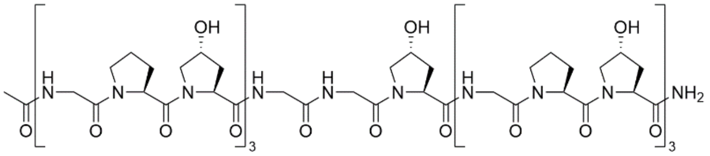

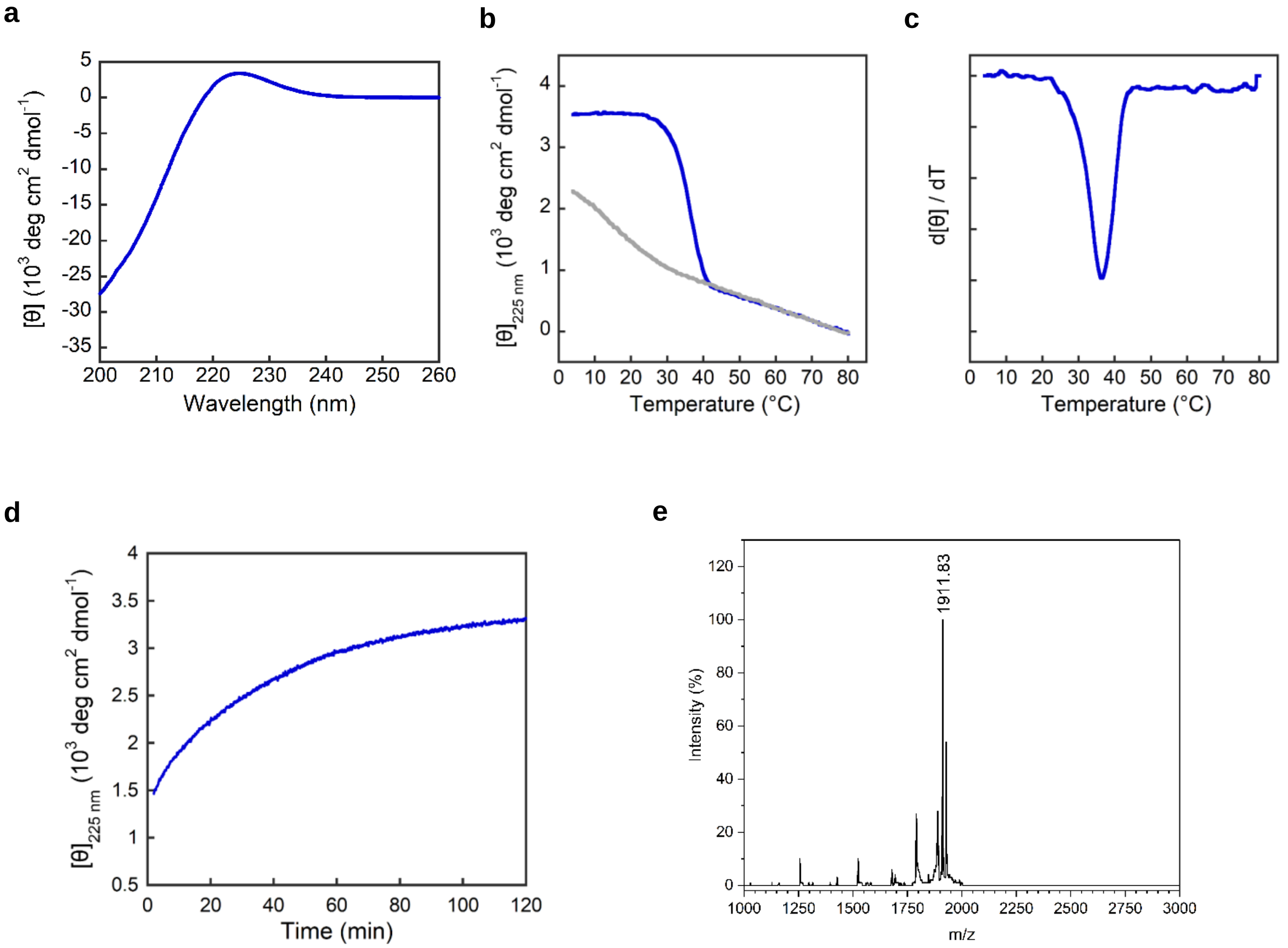

a, The CD spectrum in PBS buffer at 4 °C.

b, The CD thermal unfolding (blue) and cooling (gray) curves in PBS buffer.

c, The first derivative of the thermal unfolding curve, T_m = 36 °C.

d, The CD refolding curve in PBS buffer at 4 °C.

e, MALDI-MS, calculated: 1911.86 [M+Na]$^+$, observed: 1911.83 [M+Na]$^+$.

Ala-CMP (in PBS)

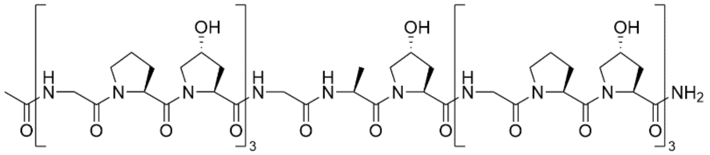

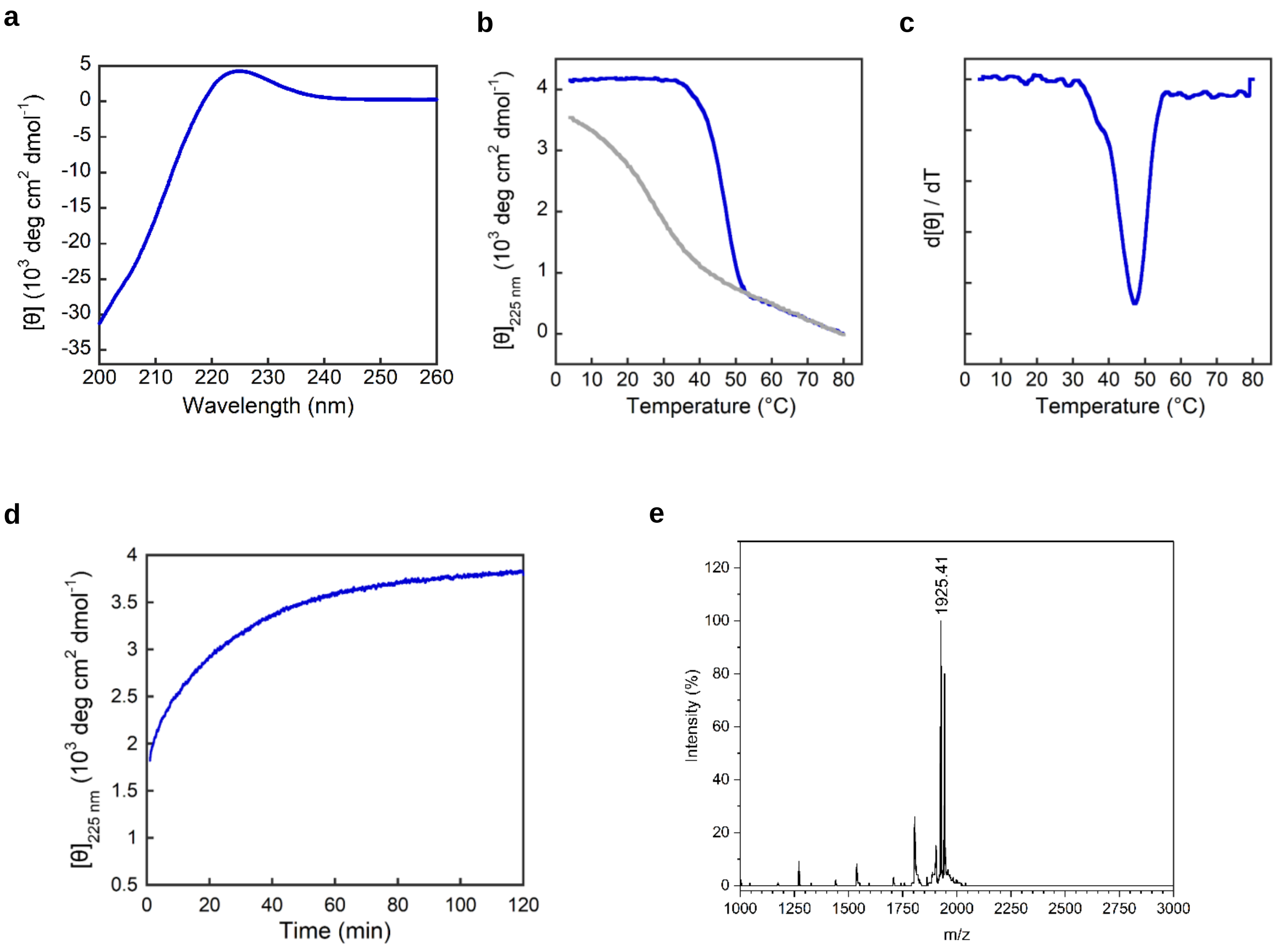

a, The CD spectrum in PBS buffer at 4 °C.

b, The CD thermal unfolding (blue) and cooling (gray) curves in PBS buffer.

c, The first derivative of the thermal unfolding curve, T_m = 47 °C.

d, The CD refolding curve in PBS buffer at 4 °C.

e, MALDI-MS, calculated: 1925.87 $[M+Na]^+$, observed: 1925.41 $[M+Na]^+$.

Leu-CMP (in PBS)

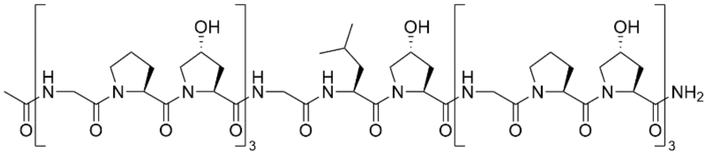

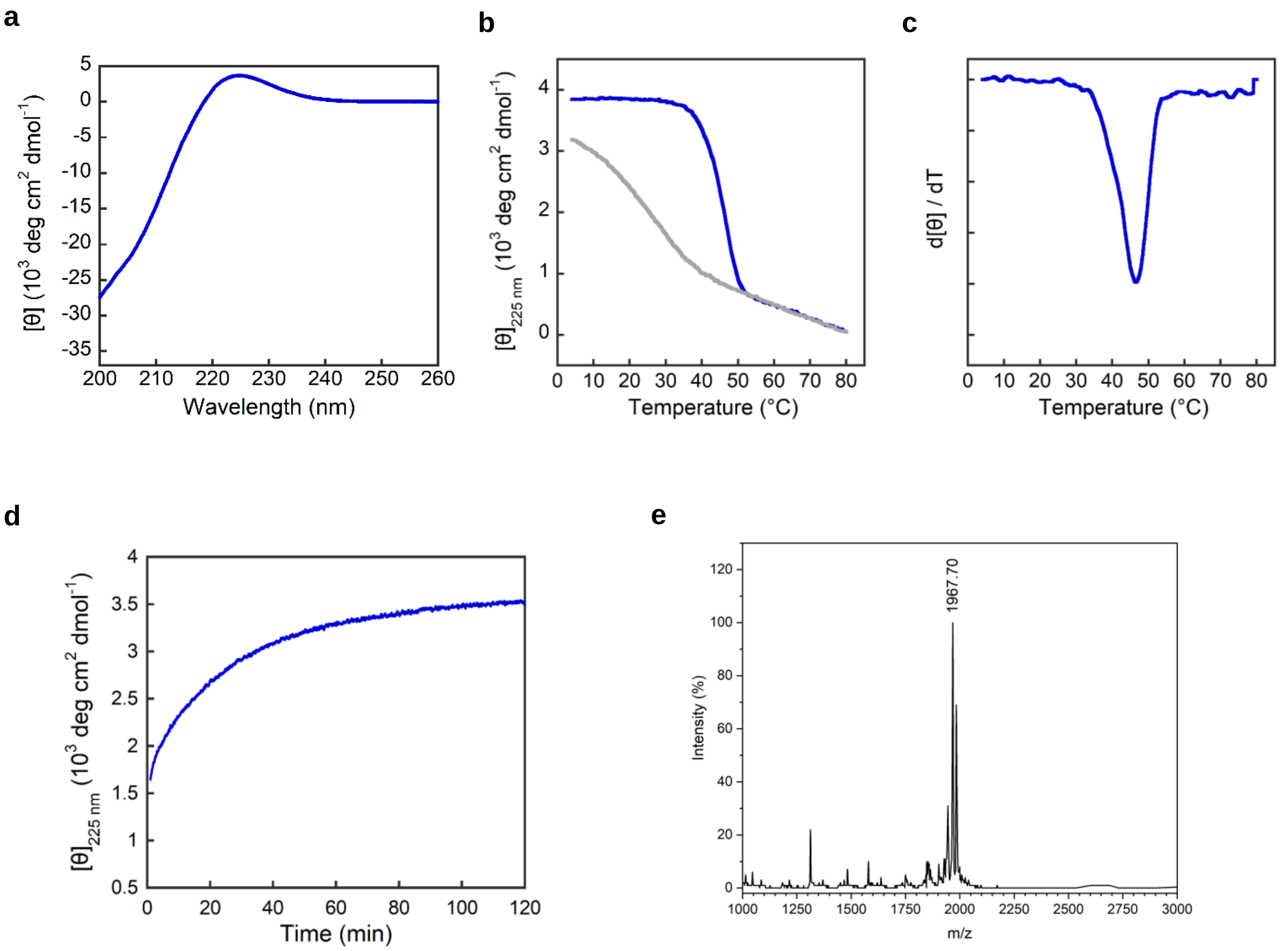

a, The CD spectrum in PBS buffer at 4 °C.

b, The CD thermal unfolding (blue) and cooling (gray) curves in PBS buffer.

c, The first derivative of the thermal unfolding curve, T_m = 47 °C.

d, The CD refolding curve in PBS buffer at 4 °C.

e, MALDI-MS, calculated: 1967.92 $[M+Na]^+$, observed: 1967.70 $[M+Na]^+$.

Phe-CMP (in PBS)

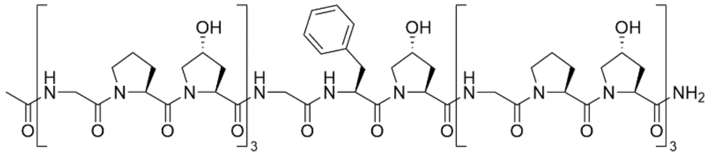

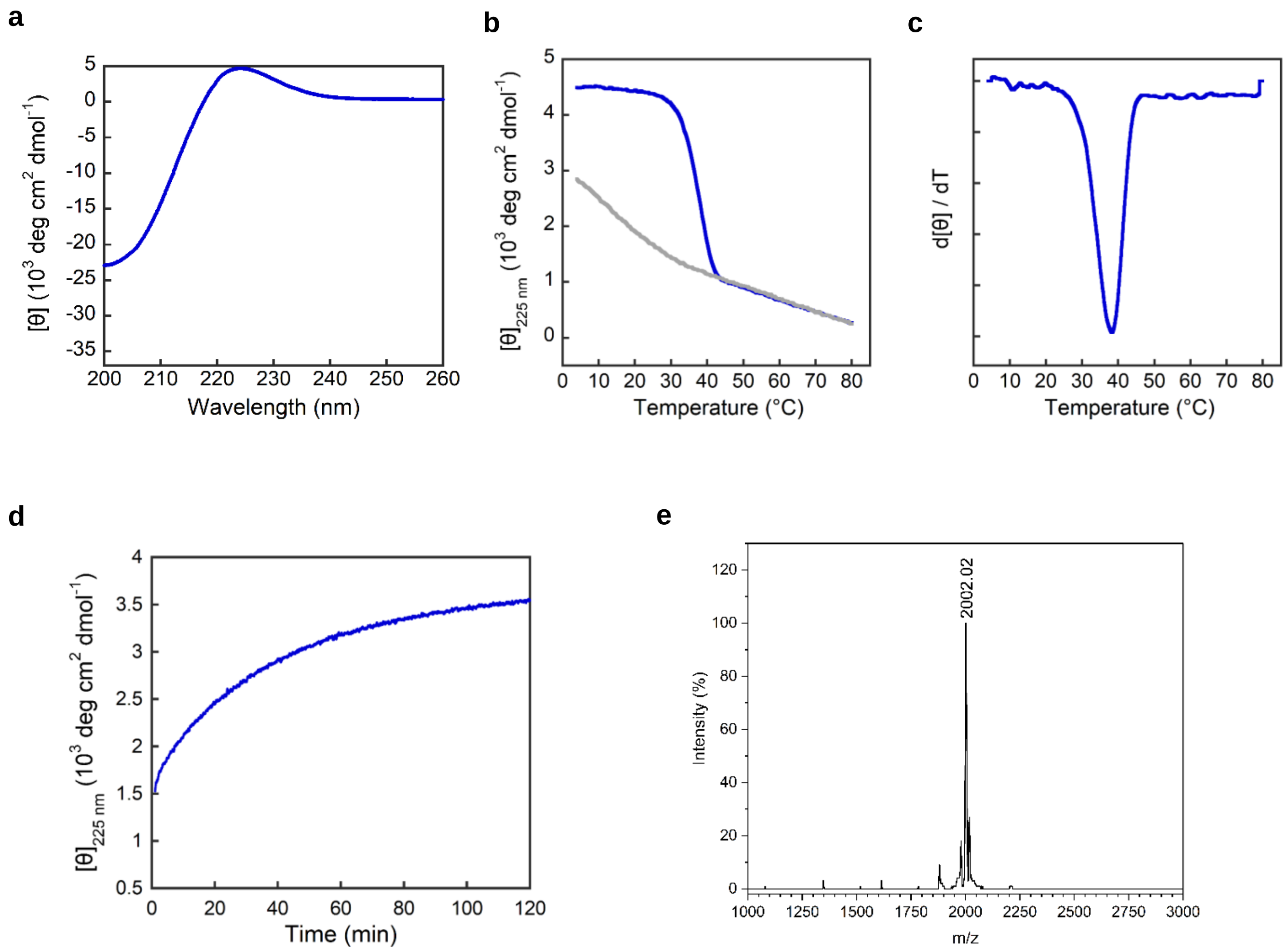

a, The CD spectrum in PBS buffer at 4 °C.

b, The CD thermal unfolding (blue) and cooling (gray) curves in PBS buffer.

c, The first derivative of the thermal unfolding curve, T_m = 38 °C.

d, The CD refolding curve in PBS buffer at 4 °C.

e, MALDI-MS, calculated: 2001.91 $[M+Na]^+$, observed: 2002.02 $[M+Na]^+$.

Asn-CMP (in PBS)

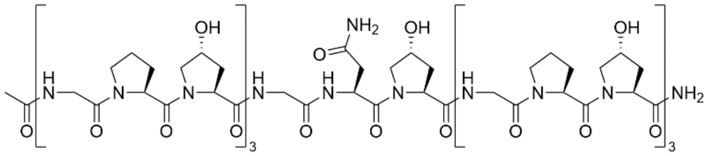

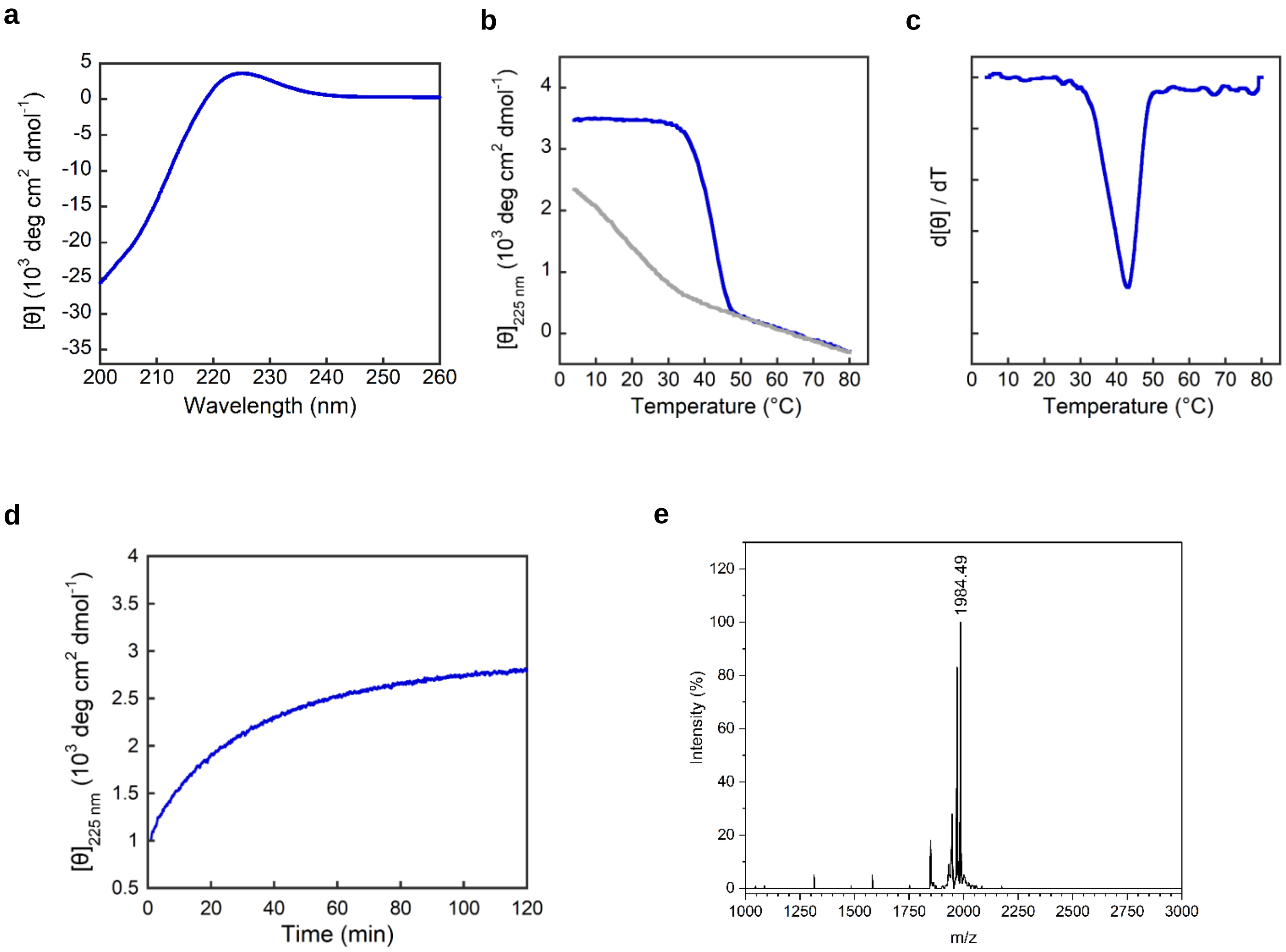

a, The CD spectrum in PBS buffer at 4 °C.

b, The CD thermal unfolding (blue) and cooling (gray) curves in PBS buffer.

c, The first derivative of the thermal unfolding curve, T_m = 43 °C.

d, The CD refolding curve in PBS buffer at 4 °C.

e, MALDI-MS, calculated: 1984.88 $[M+K]^+$, observed: 1984.49 $[M+K]^+$.

Lys-CMP (in PBS)

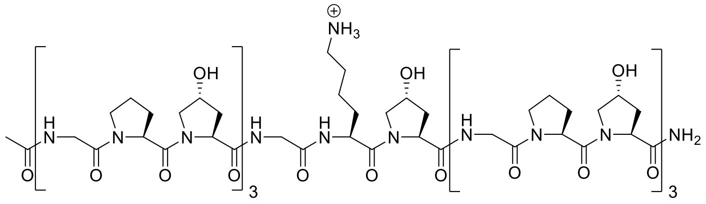

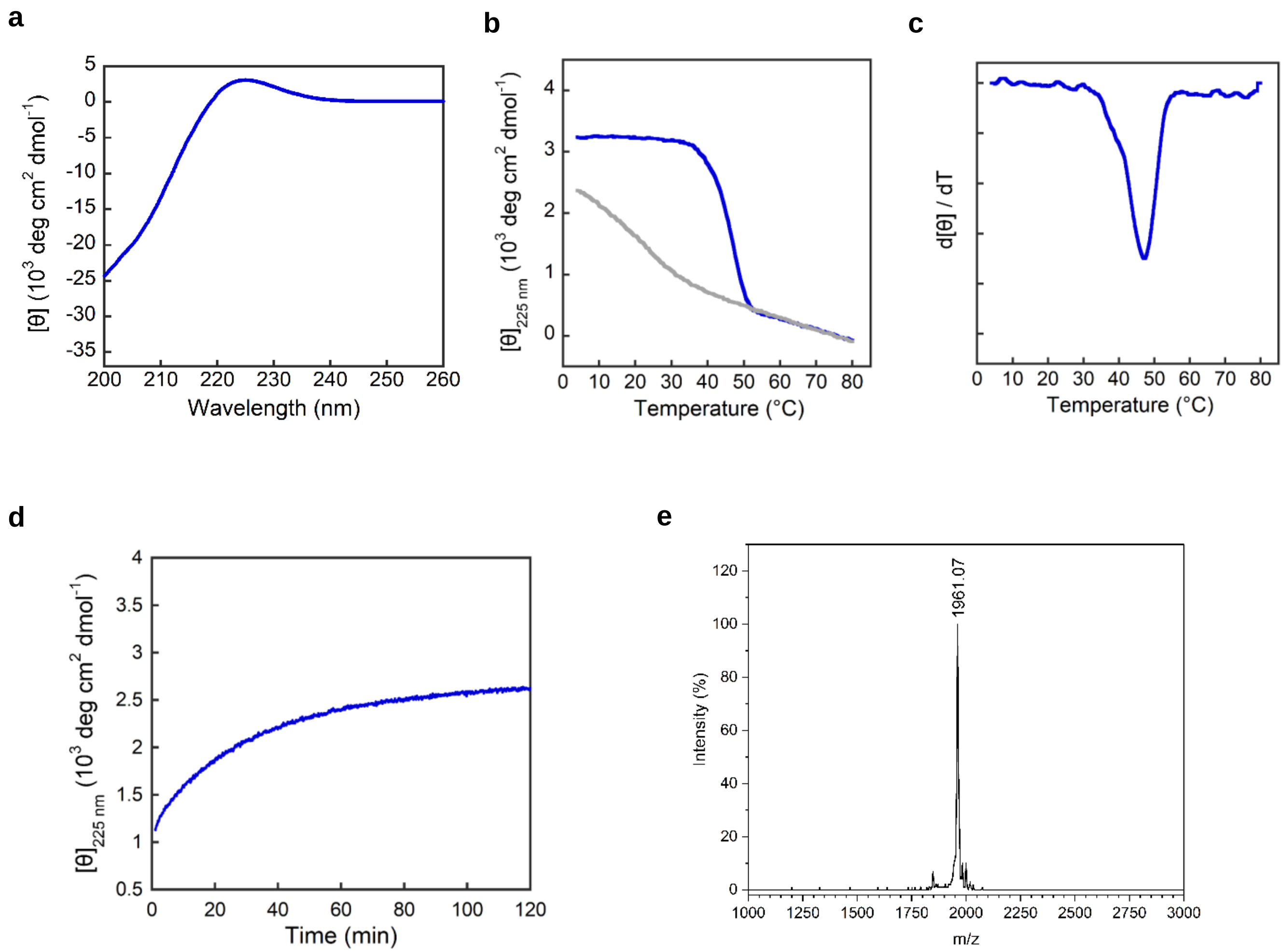

a, The CD spectrum in PBS buffer at 4 °C.

b, The CD thermal unfolding (blue) and cooling (gray) curves in PBS buffer.

c, The first derivative of the thermal unfolding curve, T_m = 47 °C.

d, The CD refolding curve in PBS buffer at 4 °C.

e, MALDI-MS, calculated: 1960.93 $[M+H]^+$, observed: 1961.07 $[M+H]^+$.

Lys-CMP (in 3.5 mM NaOH)

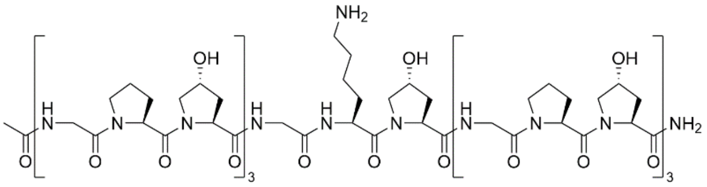

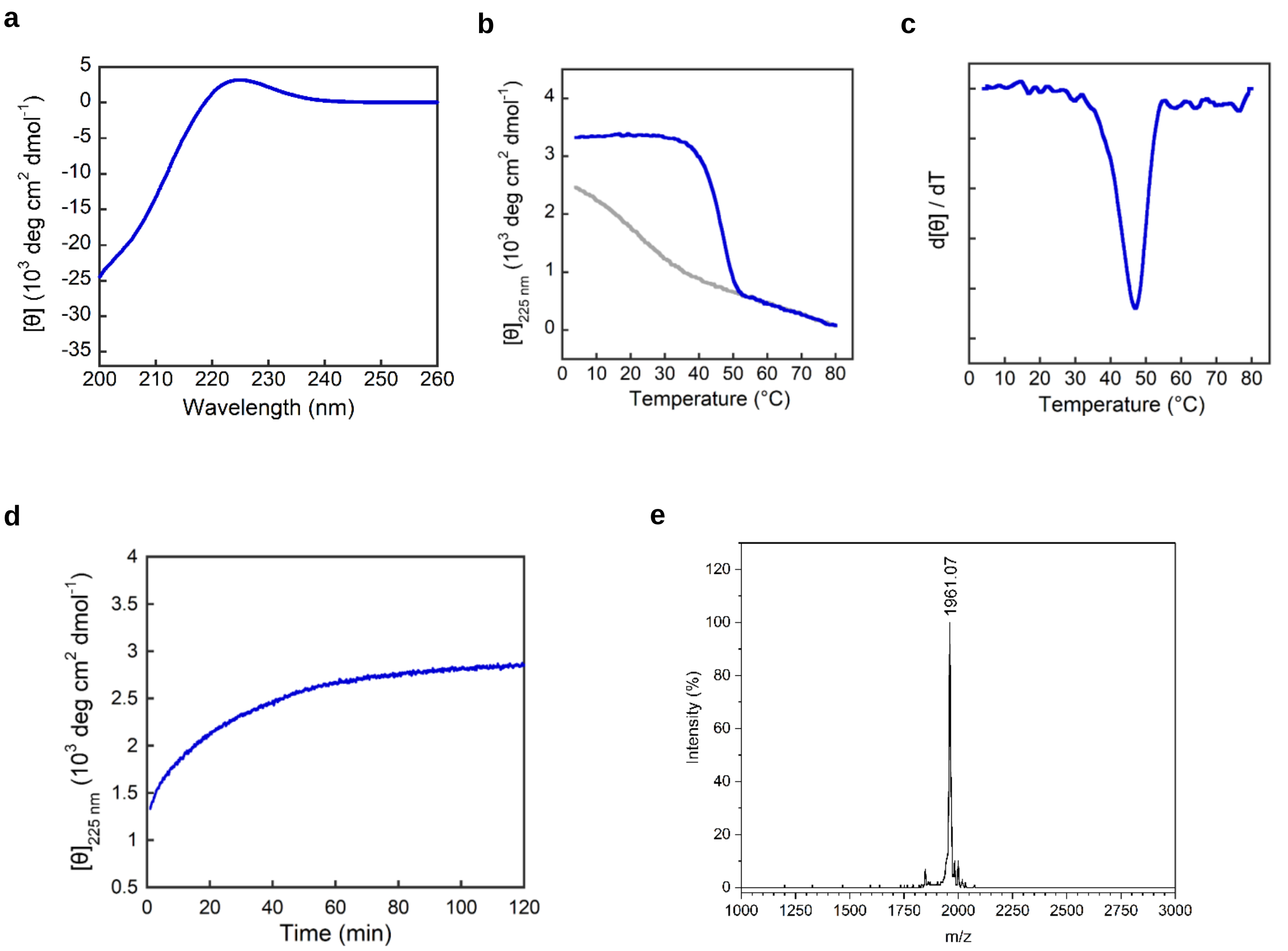

a, The CD spectrum in 3.5 mM NaOH solution (pH 11.5) at 4 °C.

b, The CD thermal unfolding (blue) and cooling (gray) curves in 3.5 mM NaOH solution.

c, The first derivative of the thermal unfolding curve, T_m = 47 °C.

d, The CD refolding curve in 3.5 mM NaOH solution at 4 °C.

e, MALDI-MS, calculated: 1960.93 $[M+H]^+$, observed: 1961.07 $[M+H]^+$.

Sar-CMP (in PBS)

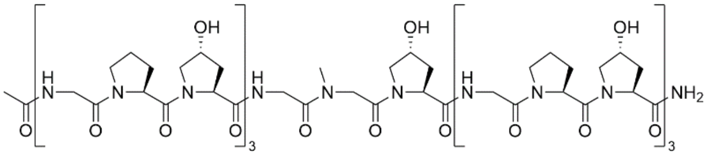

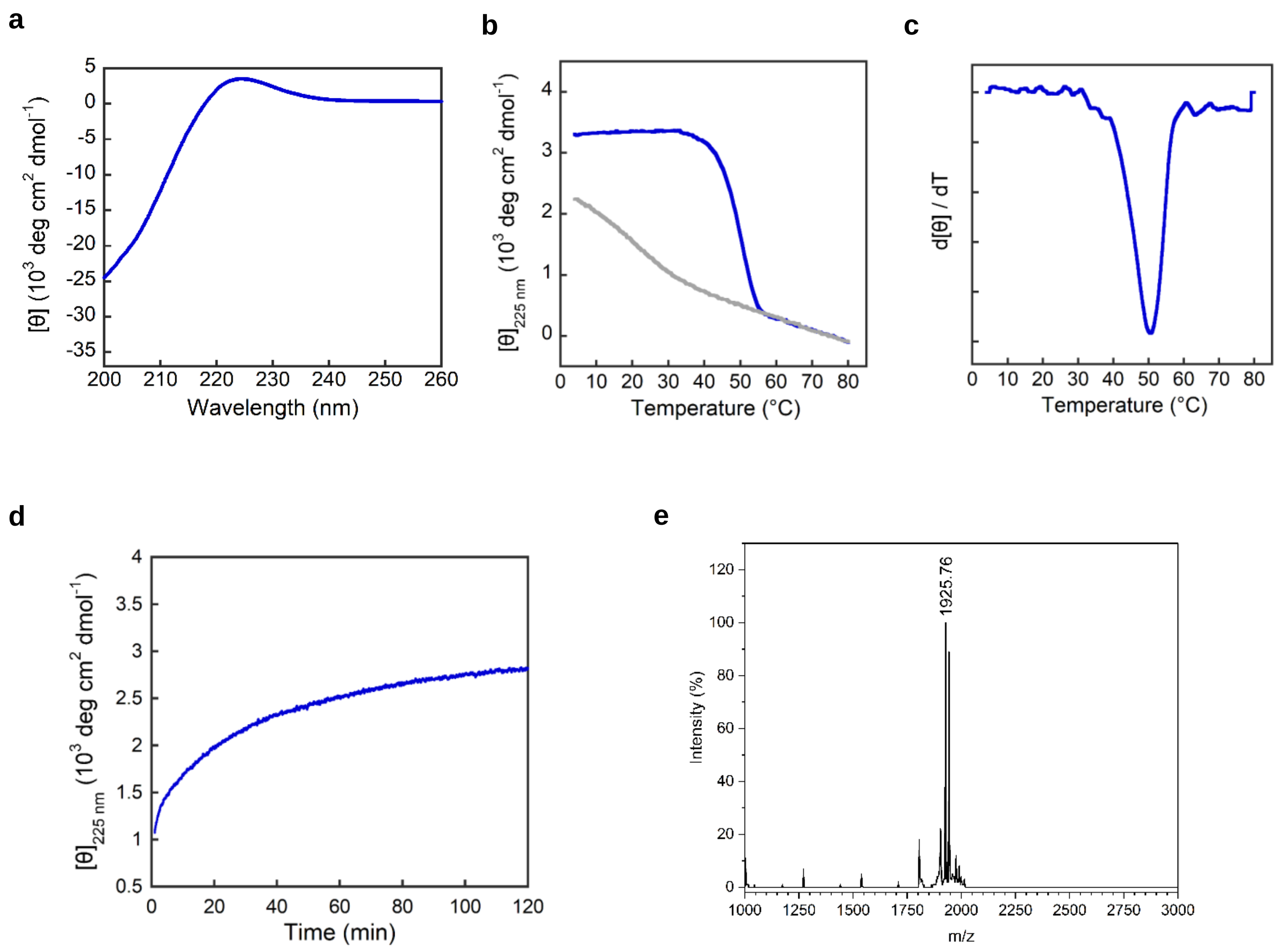

a, The CD spectrum in PBS buffer at 4 °C.

b, The CD thermal unfolding (blue) and cooling (gray) curves in PBS buffer.

c, The first derivative of the thermal unfolding curve, T_m = 51 °C.

d, The CD refolding curve in PBS buffer at 4 °C.

e, MALDI-MS, calculated: 1925.87 $[M+Na]^+$, observed: 1925.76 $[M+Na]^+$.

Nchx-CMP (in PBS)

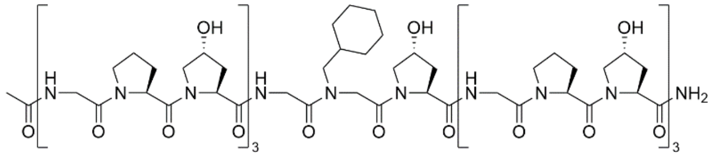

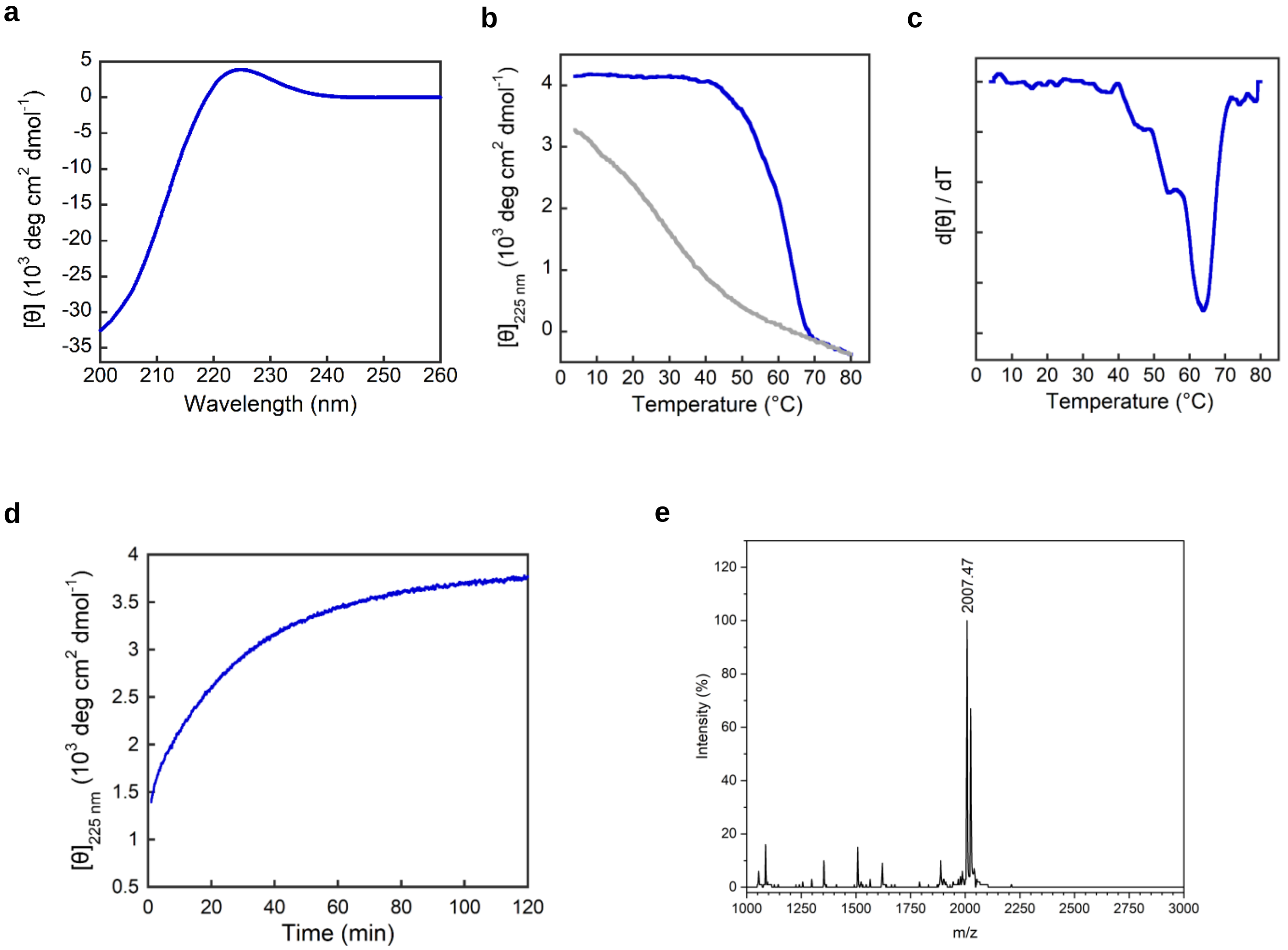

a, The CD spectrum in PBS buffer at 4 °C.

b, The CD thermal unfolding (blue) and cooling (gray) curves in PBS buffer.

c, The first derivative of the thermal unfolding curve, T_m = 64 °C.

d, The CD refolding curve in PBS buffer at 4 °C.

e, MALDI-MS, calculated: 2007.95 $[M+Na]^+$, observed: 2007.47 $[M+Na]^+$.

Nleu-CMP (in PBS)

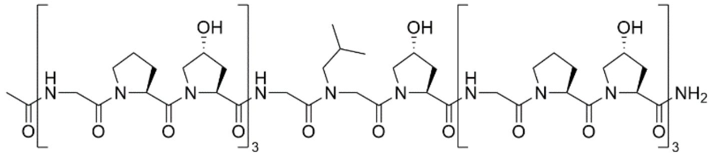

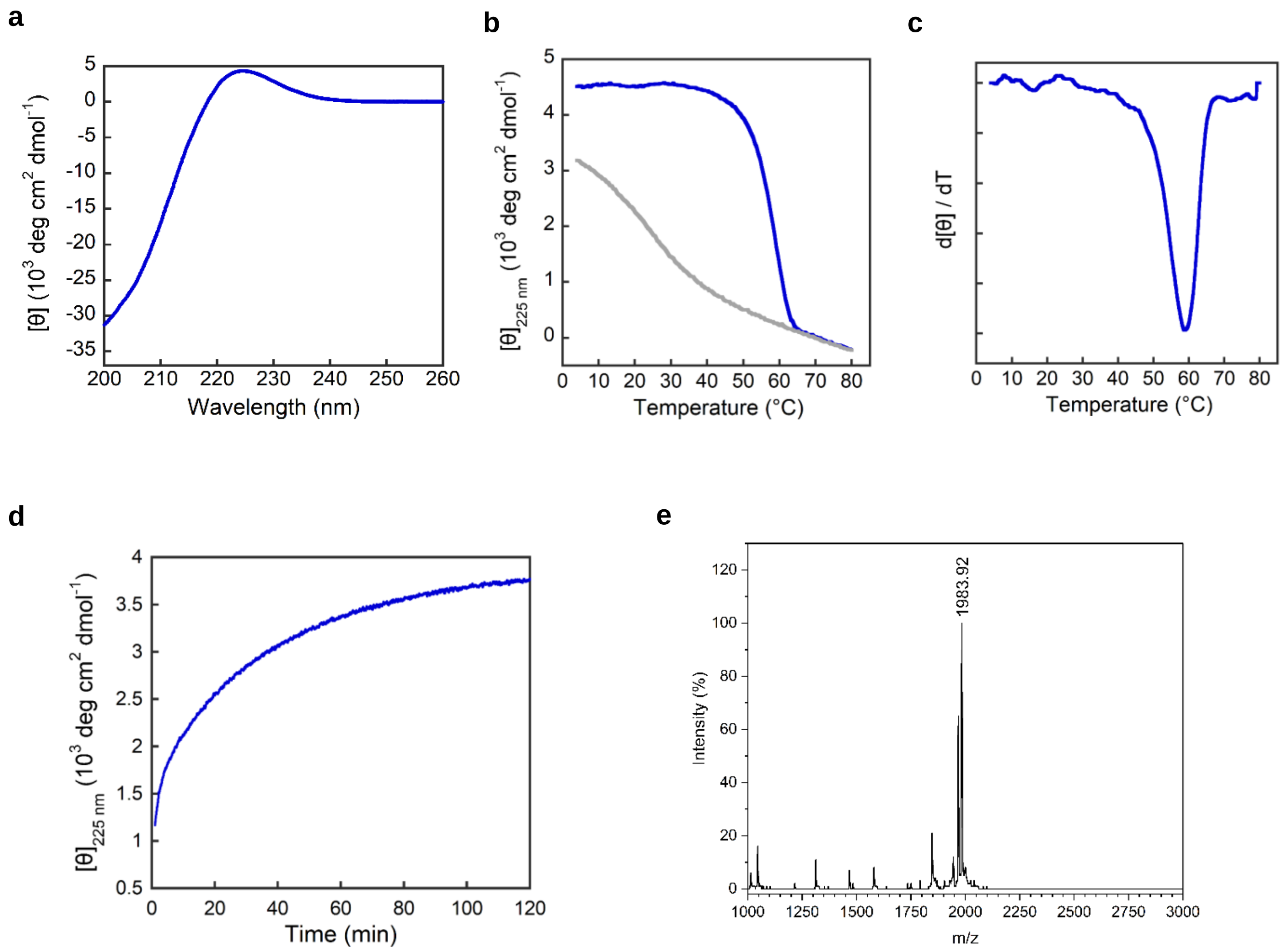

a, The CD spectrum in PBS buffer at 4 °C.

b, The CD thermal unfolding (blue) and cooling (gray) curves in PBS buffer.

c, The first derivative of the thermal unfolding curve, T_m = 59 °C.

d, The CD refolding curve in PBS buffer at 4 °C.

e, MALDI-MS, calculated: 1983.92 $[M+K]^+$, observed: 1983.92 $[M+K]^+$.

Nphe-CMP (in PBS)

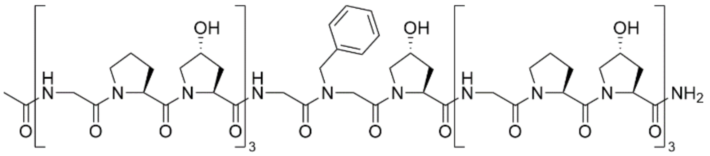

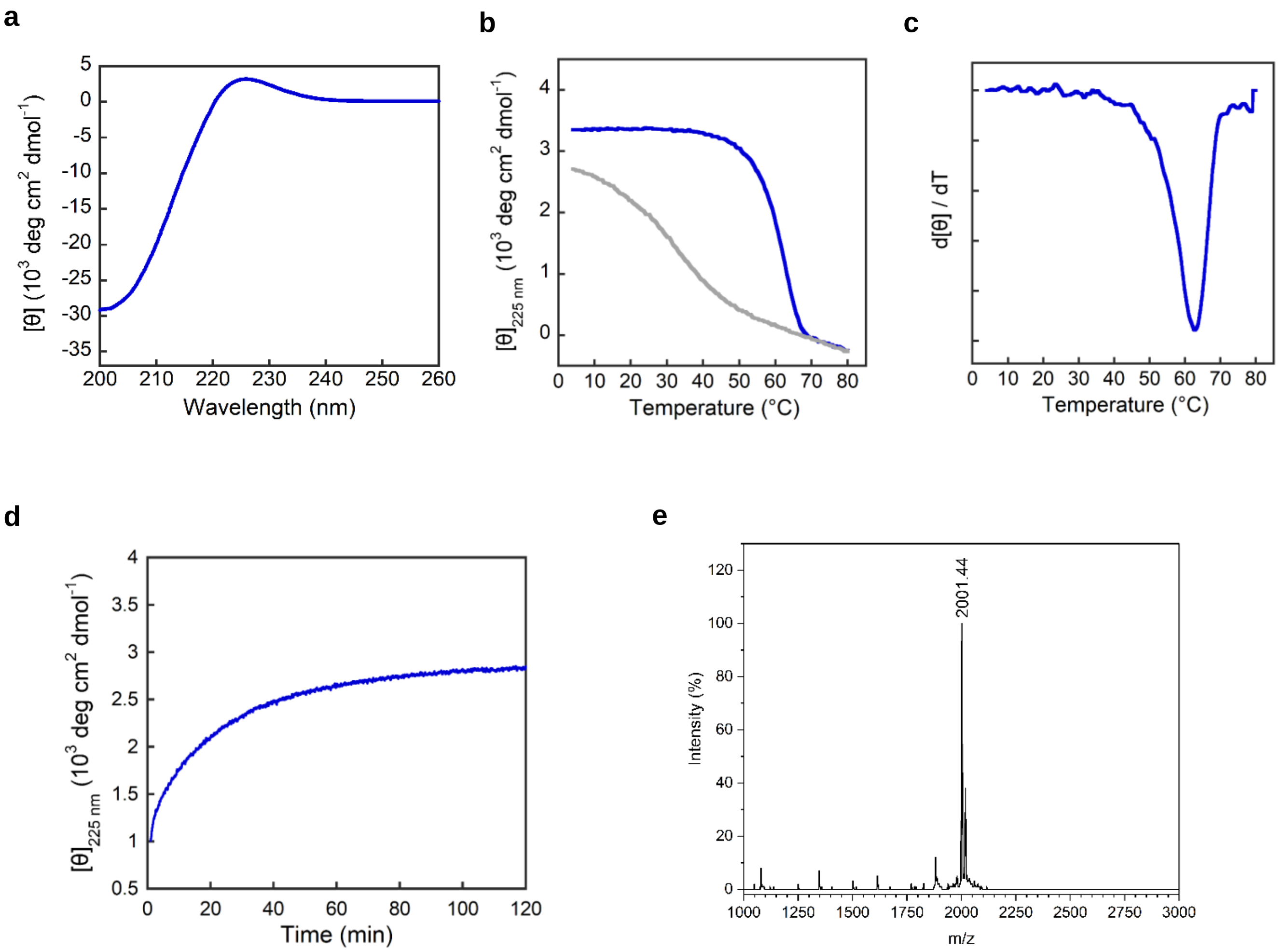

a, The CD spectrum in PBS buffer at 4 °C.

b, The CD thermal unfolding (blue) and cooling (gray) curves in PBS buffer.

c, The first derivative of the thermal unfolding curve, T_m = 61 °C.

d, The CD refolding curve in PBS buffer at 4 °C.

e, MALDI-MS, calculated: 2001.91 $[M+Na]^+$, observed: 2001.44 $[M+Na]^+$.

Nasn-CMP (in PBS)

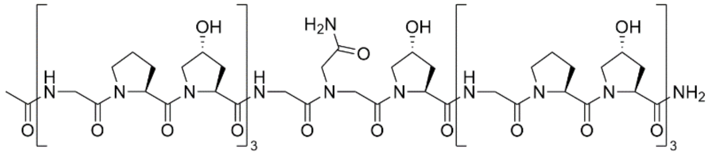

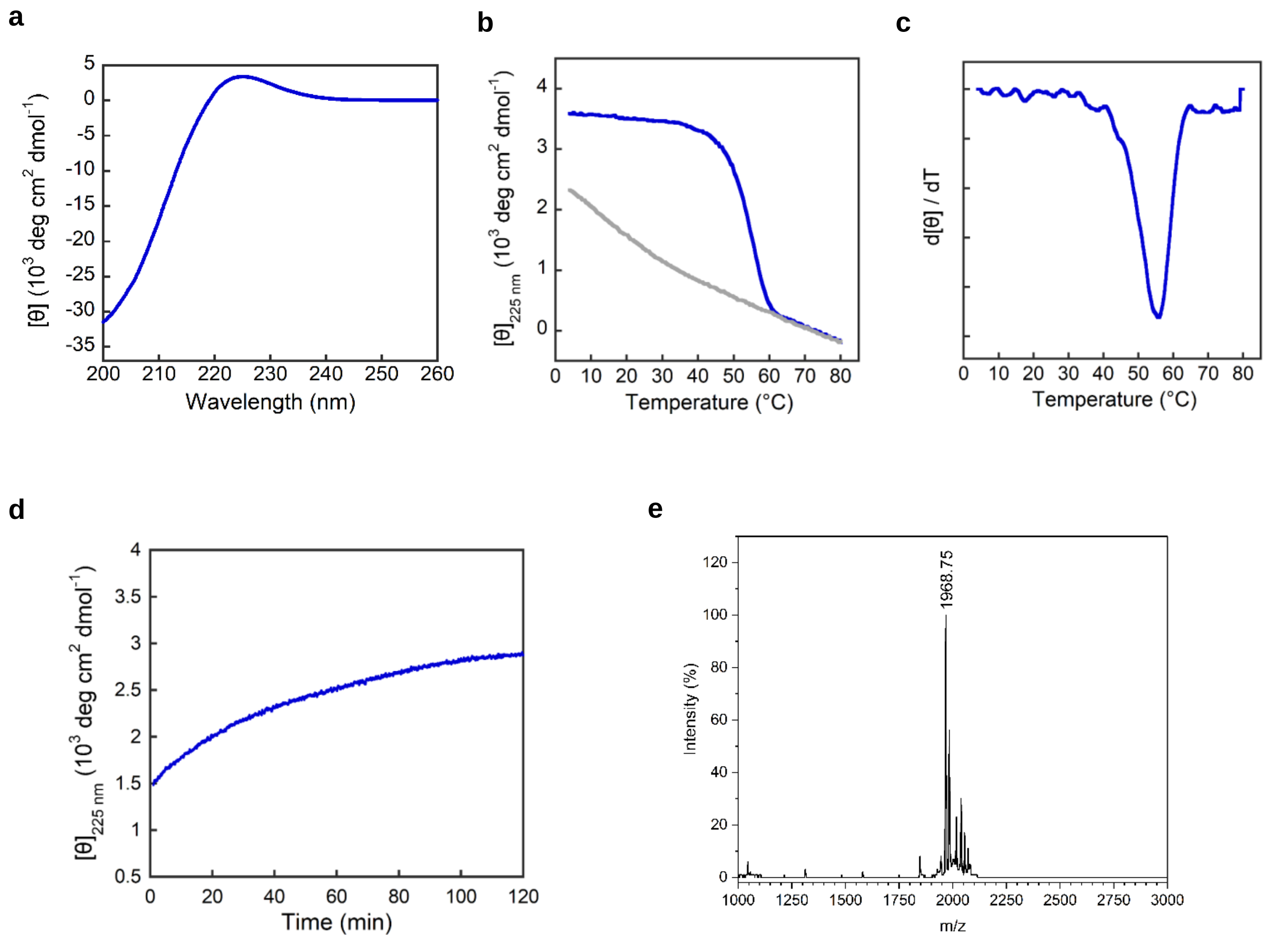

a, The CD spectrum in PBS buffer at 4 °C.

b, The CD thermal unfolding (blue) and cooling (gray) curves in PBS buffer.

c, The first derivative of the thermal unfolding curve, T_m = 56 °C.

d, The CD refolding curve in PBS buffer at 4 °C.

e, MALDI-MS, calculated: 1968.88 $[M+Na]^+$, observed: 1968.75 $[M+Na]^+$.

Nlys-CMP (in PBS)

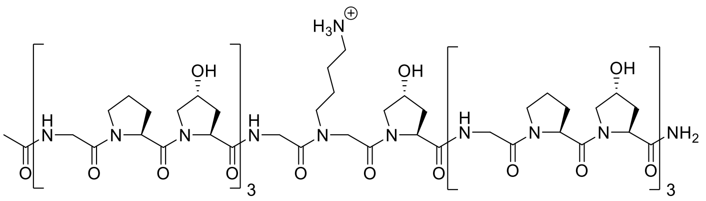

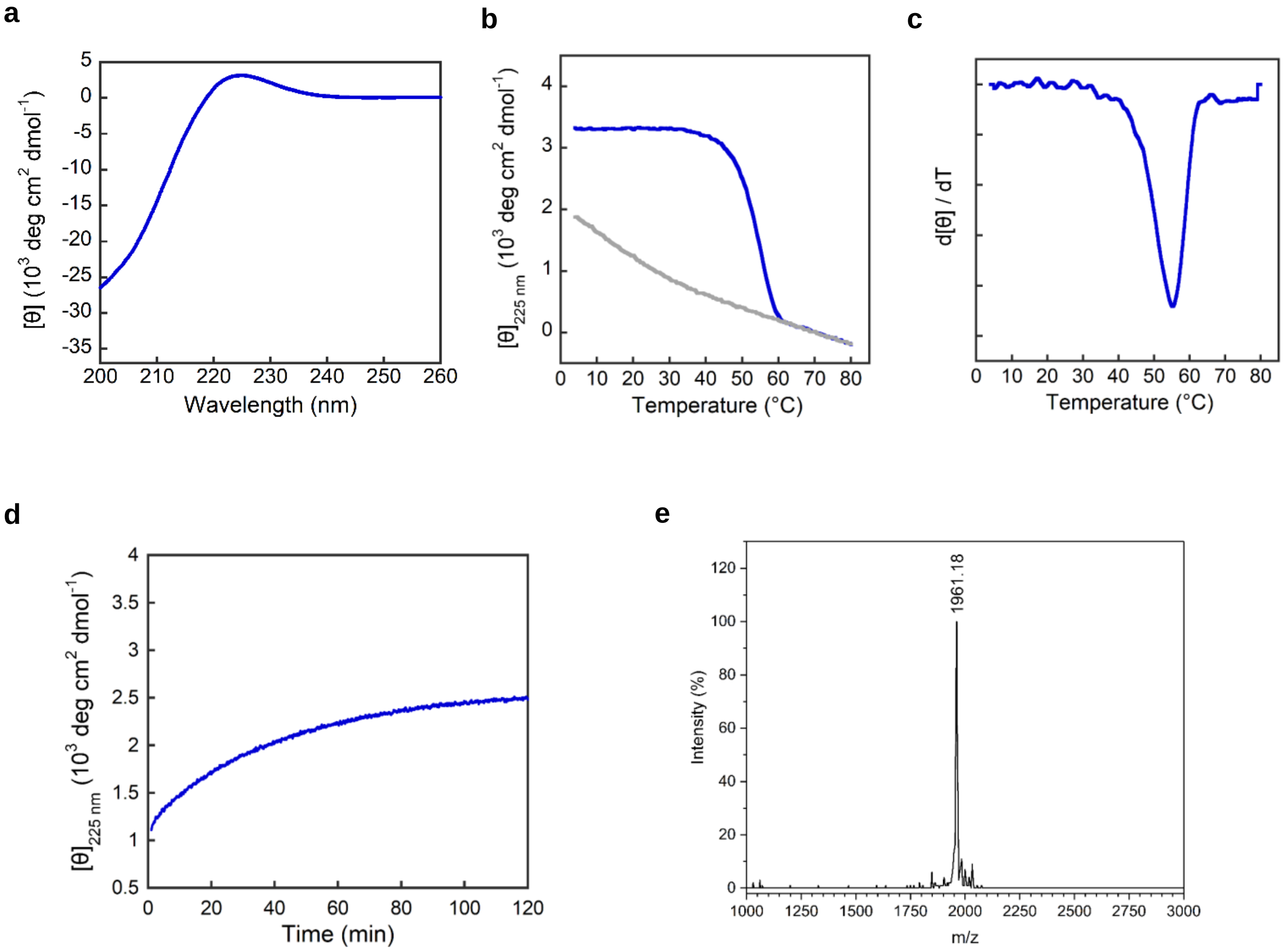

a, The CD spectrum in PBS buffer at 4 °C.

b, The CD thermal unfolding (blue) and cooling (gray) curves in PBS buffer.

c, The first derivative of the thermal unfolding curve, T_m = 55 °C.

d, The CD refolding curve in PBS buffer at 4 °C.

e, MALDI-MS, calculated: 1960.93 $[M+H]^+$, observed: 1961.18 $[M+H]^+$.

Nlys-CMP (in 3.5 mM NaOH)

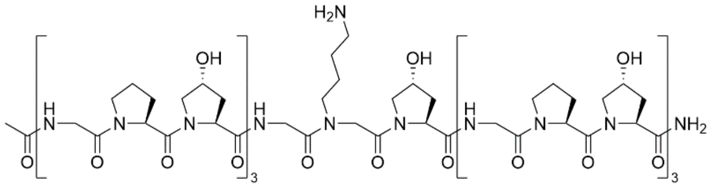

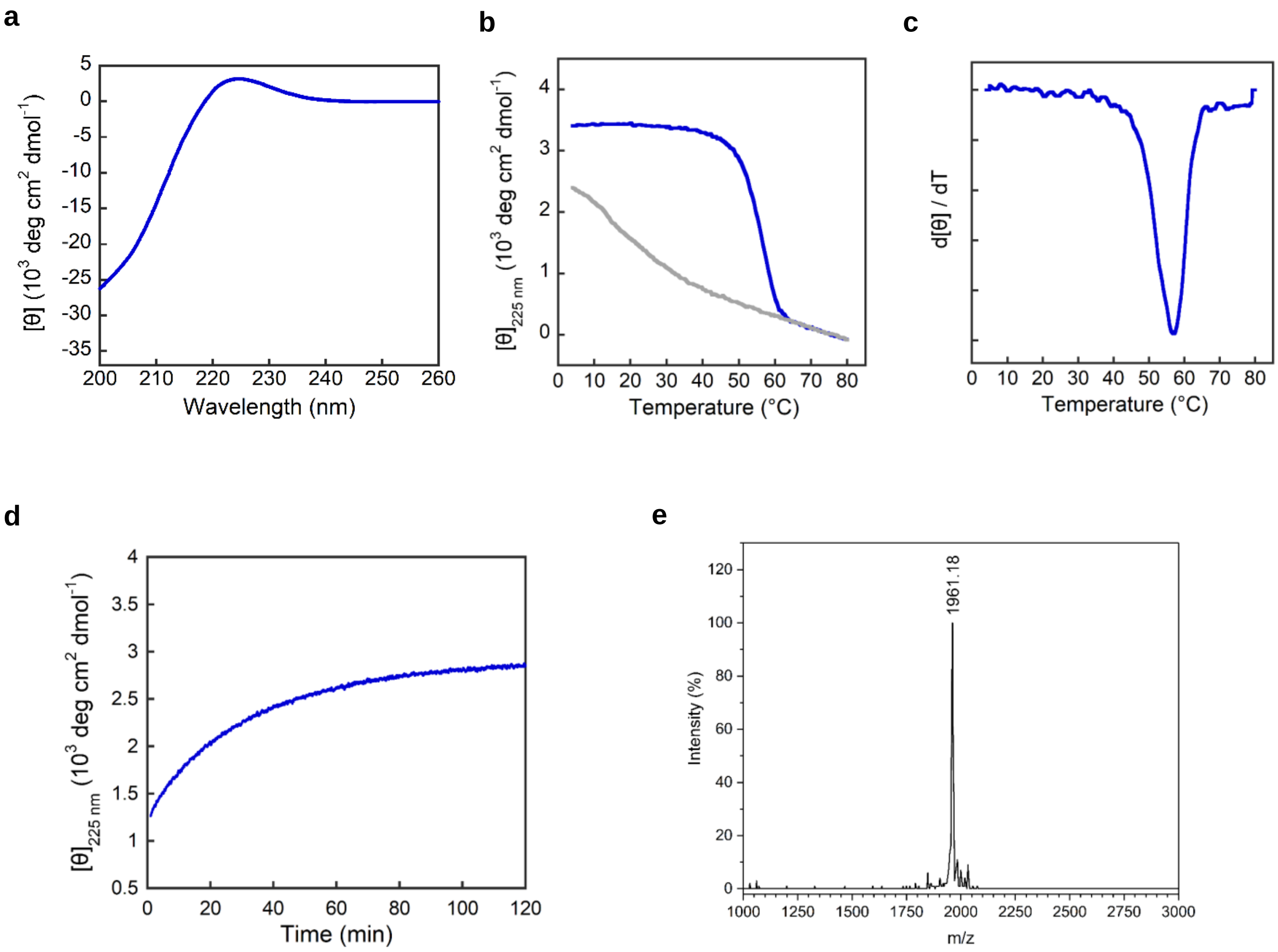

a, The CD spectrum in 3.5 mM NaOH solution (pH 11.5) at 4 °C.

b, The CD thermal unfolding (blue) and cooling (gray) curves in 3.5 mM NaOH solution.

c, The first derivative of the thermal unfolding curve, T_m = 58 °C.

d, The CD refolding curve in 3.5 mM NaOH solution at 4 °C.

e, MALDI-MS, calculated: 1960.93 $[M+H]^+$, observed: 1961.18 $[M+H]^+$.

Nme$_2$ae-CMP (in PBS)

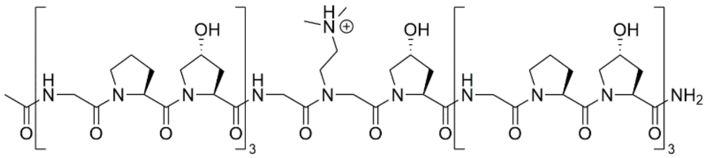

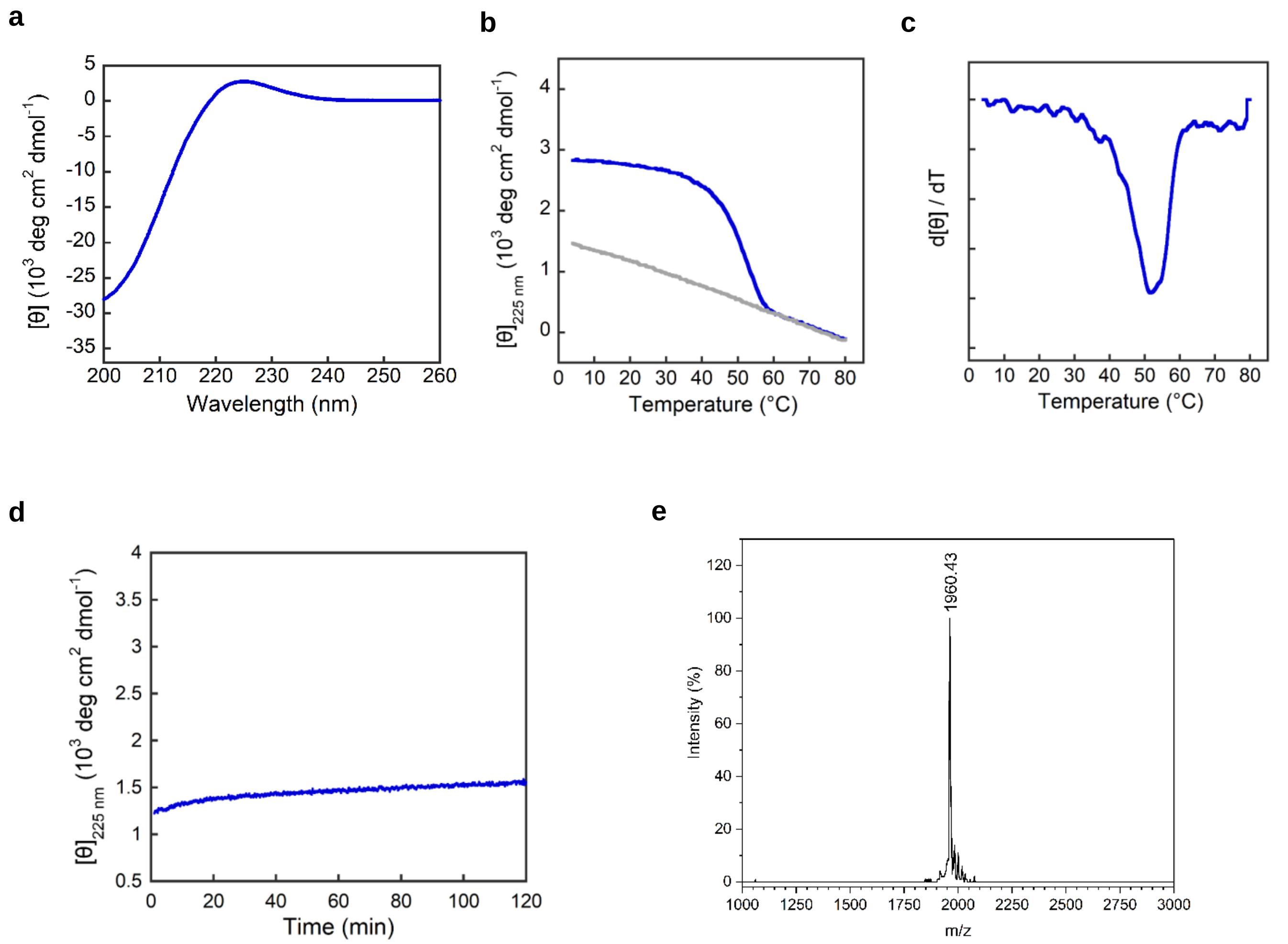

a, The CD spectrum in PBS buffer (pH 7.4) at 4 °C.

b, The CD thermal unfolding (blue) and cooling (gray) curves in PBS buffer.

c, The first derivative of the thermal unfolding curve, T_m = 52 °C.

d, The CD refolding curve in PBS buffer at 4 °C.

e, MALDI-MS, calculated: 1960.93 $[M+H]^+$, observed: 1960.43 $[M+H]^+$.

Nme$_2$ae-CMP (in 3.5 mM NaOH)

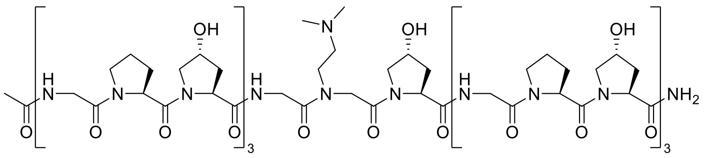

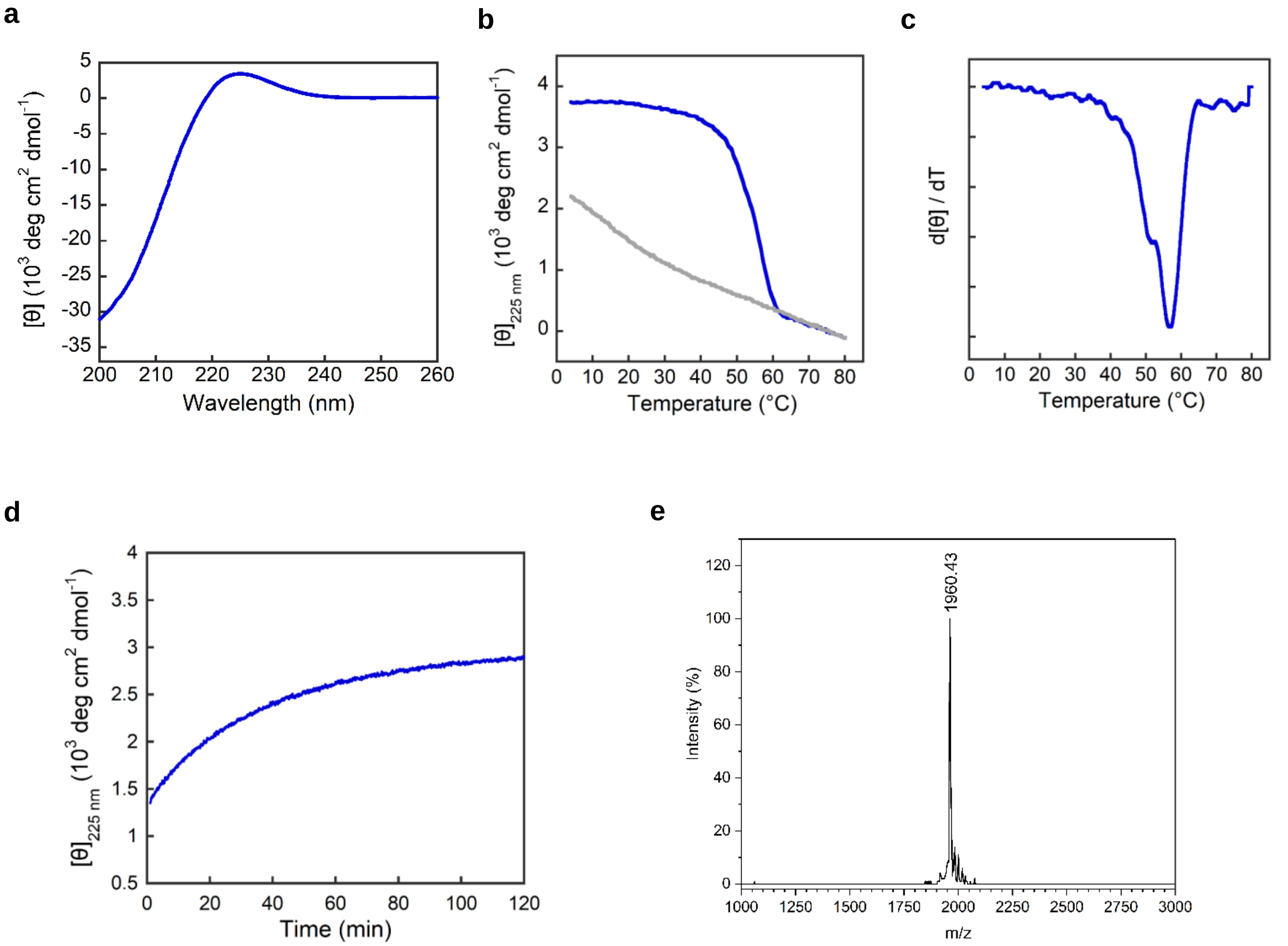

a, The CD spectrum in 3.5 mM NaOH solution (pH 11.5) at 4 °C.

b, The CD thermal unfolding (blue) and cooling (gray) curves in 3.5 mM NaOH solution.

c, The first derivative of the thermal unfolding curve, T_m = 57 °C.

d, The CD refolding curve in 3.5 mM NaOH solution at 4 °C.

e, MALDI-MS, calculated: 1960.93 $[M+H]^+$, observed: 1960.43 $[M+H]^+$.

Nbtm$^+$-CMP (in PBS)

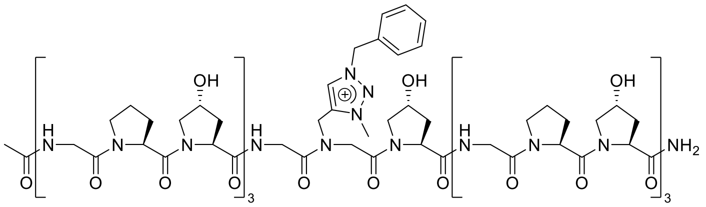

a

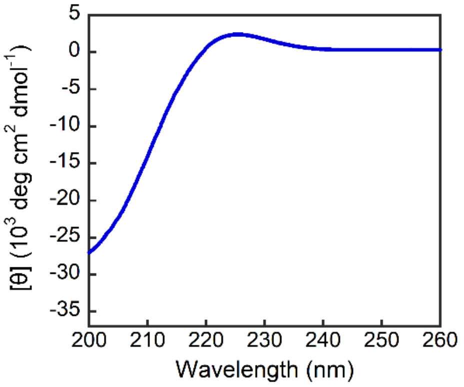

b

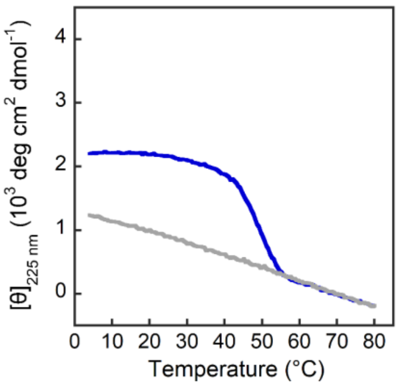

c

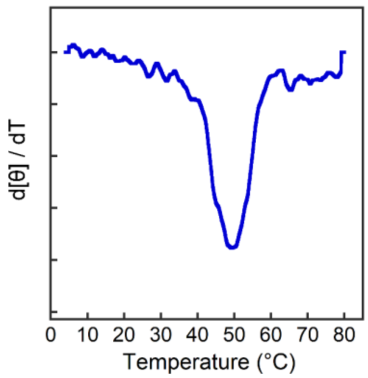

d

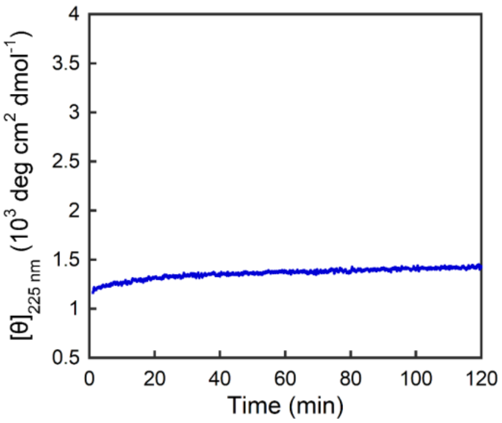

e

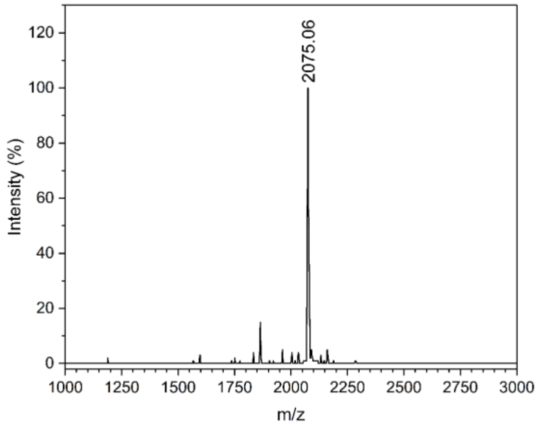

a, The CD spectrum in PBS buffer at 4 °C.

b, The CD thermal unfolding (blue) and cooling (gray) curves in PBS buffer.

c, The first derivative of the thermal unfolding curve, T_m = 49 °C.

d, The CD refolding curve in PBS buffer at 4 °C.

e, MALDI-MS, calculated: 2075.96 $[M+H]^+$, observed: 2075.06 $[M+H]^+$.

Nme$_3$ae-CMP (in PBS)

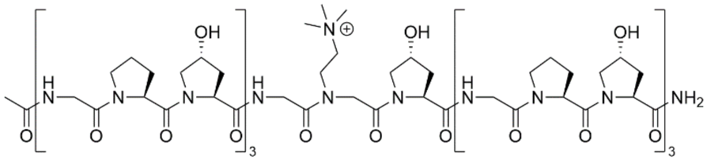

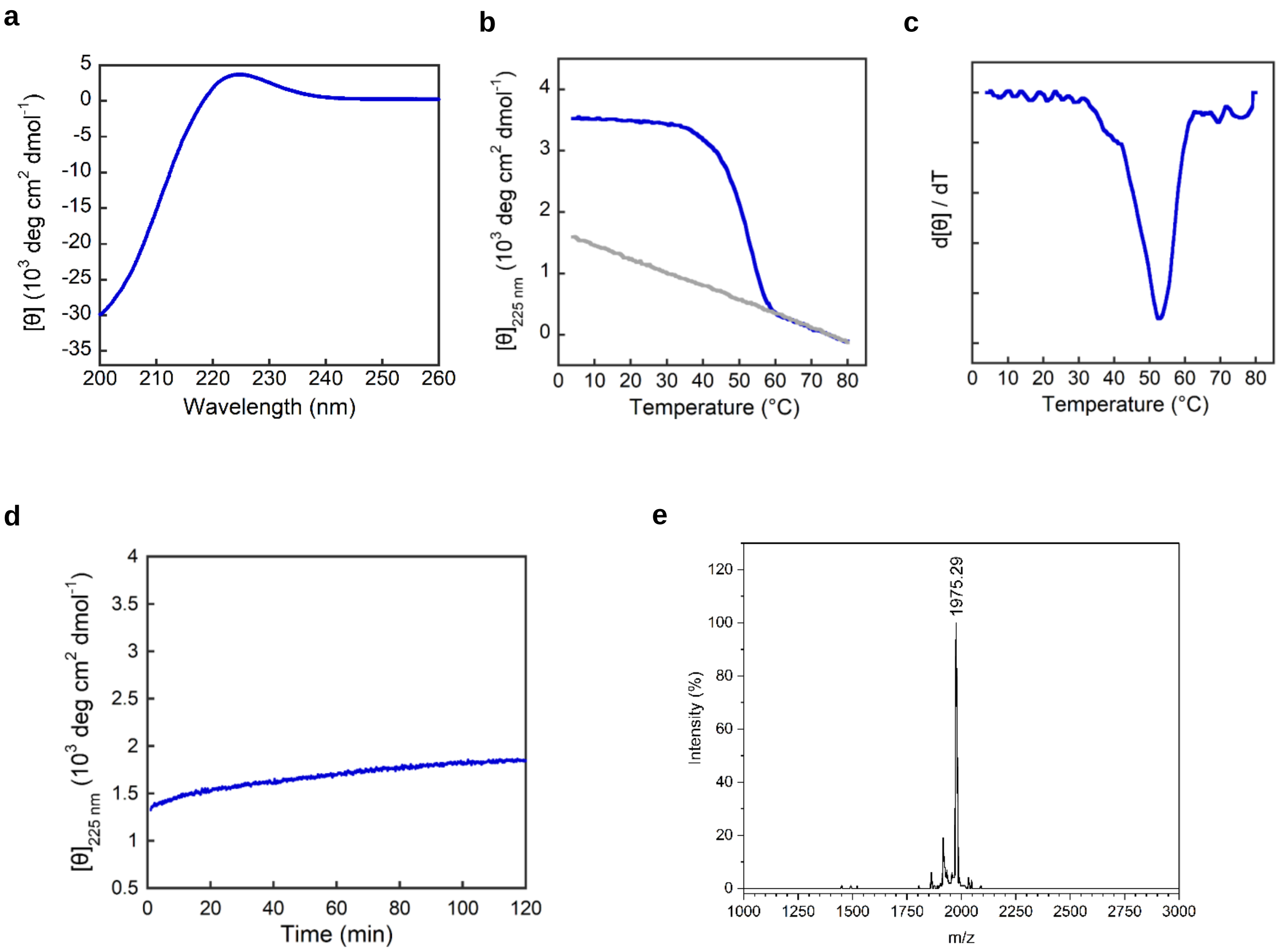

a, The CD spectrum in PBS buffer at 4 °C.

b, The CD thermal unfolding (blue) and cooling (gray) curves in PBS buffer.

c, The first derivative of the thermal unfolding curve, T_m = 53 °C.

d, The CD refolding curve in PBS buffer at 4 °C.

e, MALDI-MS, calculated: 1975.96 $[M+H]^+$, observed: 1975.29 $[M+H]^+$.

Net_2ae-CMP (in PBS)

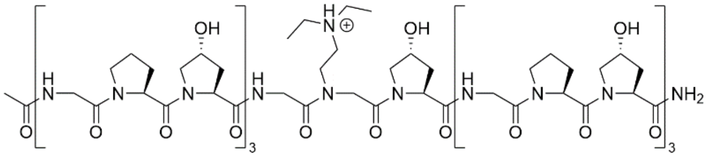

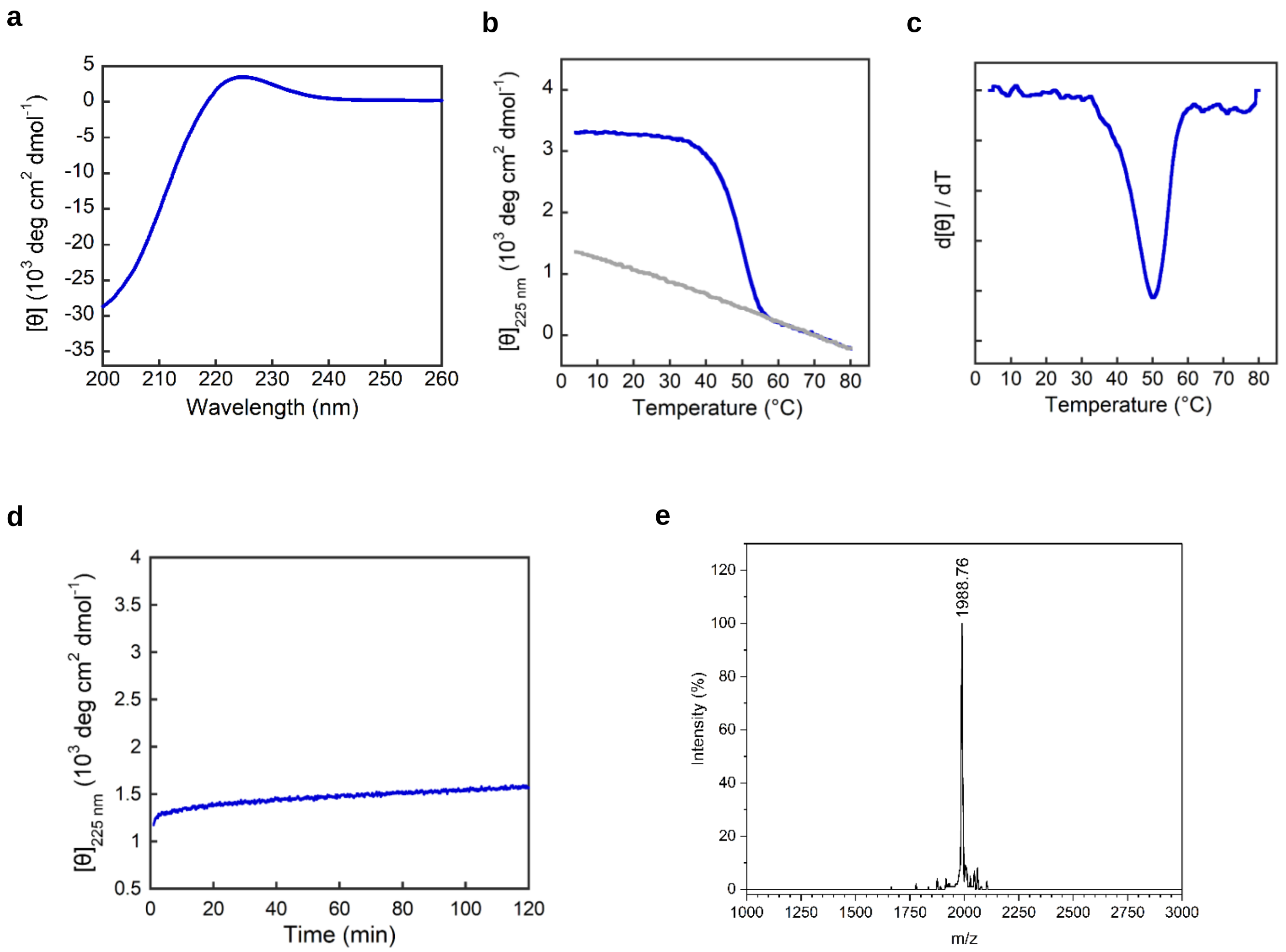

a, The CD spectrum in PBS buffer (pH 7.4) at 4 °C.

b, The CD thermal unfolding (blue) and cooling (gray) curves in PBS buffer.

c, The first derivative of the thermal unfolding curve, T_m = 50 °C.

d, The CD refolding curve in PBS buffer at 4 °C.

e, MALDI-MS, calculated: 1988.96 $[M+H]^+$, observed: 1988.76 $[M+H]^+$.

Net$_2$ae-CMP (in 3.5 mM NaOH)

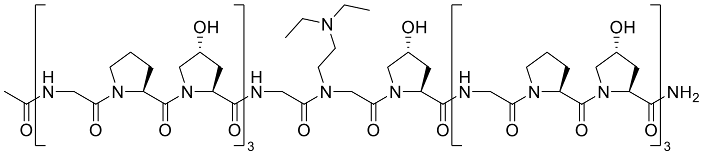

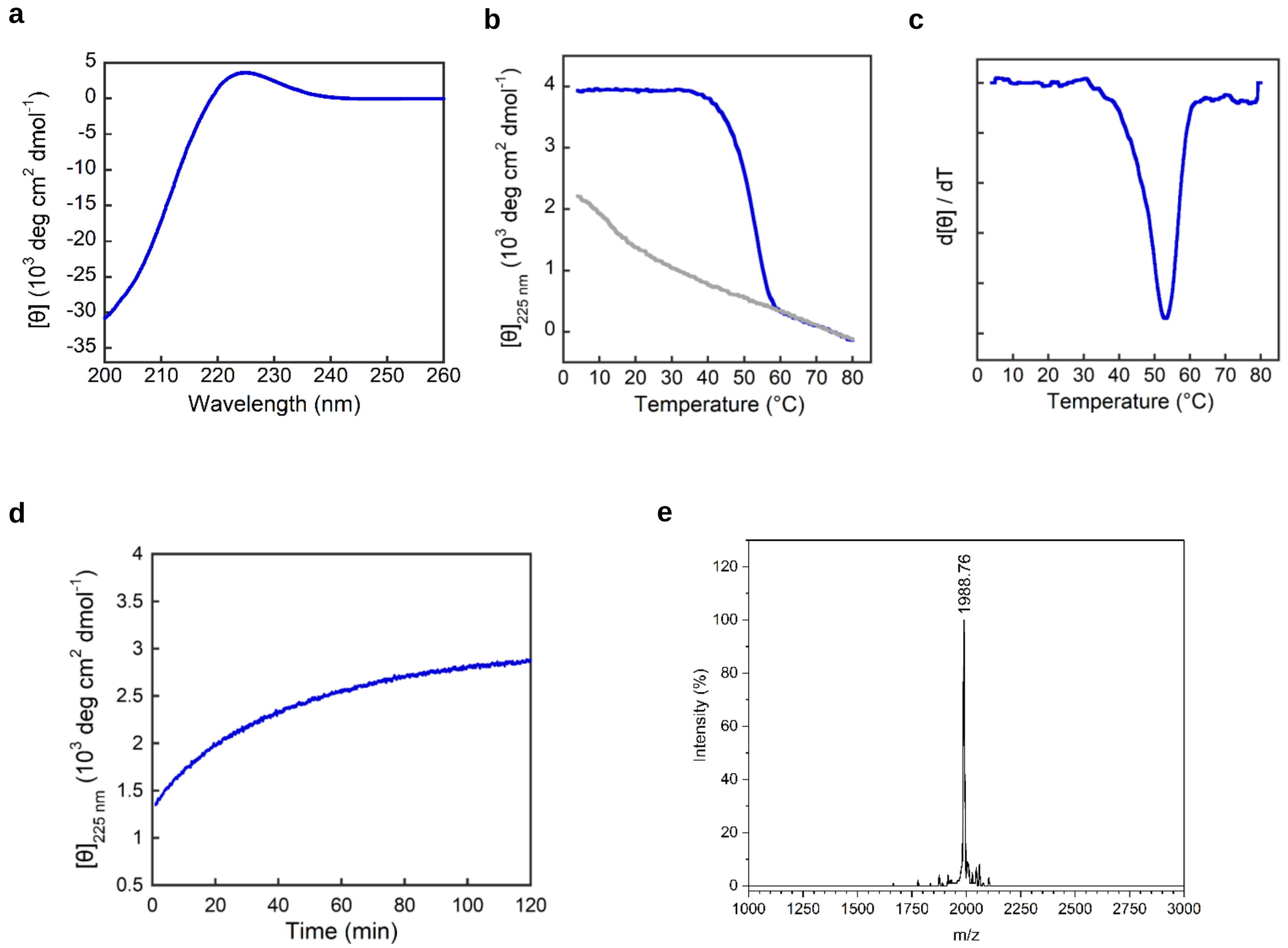

a, The CD spectrum in 3.5 mM NaOH solution (pH 11.5) at 4 °C.

b, The CD thermal unfolding (blue) and cooling (gray) curves in 3.5 mM NaOH solution.

c, The first derivative of the thermal unfolding curve, T_m = 53 °C.

d, The CD refolding curve in 3.5 mM NaOH solution at 4 °C.

e, MALDI-MS, calculated: 1988.96 $[M+H]^+$, observed: 1988.76 $[M+H]^+$.

N^{i}pr$_2$ae-CMP (in PBS)

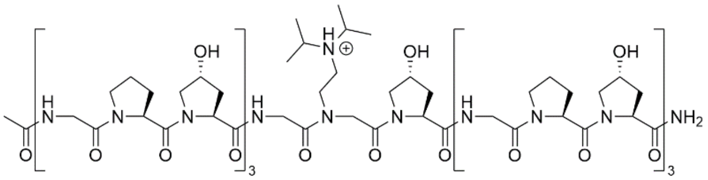

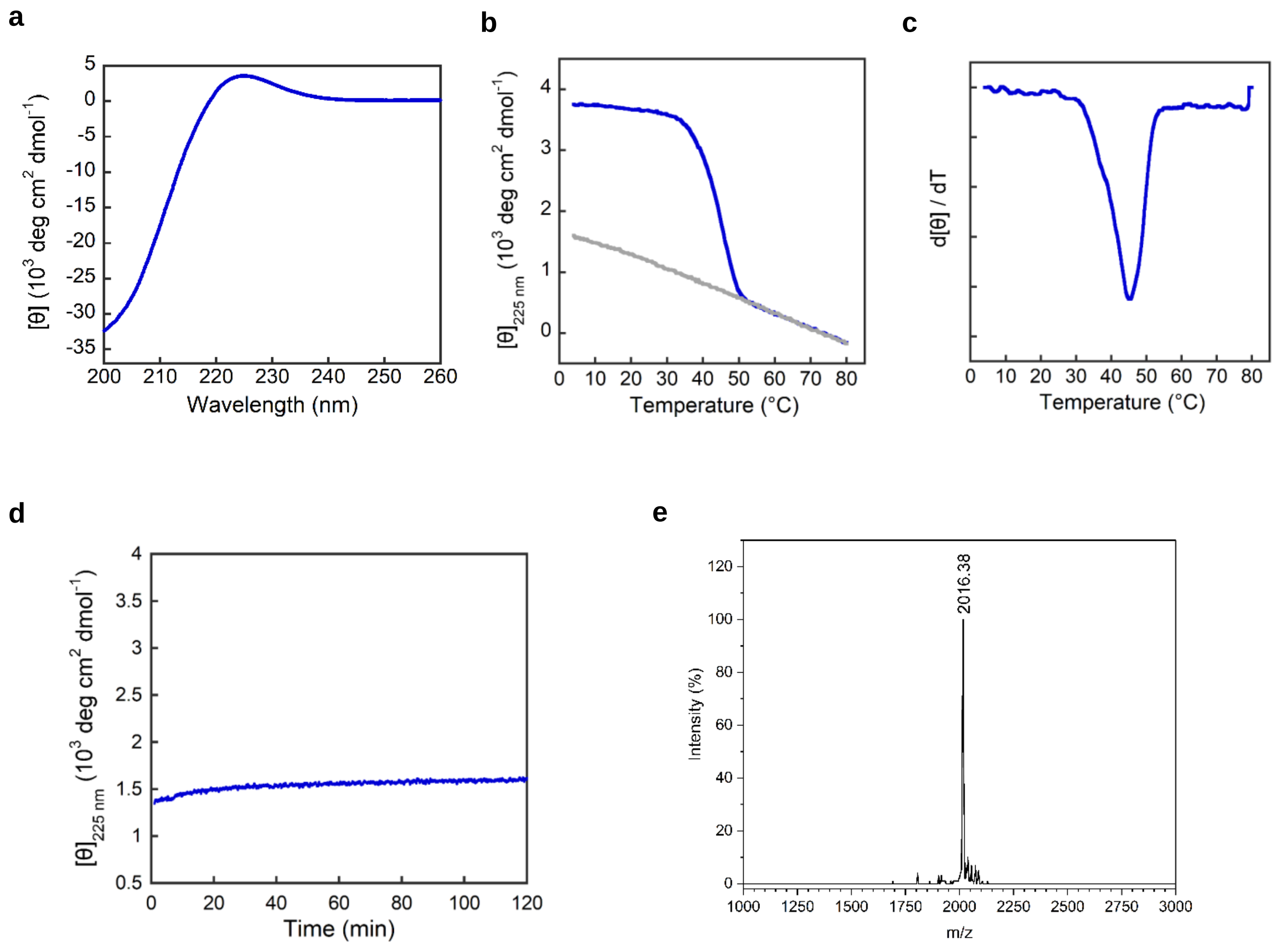

a, The CD spectrum in PBS buffer (pH 7.4) at 4 °C.

b, The CD thermal unfolding (blue) and cooling (gray) curves in PBS buffer.

c, The first derivative of the thermal unfolding curve, T_m = 45 °C.

d, The CD refolding curve in PBS buffer at 4 °C.

e, MALDI-MS, calculated: 2017.00 [M+H]$^+$, observed: 2016.38 [M+H]$^+$.

N^{i}pr$_2$ae-CMP (in 3.5 mM NaOH)

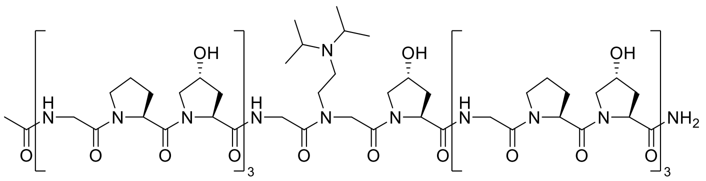

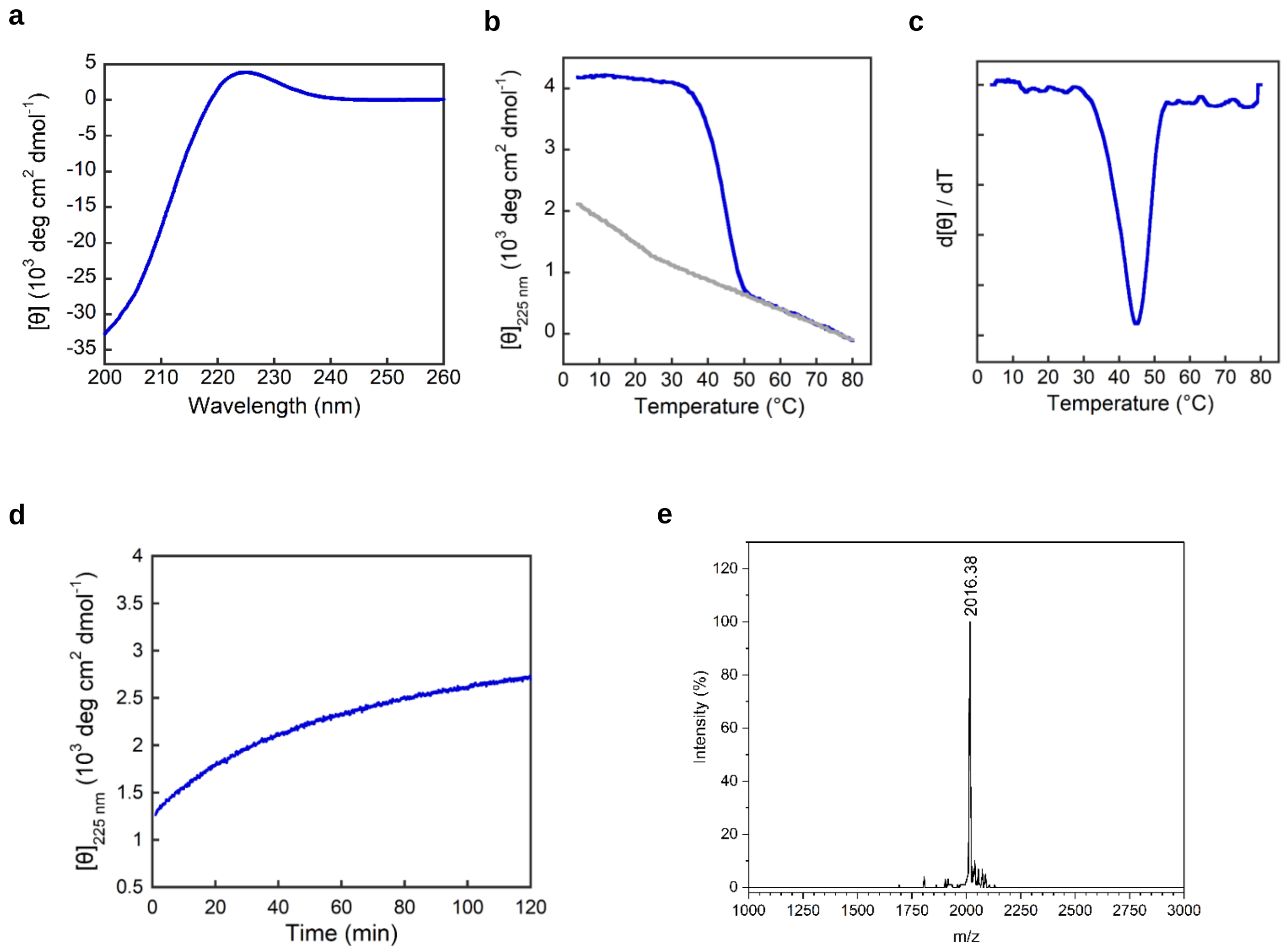

a, The CD spectrum in 3.5 mM NaOH solution (pH 11.5) at 4 °C.

b, The CD thermal unfolding (blue) and cooling (gray) curves in 3.5 mM NaOH solution.

c, The first derivative of the thermal unfolding curve, T_m = 45 °C.

d, The CD refolding curve in 3.5 mM NaOH solution at 4 °C.

e, MALDI-MS, calculated: 2017.00 [M+H]$^+$, observed: 2016.38 [M+H]$^+$.

N2pic-CMP (in PBS)

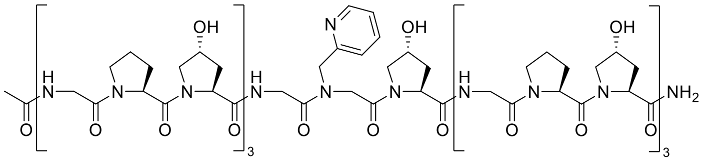

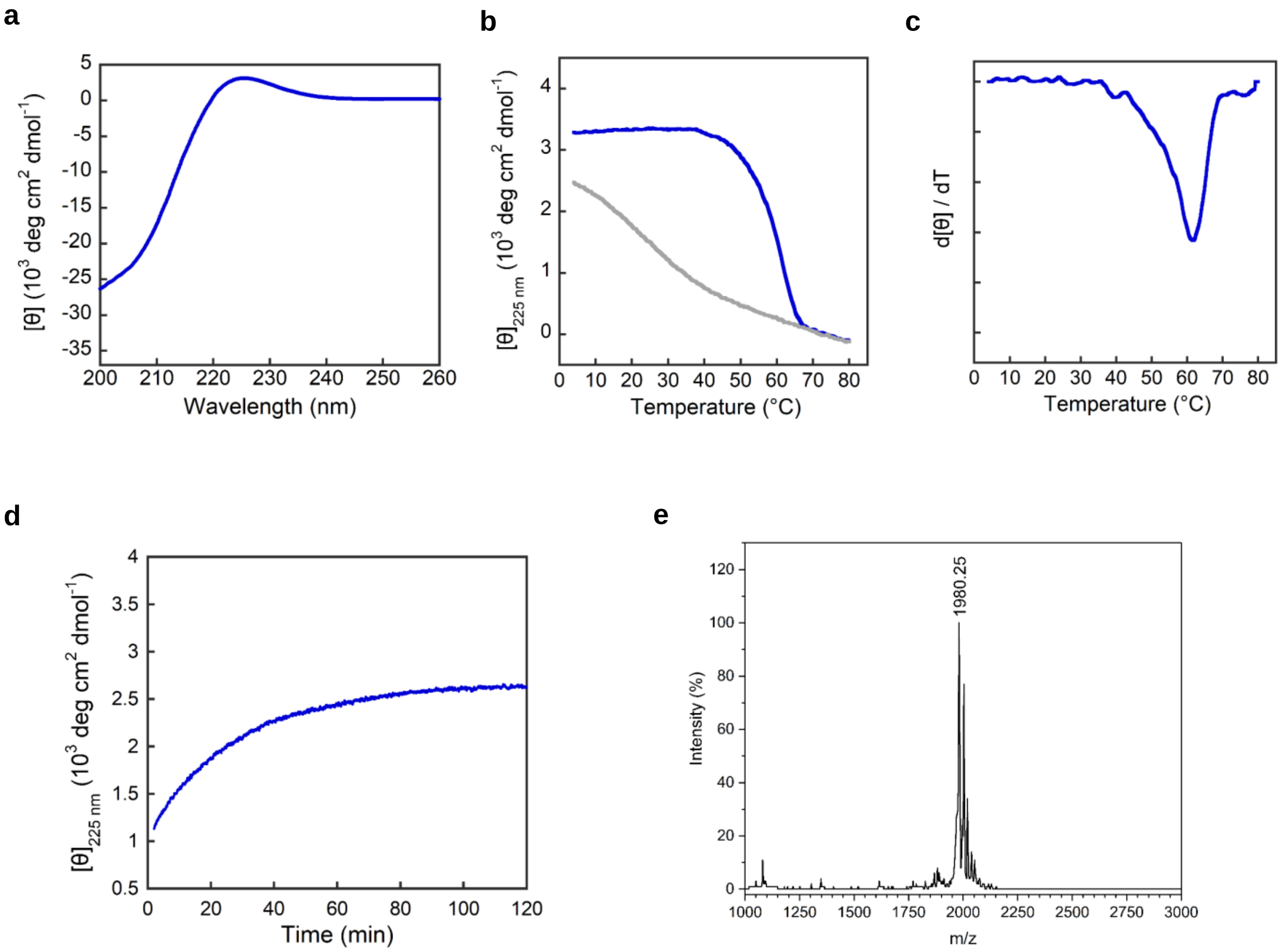

a, The CD spectrum in PBS solution (pH 7.4) at 4 °C.

b, The CD thermal unfolding (blue) and cooling (gray) curves in PBS solution.

c, The first derivative of the thermal unfolding curve, T_m = 62 °C.

d, The CD refolding curve in PBS solution at 4 °C.

e, MALDI-MS, calculated: 1980.90 $[M+H]^+$, observed: 1980.25 $[M+H]^+$.

N2pic-CMP (in 1 mM HCl)

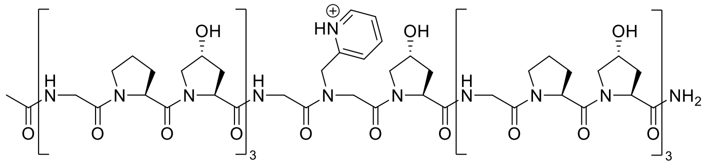

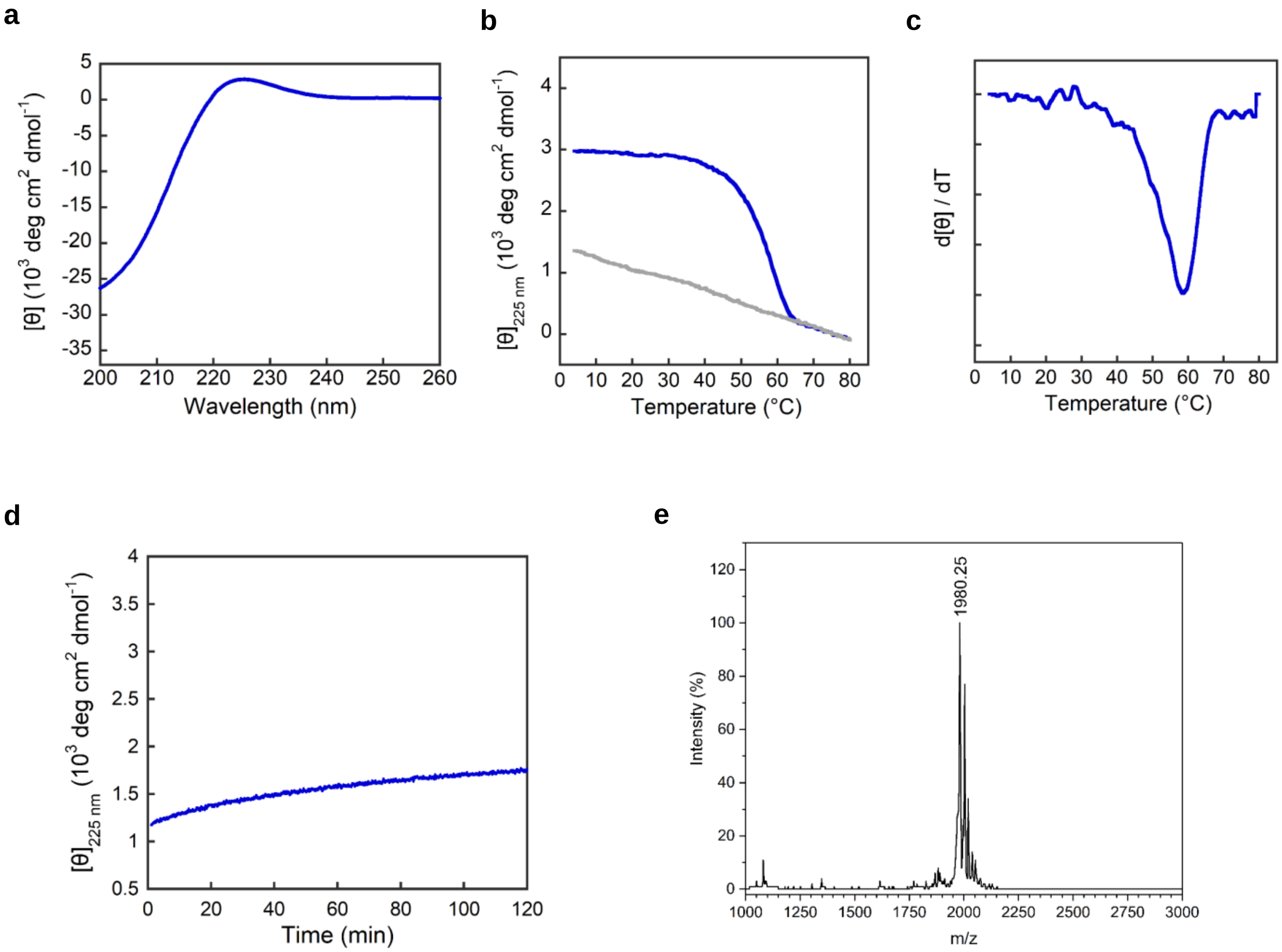

a, The CD spectrum in 1 mM HCl solution (pH 3.0) at 4 °C.

b, The CD thermal unfolding (blue) and cooling (gray) curves in 1 mM HCl solution.

c, The first derivative of the thermal unfolding curve, T_m = 59 °C.

d, The CD refolding curve in 1 mM HCl solution at 4 °C.

e, MALDI-MS, calculated: 1980.90 $[M+H]^+$, observed: 1980.25 $[M+H]^+$.

N2pic2-CMP (in PBS)

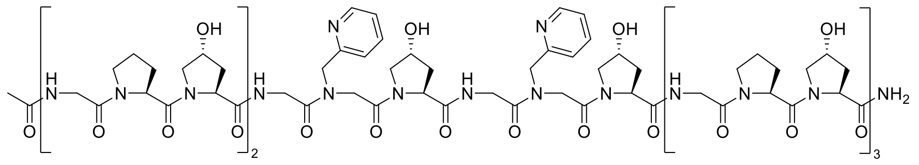

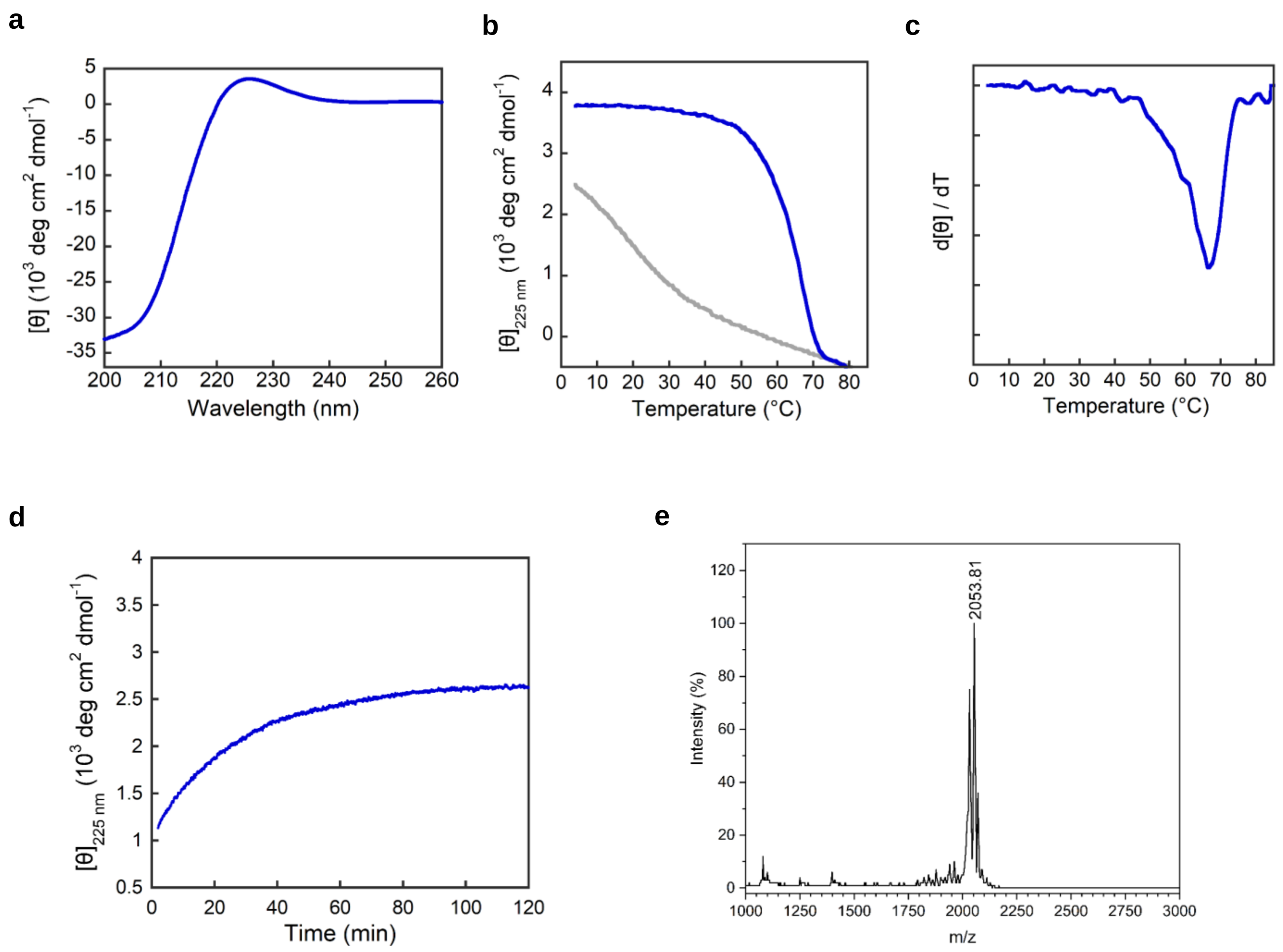

a, The CD spectrum in PBS solution (pH 7.4) at 4 °C.

b, The CD thermal unfolding (blue) and cooling (gray) curves in PBS solution.

c, The first derivative of the thermal unfolding curve, T_m = 67 °C.

d, The CD refolding curve in PBS solution at 4 °C.

e, MALDI-MS, calculated: 2053.91 $[M+Na]^+$, observed: 2053.81 $[M+Na]^+$.

N2pic2-CMP (in 1 mM HCl)

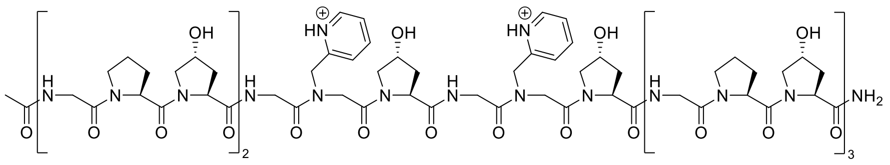

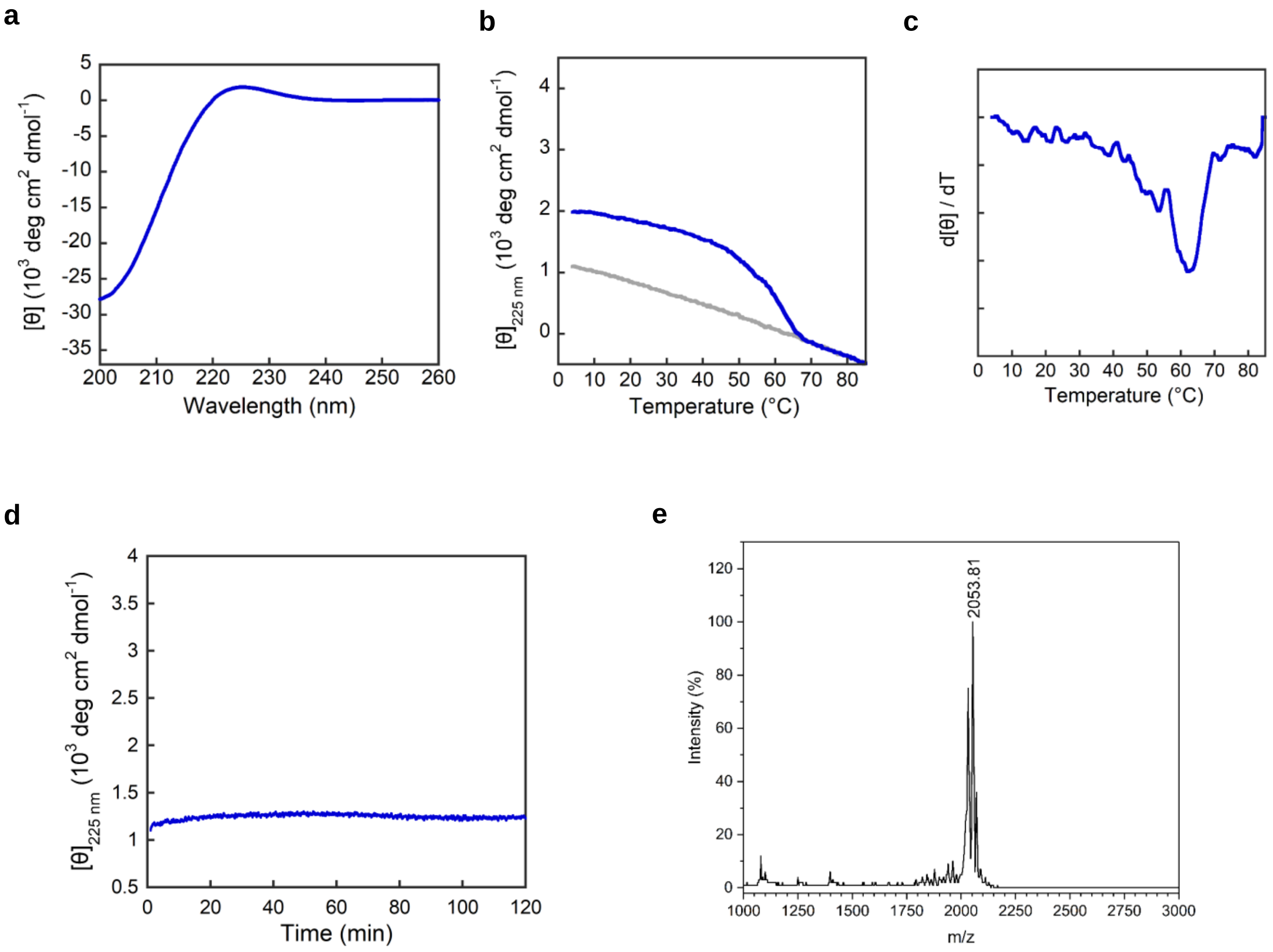

a, The CD spectrum in 1 mM HCl solution (pH 3.0) at 4 °C.

b, The CD thermal unfolding (blue) and cooling (gray) curves in 1 mM HCl solution.

c, The first derivative of the thermal unfolding curve, T_m = 63 °C.

d, The CD refolding curve in 1 mM HCl solution at 4 °C.

e, MALDI-MS, calculated: 2053.91 $[M+Na]^+$, observed: 2053.81 $[M+Na]^+$.

N2pic3-CMP (in PBS)

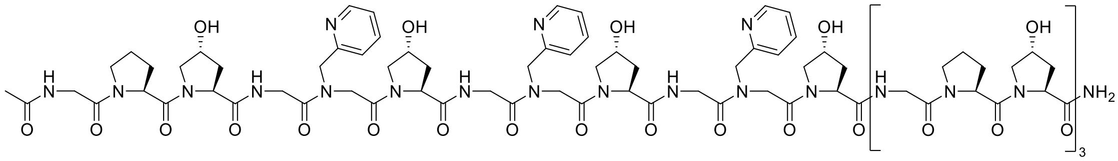

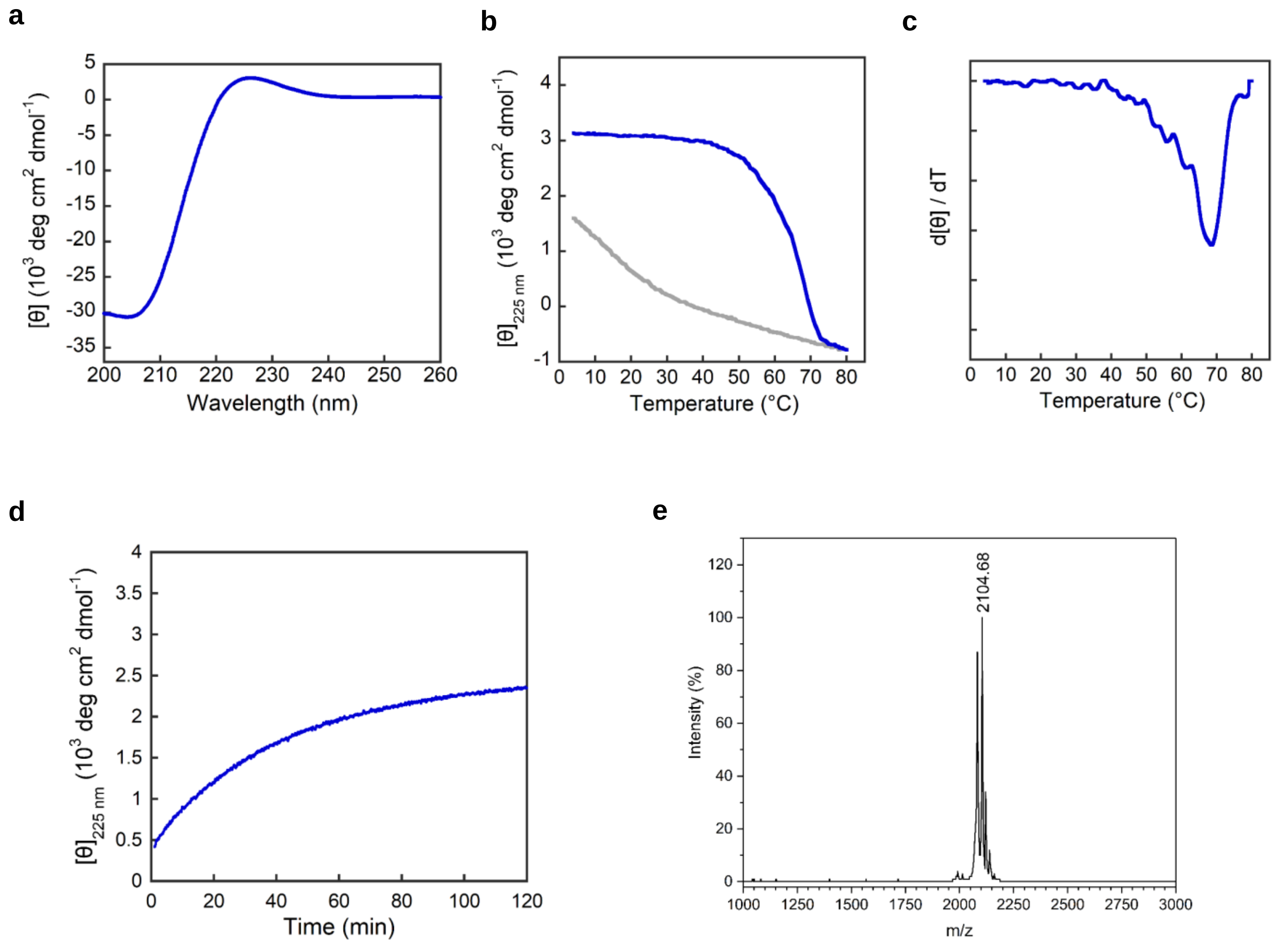

a, The CD spectrum in PBS solution (pH 7.4) at 4 °C.

b, The CD thermal unfolding (blue) and cooling (gray) curves in PBS solution.

c, The first derivative of the thermal unfolding curve, T_m = 69 °C.

d, The CD refolding curve in PBS solution at 4 °C.

e, MALDI-MS, calculated: 2104.92 $[M+Na]^+$, observed: 2104.68 $[M+Na]^+$.

N2pic3-CMP (in 1 mM HCl)

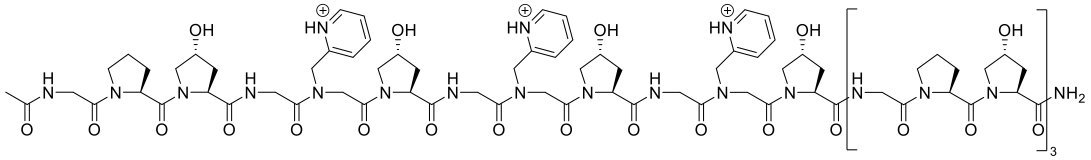

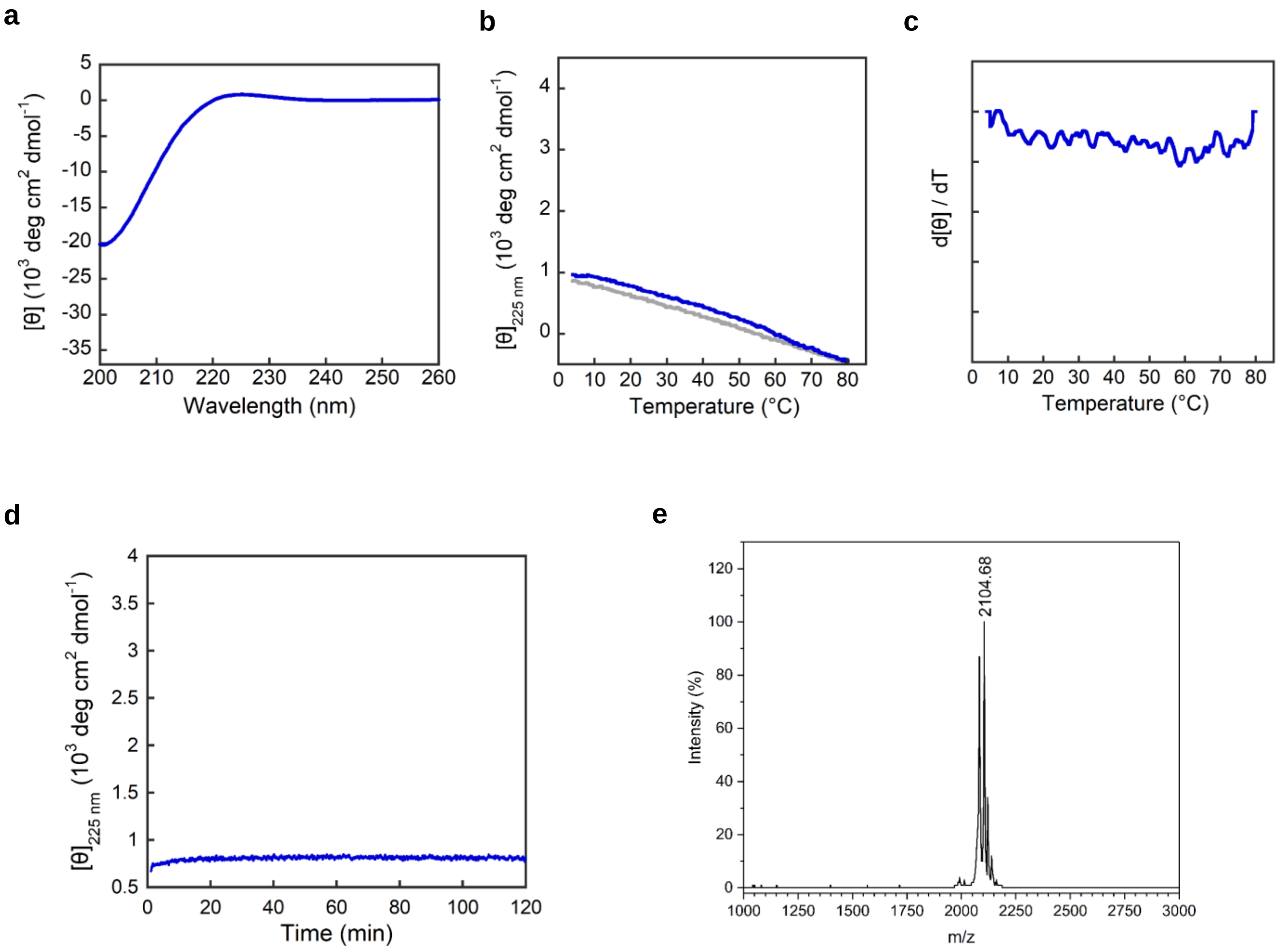

a, The CD spectrum in 1 mM HCl solution (pH 3.0) at 4 °C.

b, The CD thermal unfolding (blue) and cooling (gray) curves in 1 mM HCl solution.

c, The first derivative of the thermal unfolding curve, no triple helix.

d, The CD refolding curve in 1 mM HCl solution at 4 °C.

e, MALDI-MS, calculated: 2104.92 $[M+Na]^+$, observed: 2104.68 $[M+Na]^+$.

N2pic3-sCMP (in PBS)

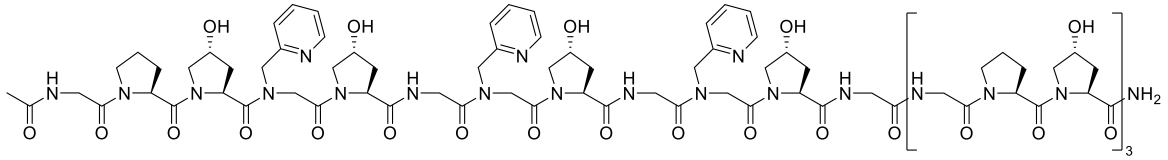

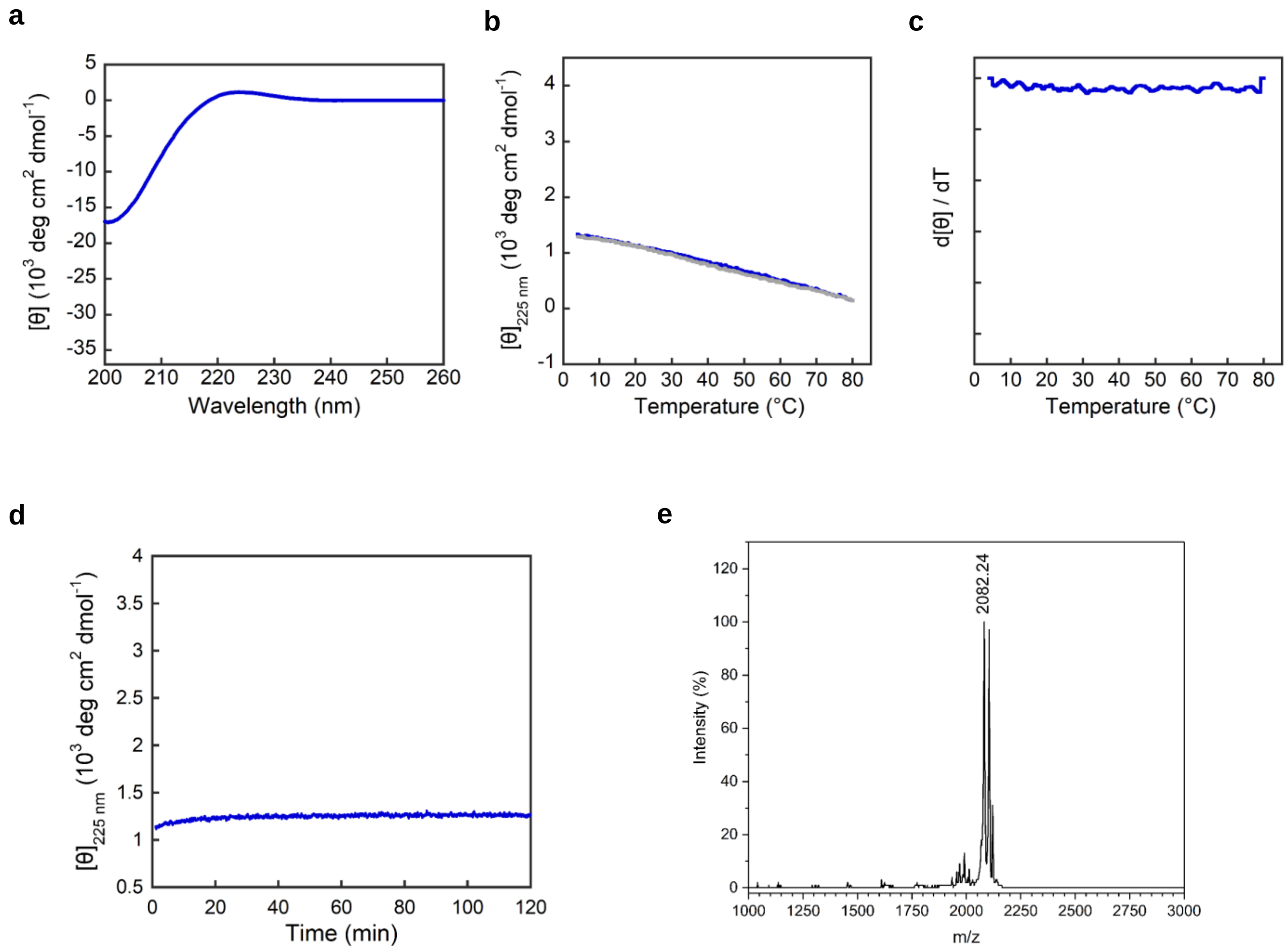

a, The CD spectrum in PBS solution (pH 7.4) at 4 °C.

b, The CD thermal unfolding (blue) and cooling (gray) curves in PBS solution.

c, The first derivative of the thermal unfolding curve, no triple helix.

d, The CD refolding curve in PBS solution at 4 °C.

e, MALDI-MS, calculated: 2082.92 $[M+H]^+$, observed: 2082.24 $[M+H]^+$.

N2pic3-sCMP (in 1 mM HCl)

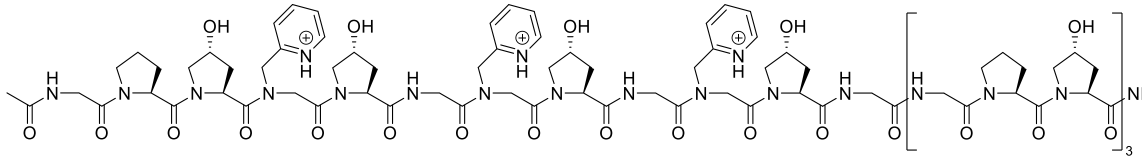

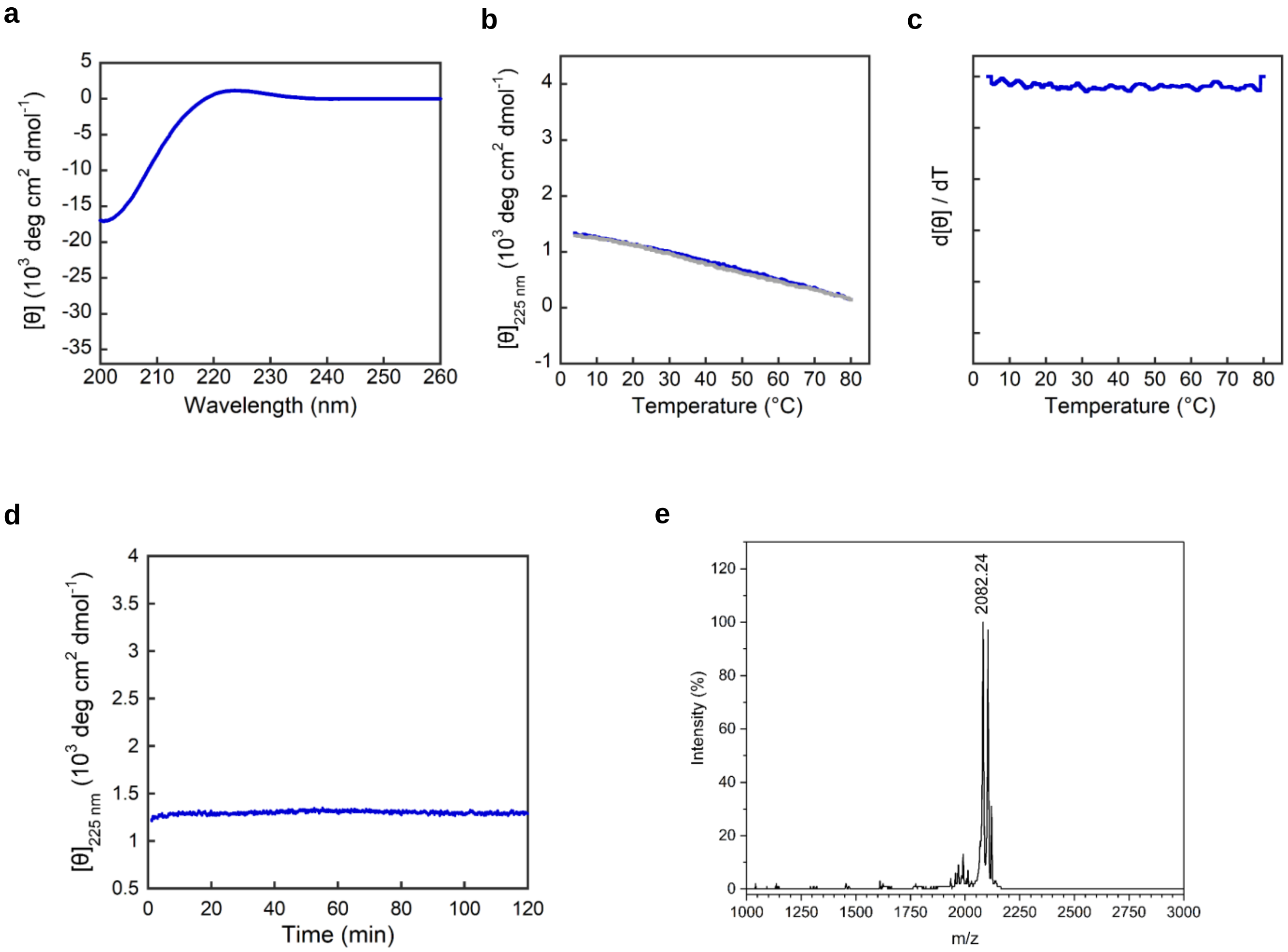

a, The CD spectrum in 1 mM HCl solution (pH 3.0) at 4 °C.

b, The CD thermal unfolding (blue) and cooling (gray) curves in 1 mM HCl solution.

c, The first derivative of the thermal unfolding curve, no triple helix.

d, The CD refolding curve in 1 mM HCl solution at 4 °C.

e, MALDI-MS, calculated: 2082.92 $[M+H]^+$, observed: 2082.24 $[M+H]^+$.

X7-CMP (in AcOH)

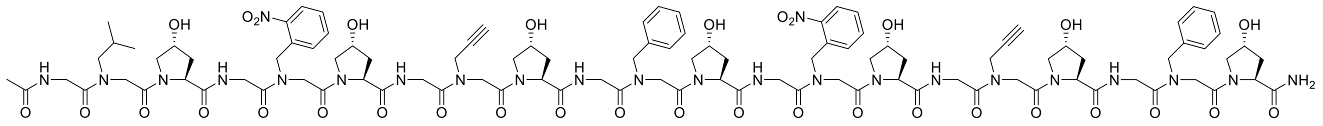

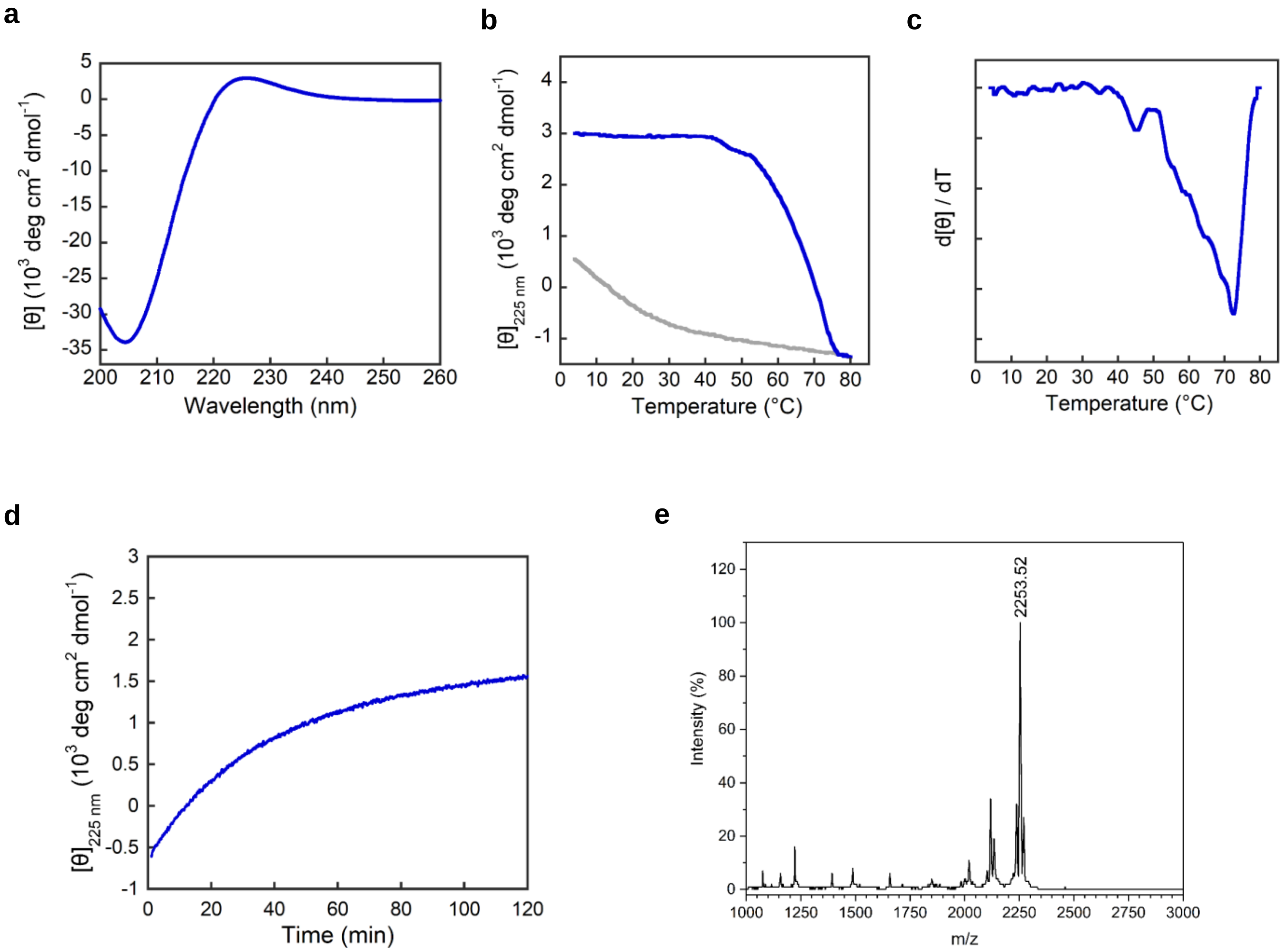

a, The CD spectrum in 50 mM AcOH solution at 4 °C.

b, The CD thermal unfolding (blue) and cooling (gray) curves in 50 mM AcOH solution.

c, The first derivative of the thermal unfolding curve, T_m = ~73 °C.

d, The CD refolding curve in 50 mM AcOH solution at 4 °C.

e, MALDI-MS, calculated: 2253.92 $[M+Na]^+$, observed: 2253.52 $[M+Na]^+$.

X7-CMP after UV exposure (in AcOH)

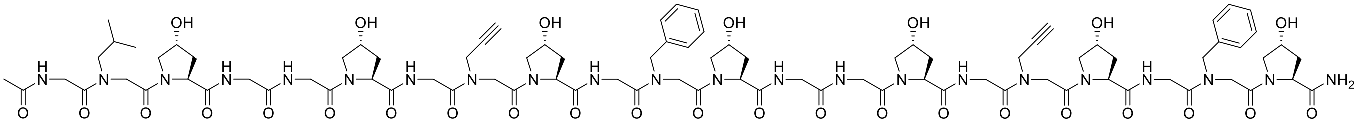

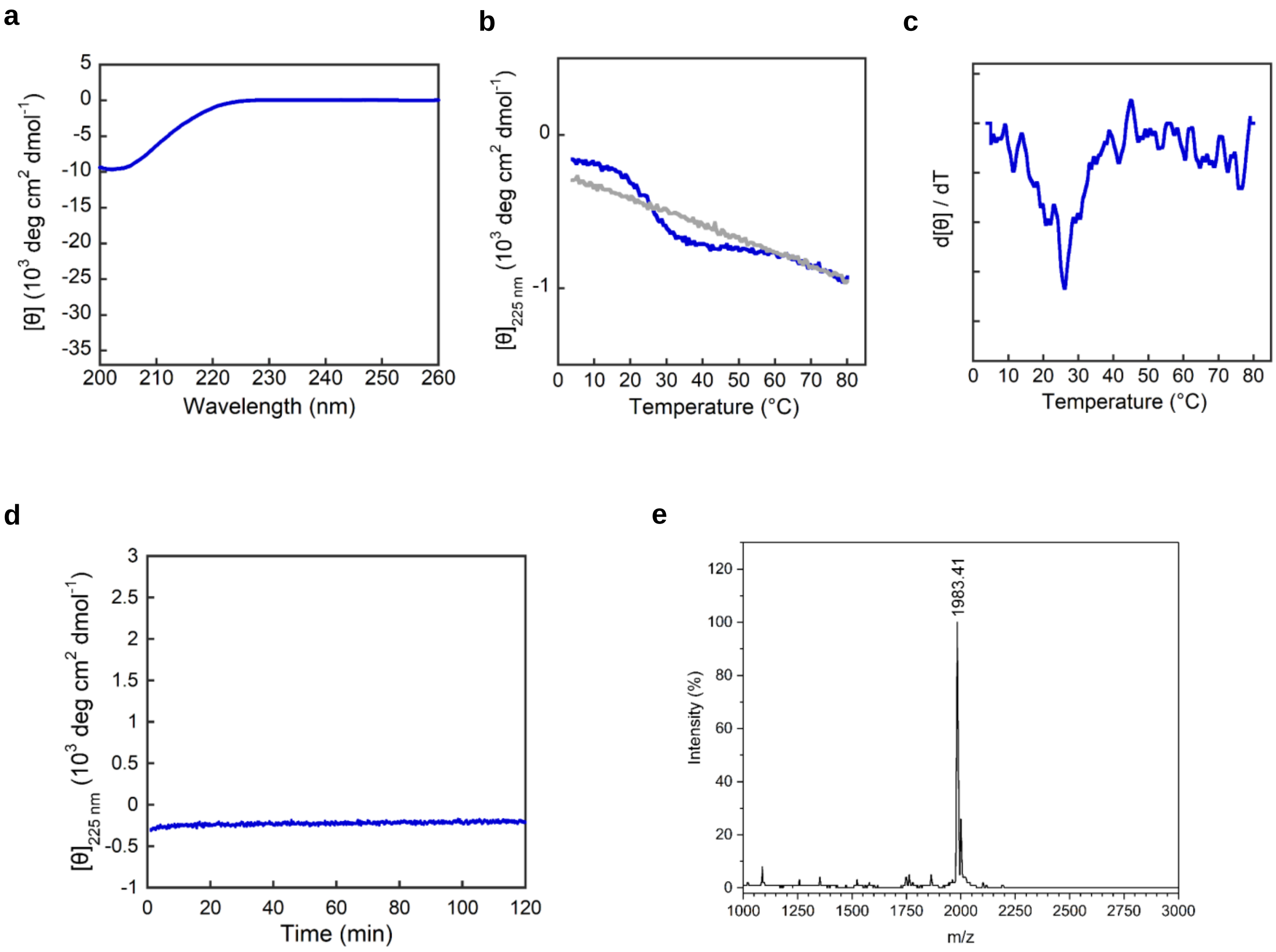

a, The CD spectrum in 50 mM AcOH solution at 4 °C.

b, The CD thermal unfolding (blue) and cooling (gray) curves in 50 mM AcOH solution.

c, The first derivative of the thermal unfolding curve, T_m = ~26 °C.

d, The CD refolding curve in 50 mM AcOH solution at 4 °C.

e, MALDI-MS, calculated: 1983.86 $[M+Na]^+$, observed: 1983.41 $[M+Na]^+$.

X-PP5 peptides

Gly-PP5

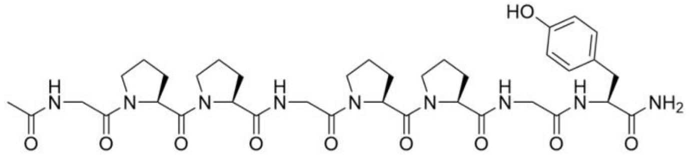

a

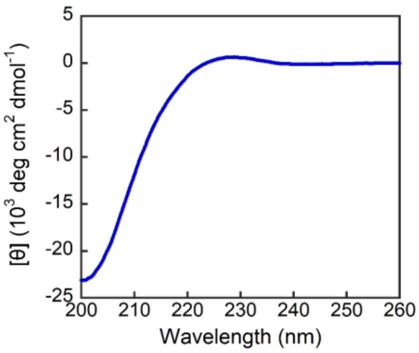

b

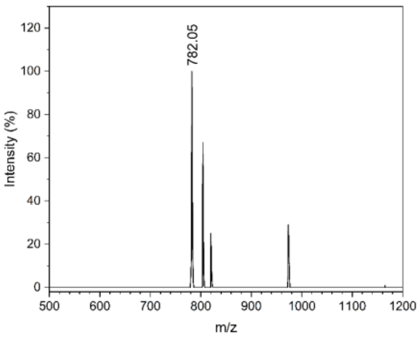

a, The CD spectrum in 5 mM phosphate buffer at 25 °C.

b, MALDI-MS, calculated: 782.38 $[M+H]^+$, observed: 782.05 $[M+H]^+$.

Asn-PP5

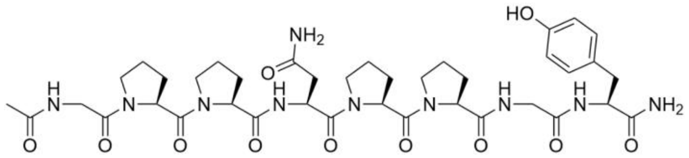

a

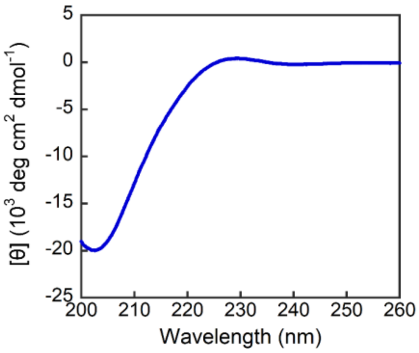

b

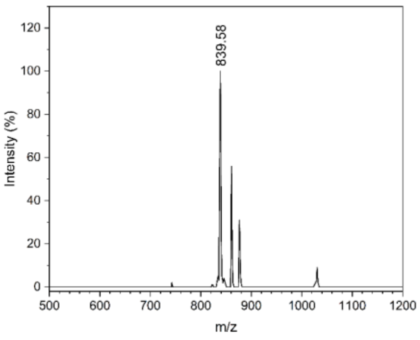

a, The CD spectrum in 5 mM phosphate buffer at 25 °C.

b, MALDI-MS, calculated: 839.40 $[M+H]^+$, observed: 839.58 $[M+H]^+$.

Lys-PP5

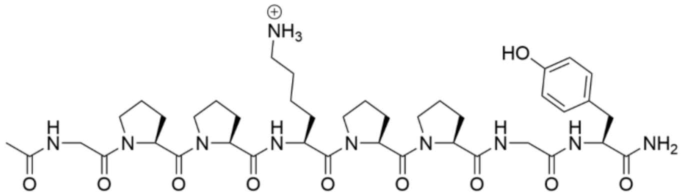

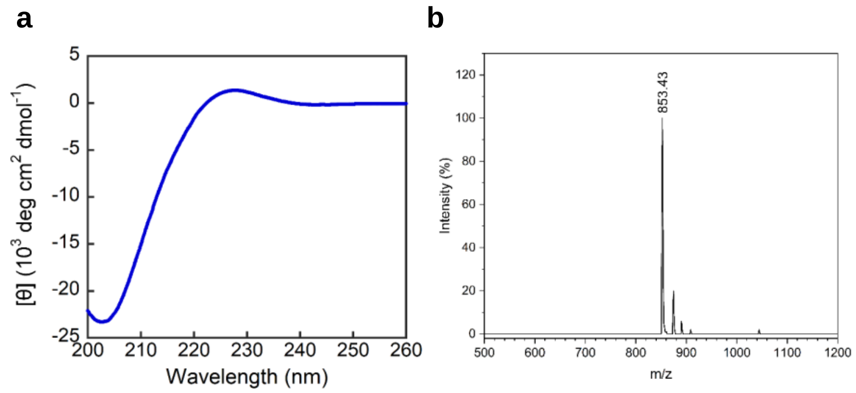

a, The CD spectrum in 5 mM phosphate buffer at 25 °C.

b, MALDI-MS, calculated: 853.45 $[M+H]^+$, observed: 853.45 $[M+H]^+$.

Ala-PP5

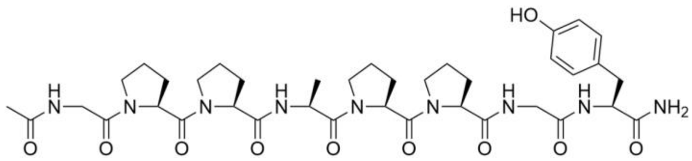

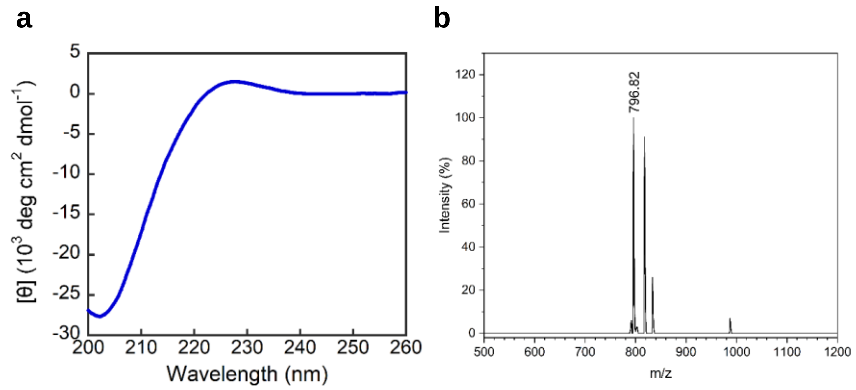

a, The CD spectrum in 5 mM phosphate buffer at 25 °C.

b, MALDI-MS, calculated: 796.39 $[M+H]^+$, observed: 796.82 $[M+H]^+$.

Leu-PP5

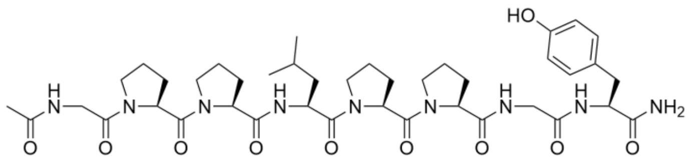

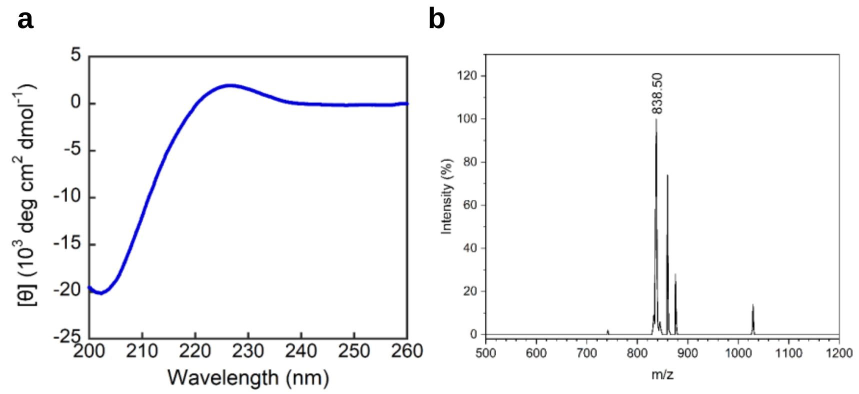

a, The CD spectrum in 5 mM phosphate buffer at 25 °C.

b, MALDI-MS, calculated: 838.44 $[M+H]^+$, observed: 838.50 $[M+H]^+$.

Nleu-PP5

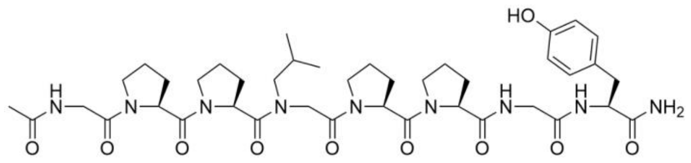

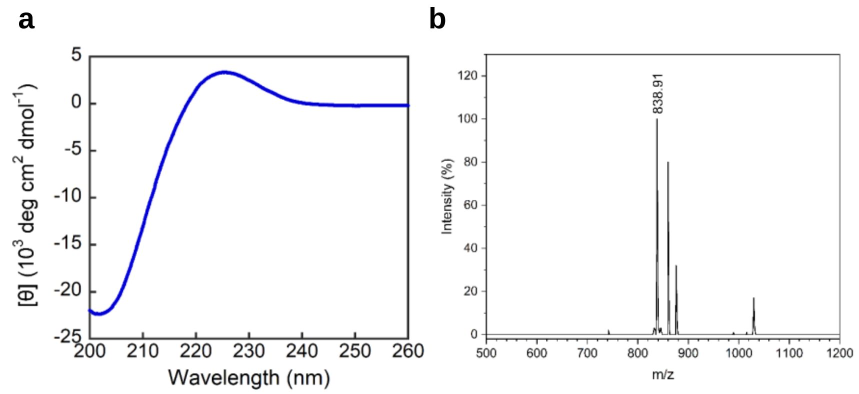

a, The CD spectrum in 5 mM phosphate buffer at 25 °C.

b, MALDI-MS, calculated: 838.44 $[M+H]^+$, observed: 838.91 $[M+H]^+$.

Nchx-PP5

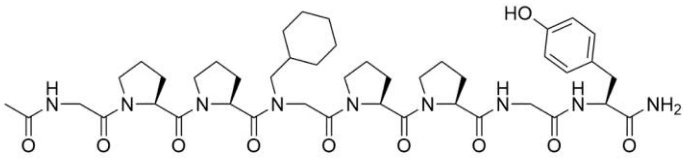

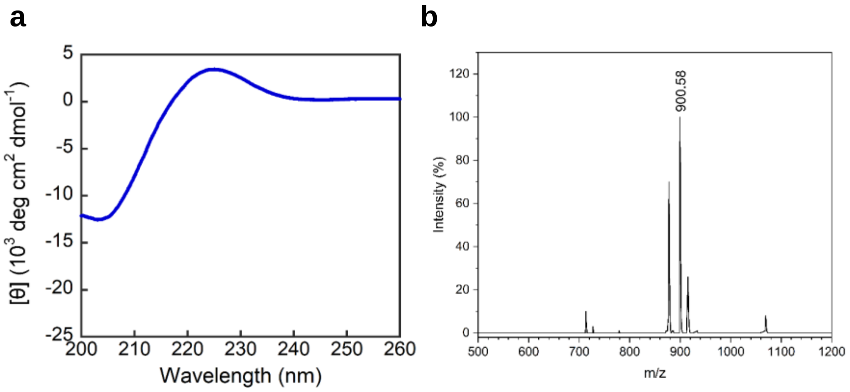

a, The CD spectrum in 5 mM phosphate buffer at 25 °C.

b, MALDI-MS, calculated: 900.47 $[M+Na]^+$, observed: 900.58 $[M+Na]^+$.

Nphe-PP5

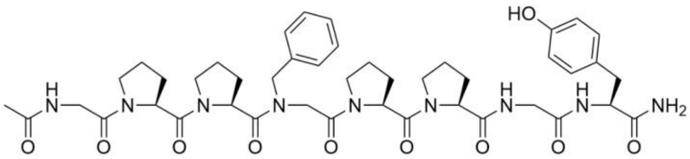

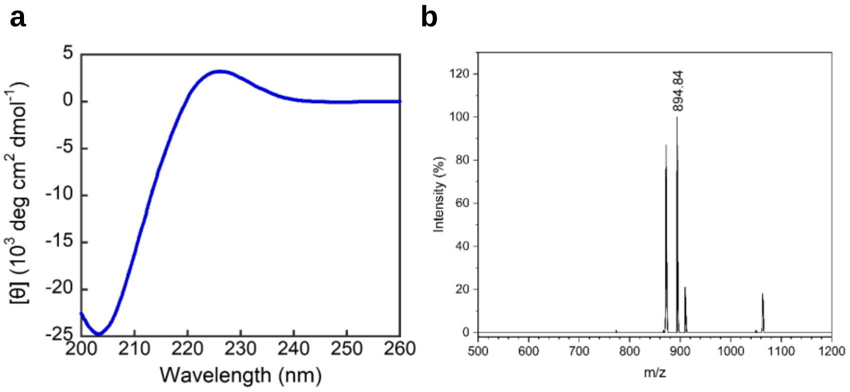

a, The CD spectrum in 5 mM phosphate buffer at 25 °C.

b, MALDI-MS, calculated: 894.42 $[M+Na]^+$, observed: 894.84 $[M+Na]^+$.

Nasn-PP5

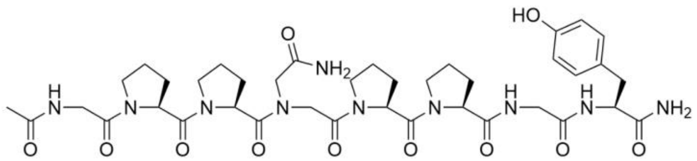

a **b**

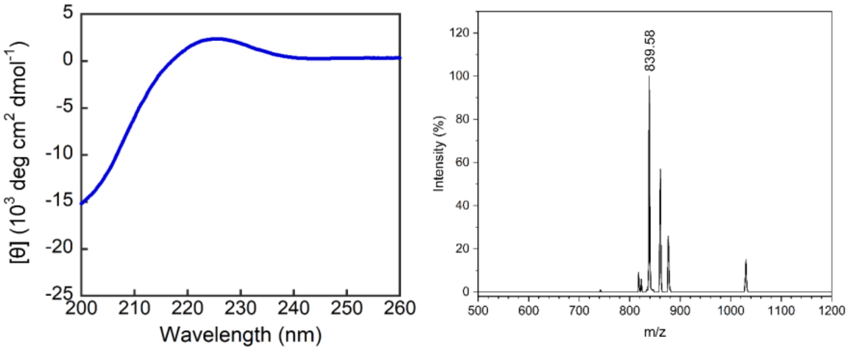

a, The CD spectrum in 5 mM phosphate buffer at 25 °C.

b, MALDI-MS, calculated: 839.40 $[M+H]^+$, observed: 839.58 $[M+H]^+$.

Nlys-PP5

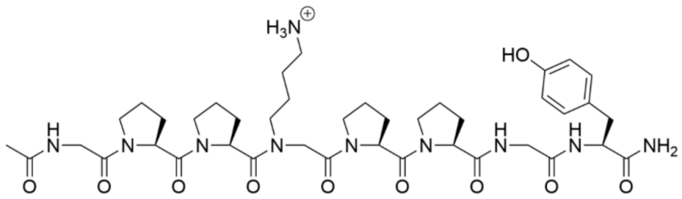

a **b**

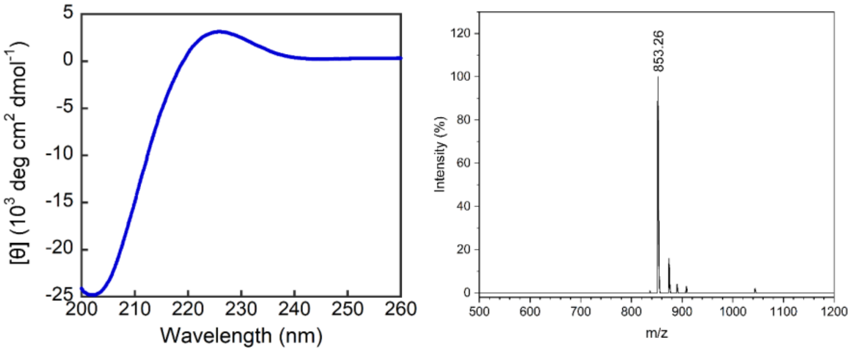

a, The CD spectrum in 5 mM phosphate buffer at 25 °C.

b, MALDI-MS, calculated: 853.45 $[M+H]^+$, observed: 853.26 $[M+H]^+$.

Pro-PP5

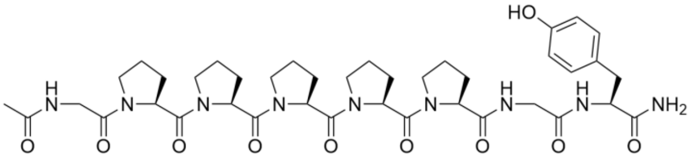

a

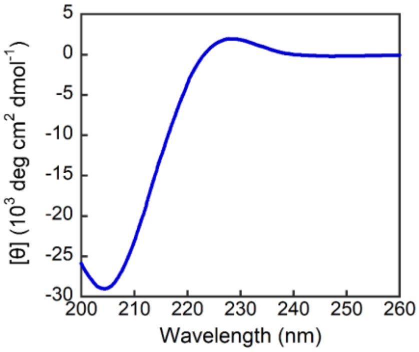

b

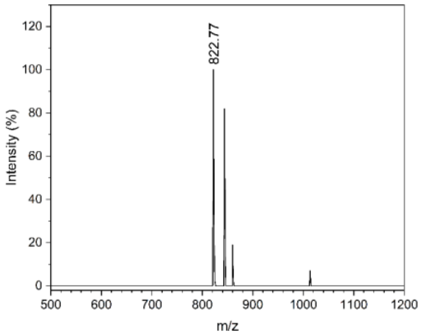

a, The CD spectrum in 5 mM phosphate buffer at 25 °C.

b, MALDI-MS, calculated: 822.41 $[M+H]^+$, observed: 822.77 $[M+H]^+$.

Hyp-PP5

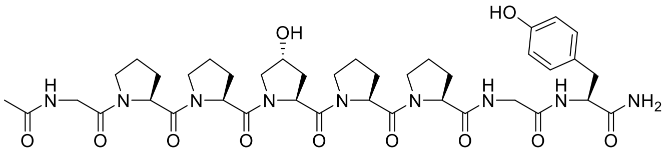

a

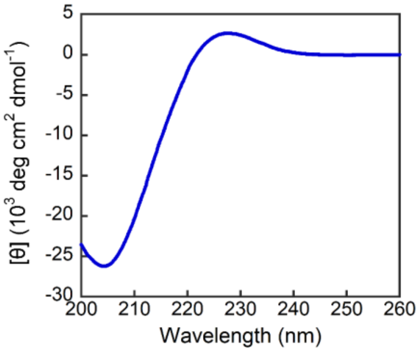

b

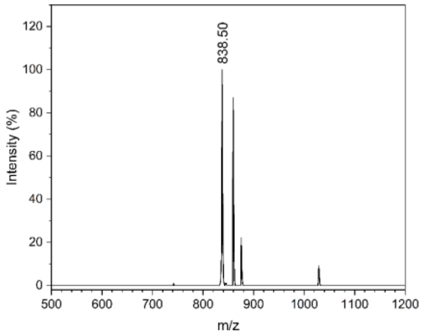

a, The CD spectrum in 5 mM phosphate buffer at 25 °C.

b, MALDI-MS, calculated: 838.40 $[M+H]^+$, observed: 838.50 $[M+H]^+$.

Flp-PP5

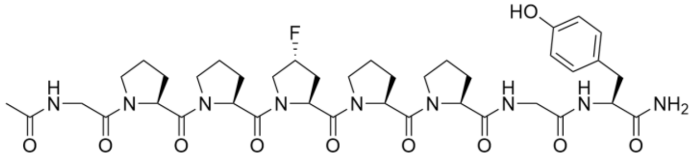

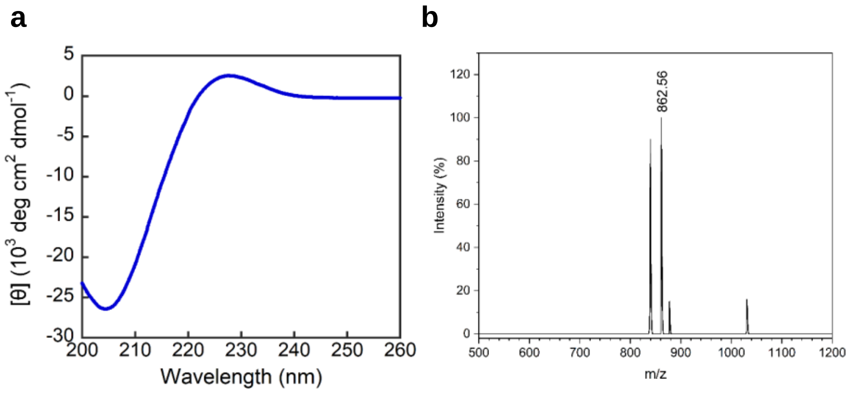

a, The CD spectrum in 5 mM phosphate buffer at 25 °C.

b, MALDI-MS, calculated: 862.39 $[M+Na]^+$, observed: 862.56 $[M+Na]^+$.

N2pic-PP5

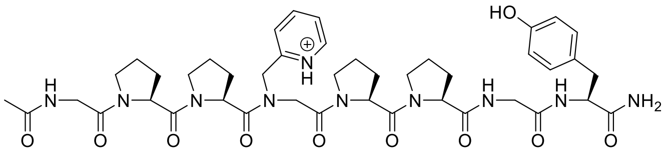

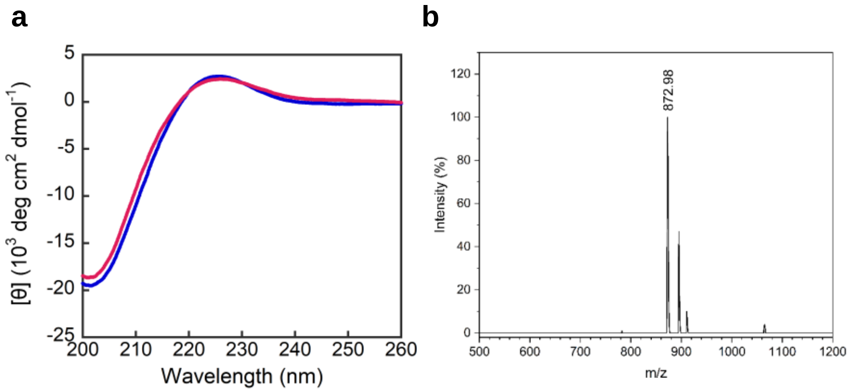

a, The CD spectrum in 1 mM HCl solution (red) and in 5 mM phosphate buffer (blue) at 25 °C.

b, MALDI-MS, calculated: 873.42 $[M+H]^+$, observed: 872.98 $[M+H]^+$.

Nme$_2$ae-PP5

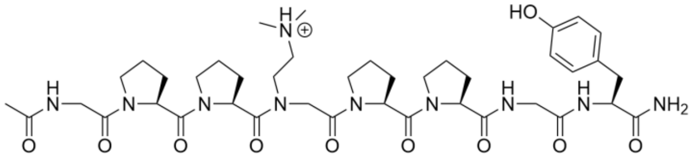

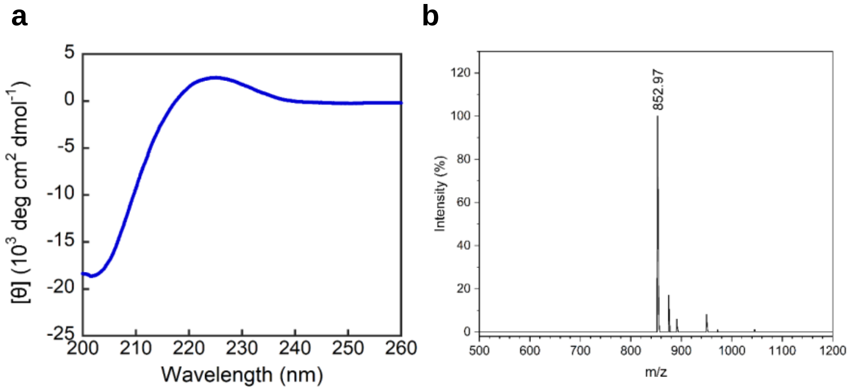

a, The CD spectrum in 5 mM phosphate buffer at 25 °C.

b, MALDI-MS, calculated: 853.45 $[M+H]^+$, observed: 852.97 $[M+H]^+$.

Net$_2$ae-PP5

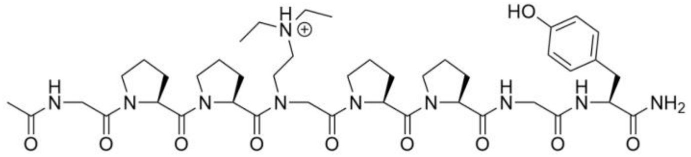

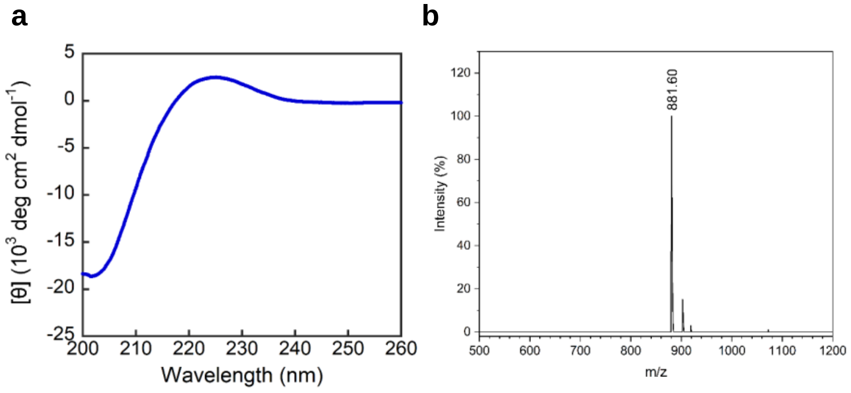

a, The CD spectrum in 5 mM phosphate buffer at 25 °C.

b, MALDI-MS, calculated: 881.49 $[M+H]^+$, observed: 881.60 $[M+H]^+$.

Nbtm⁺-PP5

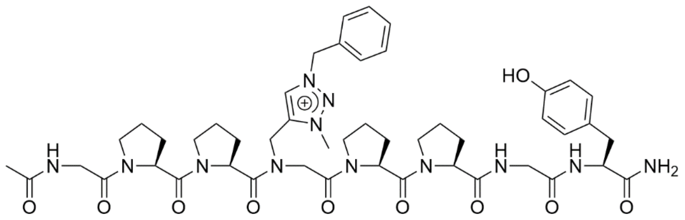

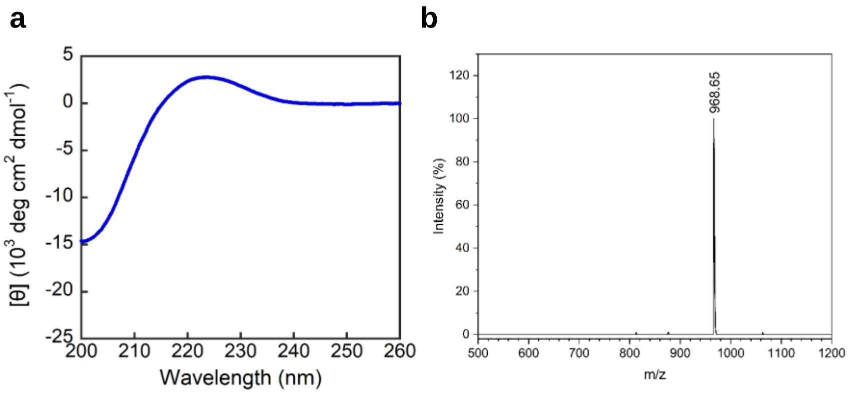

a, The CD spectrum in 5 mM phosphate buffer at 25 °C.

b, MALDI-MS, calculated: 968.48 $[M+H]^+$, observed: 968.65 $[M+H]^+$.

Nme$_3$ae-PP5

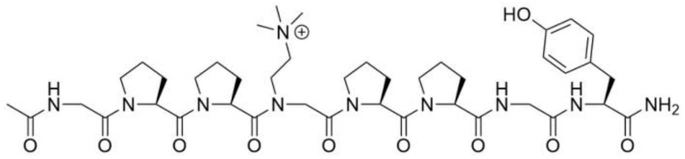

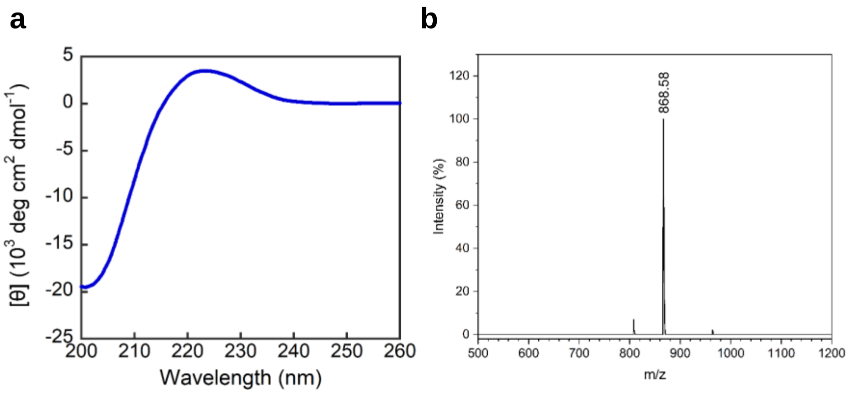

a, The CD spectrum in 5 mM phosphate buffer at 25 °C.

b, MALDI-MS, calculated: 868.47 $[M+H]^+$, observed: 868.58 $[M+H]^+$.

N^{i}pr$_2$ae-PP5

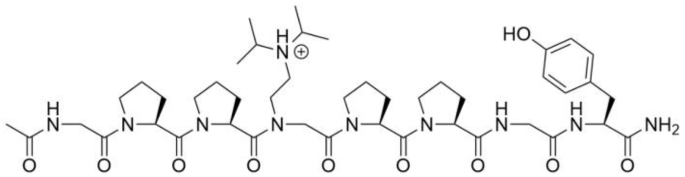

a

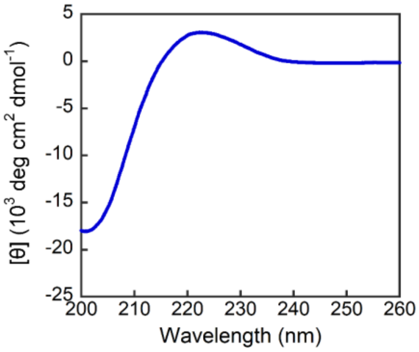

b

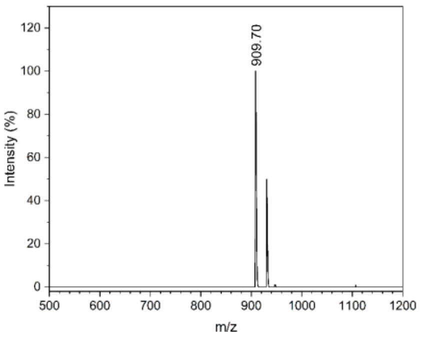

a, The CD spectrum in 5 mM phosphate buffer at 25 °C.

b, MALDI-MS, calculated: 909.52 [M+H]$^+$, observed: 909.70 [M+H]$^+$.

Supplementary References

1. Kessler, J.L., *et al.* Peptoid residues make diverse, hyperstable collagen triple-helices. *J. Am. Chem. Soc.* **143**, 10910-10919 (2021).
2. Zuckermann, R.N. Peptoid origins. *Peptide Science* **96**, 545-555 (2011).
3. Wijaya, A.W., *et al.* Cooperative intramolecular hydrogen bonding strongly enforces cis-peptoid folding. *J. Am. Chem. Soc.* **141**, 19436-19447 (2019).
4. Erdmann, R.S. & Wennemers, H. Functionalizable collagen model peptides. *J. Am. Chem. Soc.* **132**, 13957-13959 (2010).
5. Egli, J., Siebler, C., Köhler, M., Zenobi, R. & Wennemers, H. Hydrophobic moieties bestow fast-folding and hyperstability on collagen triple helices. *J. Am. Chem. Soc.* **141**, 5607-5611 (2019).
6. Lee, J. & Chmielewski, J. Folding studies of pH-dependent collagen peptides. *Chem. Biol. Drug Des.* **75**, 161-168 (2010).
7. Ackerman, M.S., *et al.* Sequence dependence of the folding of collagen-like peptides: single amino acids affect the rate of triple-helix nucleation. *J. Biol. Chem.* **274**, 7668-7673 (1999).
8. Boudko, S., *et al.* Nucleation and propagation of the collagen triple helix in single-chain and trimerized peptides: transition from third to first order kinetics. *J. Mol. Biol.* **317**, 459-470 (2002).
9. Shoulders, M.D., Kotch, F.W., Choudhary, A., Guzei, I.A. & Raines, R.T. The aberrance of the 4S diastereomer of 4-hydroxyproline. *J. Am. Chem. Soc.* **132**, 10857-10865 (2010).
10. Shoulders, M.D., Satyshur, K.A., Forest, K.T. & Raines, R.T. Stereoelectronic and steric effects in side chains preorganize a protein main chain. *Proc. Natl. Acad. Sci. U. S. A.* **107**, 559-564 (2010).
11. Kotch, F.W., Guzei, I.A. & Raines, R.T. Stabilization of the collagen triple helix by O-methylation of hydroxyproline residues. *J. Am. Chem. Soc.* **130**, 2952-2953 (2008).
12. Mizuno, K., Hayashi, T., Peyton, D.H. & Bächinger, H.P. Hydroxylation-induced stabilization of the collagen triple helix. *J. Biol. Chem.* **279**, 38072-38078 (2004).
13. Engel, J., Chen, H.-T., Prockop, D.J. & Klump, H. The triple helix ⇌ coil conversion of collagen-like polytripeptides in aqueous and nonaqueous solvents. Comparison of the thermodynamic parameters and the binding of water to (L-Pro-L-Pro-Gly)n and (L-Pro-L-Hyp-Gly)n. *Biopolymers* **16**, 601-622 (1977).
14. Nelson, M.T., *et al.* NAMD: a parallel, object-oriented molecular dynamics program. *Int J Supercomput Appl* **10**, 251-268 (1996).
15. MacKerell, A.D., Jr., *et al.* All-atom empirical potential for molecular modeling and dynamics studies of proteins. *J. Phys. Chem. B* **102**, 3586-3616 (1998).
16. Vanommeslaeghe, K., *et al.* CHARMM general force field: A force field for drug-like molecules compatible with the CHARMM all-atom additive biological force fields. *J. Comput. Chem.* **31**, 671-690 (2010).
17. Weiser, L.J. & Santiso, E.E. A CGenFF-based force field for simulations of peptoids with both cis and trans peptide bonds. *J. Comput. Chem.* **40**, 1946-1956 (2019).
18. Leach, J.P., *et al.* Hippo pathway deficiency reverses systolic heart failure after infarction. *Nature* **550**, 260-264 (2017).
19. Langer, L.B.N., *et al.* Molecular imaging of fibroblast activation protein after myocardial infarction using the novel radiotracer [^{68}Ga]MHLL1. *Theranostics* **11**, 7755-7766 (2021).
20. Liu, L., *et al.* Molecular imaging of collagen destruction of the spine. *ACS Nano* **15**, 19138-19149 (2021).
21. Jing, D., *et al.* Tissue clearing of both hard and soft tissue organs with the PEGASOS method. *Cell Res.* **28**, 803-818 (2018).
22. Egli, J., Schnitzer, T., Dietschreit, J.C.B., Ochsenfeld, C. & Wennemers, H. Why proline? Influence of ring-size on the collagen triple helix. *Org. Lett.* **22**, 348-351 (2020).

23. Hodges, J.A. & Raines, R.T. Stereoelectronic effects on collagen stability: the dichotomy of 4-fluoroproline diastereomers. *J. Am. Chem. Soc.* **125**, 9262-9263 (2003).
24. Gorske, B.C., Bastian, B.L., Geske, G.D. & Blackwell, H.E. Local and tunable $n \rightarrow \pi^*$ interactions regulate amide isomerism in the peptoid backbone. *J. Am. Chem. Soc.* **129**, 8928-8929 (2007).
25. Chamorro-Arenas, D., Osorio-Nieto, U., Quintero, L., Hernández-García, L. & Sartillo-Piscil, F. Selective, catalytic, and dual C(sp3)–H oxidation of piperazines and morpholines under transition-metal-free conditions. *J. Org. Chem.* **83**, 15333-15346 (2018).
