# Supplementary material for: Cis-trans isomerization of peptoid residues in the collagen triple-helix
Source: Nat Commun. 2023 Nov 21;14:7571. doi: 10.1038/s41467-023-43469-8 (PMC10663571; doi:10.1038/s41467-023-43469-8)
Supplement: Supplementary file 3 — Description of Additional Supplementary Files [file 41467_2023_43469_MOESM3_ESM.pdf]

Title: Supplementary Movie 1

Description: Light sheet fluorescence microscopy scanning of a mouse heart 10 days after myocardial infarction (MI) showing Cy5- N2pic3-CMP's in vivo binding to denatured collagen within the MI fibrotic scar (yellow); the mouse was intravenously injected with Cy5-N2pic3-CMP one hour before its heart was harvested for tissue clearing (scale bar: 1 mm).
