# Supplementary material for: Cis-trans isomerization of peptoid residues in the collagen triple-helix
Source: Nat Commun. 2023 Nov 21;14:7571. doi: 10.1038/s41467-023-43469-8 (PMC10663571; doi:10.1038/s41467-023-43469-8)
Supplement: Supplementary file 5 — Reporting Summary [file 41467_2023_43469_MOESM5_ESM.pdf]

nature portfolio

Corresponding author(s): Yang Li

Last updated by author(s): Nov 6, 2023

Reporting Summary

Nature Portfolio wishes to improve the reproducibility of the work that we publish. This form provides structure for consistency and transparency in reporting. For further information on Nature Portfolio policies, see our Editorial Policies and the Editorial Policy Checklist.

Statistics

For all statistical analyses, confirm that the following items are present in the figure legend, table legend, main text, or Methods section.

n/a	Confirmed	
☐	☒	The exact sample size (*n*) for each experimental group/condition, given as a discrete number and unit of measurement
☐	☒	A statement on whether measurements were taken from distinct samples or whether the same sample was measured repeatedly
☐	☒	The statistical test(s) used AND whether they are one- or two-sided<br>*Only common tests should be described solely by name; describe more complex techniques in the Methods section.*
☒	☐	A description of all covariates tested
☐	☒	A description of any assumptions or corrections, such as tests of normality and adjustment for multiple comparisons
☐	☒	A full description of the statistical parameters including central tendency (e.g. means) or other basic estimates (e.g. regression coefficient) AND variation (e.g. standard deviation) or associated estimates of uncertainty (e.g. confidence intervals)
☐	☒	For null hypothesis testing, the test statistic (e.g. *F*, *t*, *r*) with confidence intervals, effect sizes, degrees of freedom and *P* value noted<br>*Give P values as exact values whenever suitable.*
☒	☐	For Bayesian analysis, information on the choice of priors and Markov chain Monte Carlo settings
☒	☐	For hierarchical and complex designs, identification of the appropriate level for tests and full reporting of outcomes
☒	☐	Estimates of effect sizes (e.g. Cohen's *d*, Pearson's *r*), indicating how they were calculated

Our web collection on statistics for biologists contains articles on many of the points above.

Software and code

Policy information about availability of computer code

Data collection	Circular dichroism data was acquired using a JASCO J-1500 CD Spectra Manager (Version 2.15.01). Differential scanning calorimetry data was acquired using a Automated MicroCal PEAQ-DSC instrument. Molecular simulation was performed using a NAMD package (v2.12) in the local Linux workstation with a CHARMM27 force field for collagen peptides and a modified CHARMM General Force field for the peptoid substitution. In-vivo imaging data was acquired using an Living Image Software (Version 4.5.5). HE and fluorescence micrographs were acquired using an EVOS M7000 imaging system Software (Version 2.1.677.717). Light sheet microscopy fluorescence images were acquired using a Imspector Main Application (Version 7.3.7). Mass spectra were obtained on a Shimadzu 8020 matrix-aided laser analytical ionization-time of flight mass spectrometer (ICS version 2.7.0.1245).
Data analysis	Melting temperature data was analyzed using the JASCO Spectra Manager software (Version 2.10.05). In-vivo fluorescence images were analyzed using Living Image Software (Version 4.5.5). NMR data was analyzed by MestReNova (Version: 14.0.0-23239). Data analysis used one-way ANOVA with post-hoc Tukey HSD Calculator, see website below: https://astatsa.com/OneWay_Anova_with_TukeyHSD/.

For manuscripts utilizing custom algorithms or software that are central to the research but not yet described in published literature, software must be made available to editors and reviewers. We strongly encourage code deposition in a community repository (e.g. GitHub). See the Nature Portfolio guidelines for submitting code & software for further information.

Data

Policy information about availability of data

All manuscripts must include a data availability statement. This statement should provide the following information, where applicable:

- Accession codes, unique identifiers, or web links for publicly available datasets
- A description of any restrictions on data availability
- For clinical datasets or third party data, please ensure that the statement adheres to our policy

The data generated in this study are provided in the Supplementary Information and the Source Data file. Any additional requests for information can be directed to, and will be fulfilled by, the corresponding authors.

Research involving human participants, their data, or biological material

Policy information about studies with human participants or human data. See also policy information about sex, gender (identity/presentation), and sexual orientation and race, ethnicity and racism.

Reporting on sex and gender	Our study does not involve human participants, data, or biological materials.
Reporting on race, ethnicity, or other socially relevant groupings	Our study does not involve human participants, data, or biological materials.
Population characteristics	Our study does not involve human participants, data, or biological materials.
Recruitment	Our study does not involve human participants, data, or biological materials.
Ethics oversight	Our study does not involve human participants, data, or biological materials.

Note that full information on the approval of the study protocol must also be provided in the manuscript.

Field-specific reporting

Please select the one below that is the best fit for your research. If you are not sure, read the appropriate sections before making your selection.

☒ Life sciences ☐ Behavioural & social sciences ☐ Ecological, evolutionary & environmental sciences

For a reference copy of the document with all sections, see nature.com/documents/nr-reporting-summary-flat.pdf

Life sciences study design

All studies must disclose on these points even when the disclosure is negative.

Sample size	No statistical methods used to predetermine sample size. Sample sizes were determined on the basis of previous studies (Ref: ACS Nano. 2021;15(12):19138-19149; Nat Commun. 2023;14(1):6983). All in vivo experiments were performed with 3 independent animals, which is sufficient to draw qualitative conclusions. The investigators were not blinded to group allocation during data collection and analysis.
Data exclusions	No data were excluded from the analyses.
Replication	All in vitro tests were confirmed at least two times and all in vivo experiments were confirmed three times independently. All attempts at replication were successful.
Randomization	All allocation was random.
Blinding	During the data collection and analysis process, the investigators did not blind the group assignments because the results were obvious and only used for qualitative analysis.

Reporting for specific materials, systems and methods

We require information from authors about some types of materials, experimental systems and methods used in many studies. Here, indicate whether each material, system or method listed is relevant to your study. If you are not sure if a list item applies to your research, read the appropriate section before selecting a response.

Materials & experimental systems

n/a	Involved in the study	
[ ]	[x]	Antibodies
[x]	[ ]	Eukaryotic cell lines
[x]	[ ]	Palaeontology and archaeology
[ ]	[x]	Animals and other organisms
[x]	[ ]	Clinical data
[x]	[ ]	Dual use research of concern
[x]	[ ]	Plants

Methods

n/a	Involved in the study	
[x]	[ ]	ChIP-seq
[x]	[ ]	Flow cytometry
[x]	[ ]	MRI-based neuroimaging

Antibodies

Antibodies used

1. Anti-Collagen I antibody: rabbit polyclonal to collagen I, Abcam, catalog number: ab34710, lot number: GR3432109-2.
2. Cy™3 AffiniPure Goat Anti-Rabbit IgG (H+L), Jackson, catalog number: 111-165-003, lot number: 159083.

Validation

Anti-Collagen I antibody, Abcam (ab34710), and Rabbit polyclonal to Collagen I have been successfully validated by the suppliers and by others, e.g., on cryosections of mouse embryos, see reference below (Hwang J, Huang Y, Burwell TJ, et al. In Situ Imaging of Tissue Remodeling with Collagen Hybridizing Peptides. ACS Nano. 2017;11(10):9825-9835).

Animals and other research organisms

Policy information about studies involving animals; ARRIVE guidelines recommended for reporting animal research, and Sex and Gender in Research

Laboratory animals

BALB/c nude mouse, female, 8–12 weeks old. Myocardial infarction model mouse, C57BL/6J, male, 8 weeks old.
Mice were housed in the pathogen-free room with a temperature of 20-26 °C and a humidity of 40-70%, a 12-h light/12-h dark cycle.

Wild animals

The study did not involve wild animals.

Reporting on sex

We believed that findings in this study apply to both female and male mouse. We chose the sex of mice based on previous studies.

Field-collected samples

This study does not involve field-collected samples.

Ethics oversight

Experimental animal use and ethics committee of the Fifth Affiliated Hospital of Sun Yat-sen University.

Note that full information on the approval of the study protocol must also be provided in the manuscript.
